# Opportunities and Limitations of Ionic Liquid‐ and Organic Carbonate Solvent‐Based Electrolytes for Mg‐Ion‐Based Dual‐Ion Batteries

**DOI:** 10.1002/cssc.202101227

**Published:** 2021-09-02

**Authors:** Verena Küpers, Jan Frederik Dohmann, Peter Bieker, Martin Winter, Tobias Placke, Martin Kolek

**Affiliations:** ^1^ MEET Battery Research Center Institute of Physical Chemistry University of Münster Corrensstraße 46 48149 Münster Germany; ^2^ Helmholtz Institute Münster (HI MS), IEK-12 Forschungszentrum Jülich GmbH Corrensstrasse 46 48149 Münster Germany

**Keywords:** dual-ion batteries, electrolyte additives, electrolytes, energy storage, rechargeable Mg batteries

## Abstract

Dual‐ion batteries (DIBs) offer a great alternative to state‐of‐the‐art lithium‐ion batteries, based on their high promises due to the absence of transition metals and the use of low‐cost materials, which could make them economically favorable targeting stationary energy storage applications. In addition, they are not limited by certain metal cations, and DIBs with a broad variety of utilized ions could be demonstrated over the last years. Herein, a systematic study of different electrolyte approaches for Mg‐ion‐based DIBs was conducted. A side‐by‐side comparison of Li‐ and Mg‐ion‐based electrolytes using activated carbon as negative electrode revealed the opportunities but also limitations of Mg‐ion‐based DIBs. Ethylene sulfite was successfully introduced as electrolyte additive and increased the specific discharge capacity significantly up to 93±2 mAh g^−1^ with coulombic efficiencies over 99 % and an excellent capacity retention of 88 % after 400 cycles. In addition, and for the first time, highly concentrated carbonate‐based electrolytes were employed for Mg‐ion‐based DIBs, showing adequate discharge capacities and high coulombic efficiencies.

## Graphical abstract

**Dual‐ion batteries**: Different electrolyte approaches including ionic liquids, additives, and highly concentrated electrolytes for Mg‐ion‐based dual‐ion batteries with graphite as positive and activated carbon as negative electrodes are investigated. Combining the improved electrolyte formulation with an optimized cut‐off potential and specific current, specific discharge capacities of up to 93±2 mAh g^−1^ with a capacity retention of 88 % after 400 cycles are achieved.

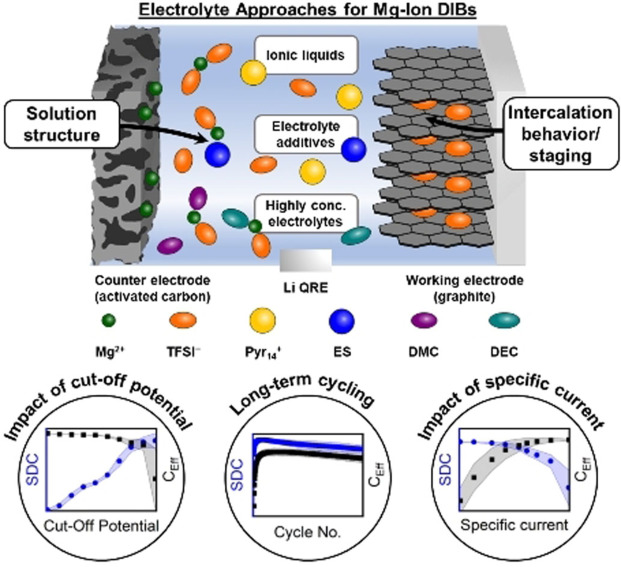

## Introduction

The generation of electric energy needs to undergo drastic changes. While economically friendly technologies to generate electricity, such as solar power and wind energy, are already present, their discontinuity raises the need for large‐scale short‐term energy storage systems. State‐of‐the‐art lithium‐ion batteries (LIBs; a list of abbreviations is given in Table S1) currently offer an optimum compromise between high energy density and power, long lifetime, and low cost for various application areas.^[1]^ Still, a technology monopoly should be avoided, as it may involve the dependence on (future) critical materials.^[2]^

One alternative to Li‐based systems is the utilization of magnesium in alternative battery technologies. In theory, Mg metal has a high capacity (2205 mAh g^−1^ and 3833 mAh cm^−3^) based on its double charge and an adequate potential of −2.36 V vs. standard hydrogen electrode (SHE) for Mg|Mg^2+^.^[3]^ However, until now, mainly cathodes with low potentials and comparably low capacities are known to be used in rechargeable Mg batteries (RMBs), as the high charge density of the Mg^2+^ ion reduces or even eliminates its mobility in solid ion conductors.[^3^, ^4^] When combined with the commonly used Mg metal[^4a^, ^5^] or sometimes Mg alloy (e. g., with bismuth)^[6]^ anode materials in RMBs, such cells result in ≤2.5 V mean cell voltage for discharge. A low cell voltage will inevitably result in the necessity to assemble more cells in series to achieve the desired battery module/pack voltage. New high‐voltage cathodes for Mg are therefore needed to realize battery cells with a high cell voltage and resulting battery packs with a confined number of cells.^[5a]^

An alternative to common insertion‐ (e. g., layered oxides or sulfides)[^5c^, ^6d^, ^7^] or conversion‐type (e. g., sulfur)^[8]^ cathodes is the usage of an anion intercalating graphite as positive electrode (cathode). The dual‐ion battery (DIB) energy storage mechanism is different from the “ion transfer” mechanism, where the cation is transported from the cathode through the electrolyte to the negative electrode (anode) and back during charge and discharge, respectively. Instead, the cation is de‐/intercalated on the anode side, while the anion is simultaneously de‐/intercalated on the cathode side. This mechanism could be enabled, for example, in carbonate‐,^[9]^ ionic liquid‐,^[10]^ and even water‐based^[11]^ electrolytes with numerous different cations,[^9c^, ^10b^, ^12^] anions,[^9a^, ^13^] anodes,[^9b^, ^10^, ^14^] and cathodes.^[15]^ Anion intercalation can also take place in LIBs.^[16]^

The dual‐ion system is also highly applicable for Mg‐ion‐based systems, as Mg^2+^‐transport within the cathode (as known for classical RMB) is not a limiting factor. In addition, the high operating potential for anion intercalation into graphite (≈4.4–5.2 V vs. Li|Li^+^; 3.7–4.5 V vs. Mg|Mg^2+^ can be expected)^[17]^ could result in a significantly increased cell voltage compared to commonly investigated RMB systems.[^5c^, ^6d^, ^7^] However, only few reports show that Mg‐ion‐based DIB cell chemistries with graphite can be enabled, even though most of them do not use high‐capacity Mg metal anodes, yielding mean cell voltages below 3 V.[^12i^, ^12j^, ^12k^] This results from the inability to electrochemically dissolve and deposit Mg with reasonable reversibility in known electrolytes for DIBs, such as ionic liquid‐[^10^, ^17^] or carbonate‐based[^9a^, ^12c^] electrolytes. Electrolytes based on ionic liquids or carbonates tend to passivate the Mg surface and result in high overpotentials for Mg electrodissolution/‐deposition.^[18]^ Approaches of electrolytes based on ionic liquids, which enable reversible Mg electrodeposition/‐dissolution, either use mixtures with oxidatively less stable ether solvents,^[19]^ make use of Mg(BH_4_)_2_ salt,^[20]^ showing limited oxidative stability, or could not be reproduced[^18c^, ^18d^, ^18e^, ^21^] by other groups. Other electrolyte types commonly used for RMBs contain ethers and often additional corrosive species, such as chlorides, and show limited oxidative stabilities.[^19d^, ^22^] Further approaches contain large anions [e. g., monocarborane (CB_11_H_11_^−^)[^22c^, ^23^] or borates^[24]^ like tetrakis(hexafluoroisopropyloxy) borate, [B(hfip)_4_]^−^], which are likely not able to intercalate into graphite^[13a]^ and have a limited oxidative stability as well.

Recently, Seggem et al. reported a Mg powder‐based DIB with a graphite cathode using 0.1 m Mg trifluoromethanesulfonate [Mg(OTf)_2_] in 1‐butyl‐3‐methyl‐imidazolium hexafluorophosphate (BMIMPF_6_).^[25]^ Even though reversible Mg electrodeposition and ‐dissolution of Mg(OTf)_2_ in 1‐butyl‐3‐methylimidazolium tetrafluoroborate (BMIMBF_4_) has been reported,^[21]^ no reversible Mg electrodeposition/‐dissolution could be observed from Mg(OTf)_2_ in BMIMPF_6_ by others so far,^[26]^ likely based on the Mg‐passivating nature of PF_6_^−^, if no additives such as chlorides are added.^[27]^ In addition, reversible Mg electrodeposition/‐dissolution from ionic liquids with BMIM^+^ could not be reproduced by other groups.[^18c^, ^18d^, ^18e^] The observed redox activities were rather correlated with the redox activity of the ionic liquid.^[18e]^ In addition, the authors observe only a slight shift of the (002) reflection of graphite upon charging,^[25]^ however, no splitting of the reflection, which is known to indicate a staging behavior and proving anion intercalation into graphite.[^13b^, ^17^, ^28^] Further research is therefore needed to prove if Mg(OTf)_2_ in BMIMPF_6_ is really suitable for Mg electrodeposition/‐dissolution on the anode [e. g., by scanning electron microscopy (SEM), energy‐dispersive X‐ray spectroscopy (EDX), and X‐ray diffraction (XRD)^[27]^] and PF_6_^−^‐intercalation in a graphite cathode in Mg metal‐based DIBs, or if other reactions are responsible for the observed electrochemical activity.

**Figure 1:**
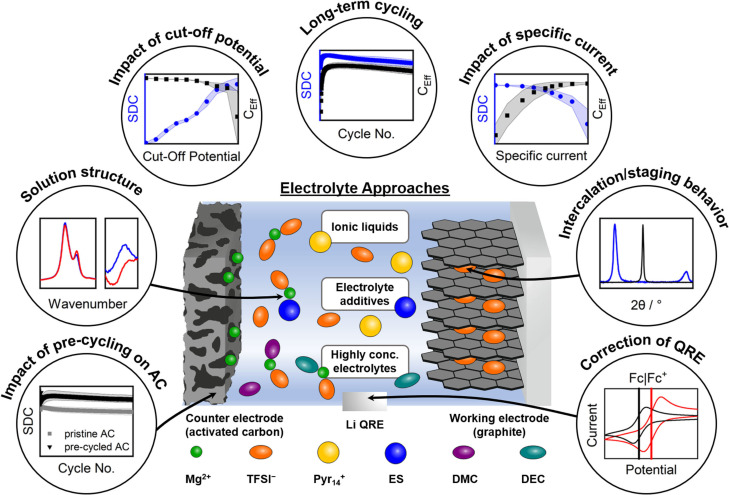
Schematic overview of the cells and investigations presented in this work. Please note that the coordination of different molecules indicated here does not reflect the quantitative coordination within the electrolyte. SDC=specific discharge capacity, C_Eff_=coulombic efficiency, AC=activated carbon, QRE=quasi reference electrode, TFSI^−^=bis(trifluoromethanesulfonyl)imide anion, ES=ethylene sulfite, DMC=dimethyl carbonate, DEC=diethyl carbonate.

The here reported study aims to gain deeper insights into the bis(trifluoromethanesulfonyl)imide anion (TFSI^−^) intercalation into graphite from a Mg‐ion‐based electrolyte compared to a Li‐ion‐based system. In addition to a commonly used ionic liquid‐based electrolyte, further electrolyte concepts based on highly concentrated electrolytes,[^9a^, ^12c^] as well as the use of ethylene sulfite (ES) as electrolyte additive,[^12a^, ^12b^, ^14^, ^29^] are evaluated based on their ability to improve the overall cycling performance. Since the electrolytes studied herein do not enable Mg metal electrodeposition/‐dissolution at an adequate overpotential, all DIB cells presented herein are based on activated carbon (AC) as counter electrode (CE). Even though AC does not show a capacity based on intercalation or conversion reactions, the formation of a double layer on its surface enables physical storage of charges in form of capacitance.^[30]^ This double layer formation shows fast kinetics and is usually within the electrochemical stability window of the electrolyte, thus, allowing cathodes to be evaluated with electrolytes, which are not able to electrodeposit and ‐dissolve Mg at adequate overpotentials if the cathode potential is controlled with a reference electrode.[^12k^, ^30^, ^31^] In addition, Li metal was used as reference electrode (RE, for Li‐ion‐based cells) or quasi‐reference electrode (QRE, for Mg‐ion‐based cells), since the usage of a Mg|Mg^2+^ electrode can lead to high variations of the working electrode (WE) potential.[^12k^, ^30^] A scheme of the cell setup including the three different electrolyte approaches (ionic liquids, electrolyte additives, and highly concentrated carbonate‐based electrolytes) as well as an overview of the investigated effects is given in Figure 1. Besides the long‐term cycling performance of graphite ‖ AC cells with these electrolytes, their limitations, but also opportunities regarding the cut‐off potentials and specific currents are discussed. Differences in the cycling performance are evaluated based on the TFSI^−^ intercalation behavior, but also variations in the solution structure and possible resulting influences. In addition, the need of calibration of a Li QRE in Mg‐ion‐based systems and its effect on the cycling performance is pointed out. In order to elucidate the influence of the ES electrolyte additive, pre‐cycled electrodes are studied and the influence of pre‐cycling and ES, especially on the AC, is discussed.

Even though using AC as CE enables hybrid dual‐ion full cells,^[12k]^ in this study, the focus is set on the graphite cathode. We thereby try to pave the way for possible future DIB applications with new Mg^2+^‐storing anode materials[^12i^, ^12j^, ^32^] or even Mg‐metal anodes if suitable protective measures such as an (artificial) Mg^2+^ conductive surface film^[33]^ on the Mg to enable Mg electrodeposition and ‐dissolution with the herein studied electrolytes are found.

## Results and Discussion

As shown recently, the reversible intercalation of TFSI^−^ into/from graphite is possible from a Mg‐ion‐based electrolyte.[^12i^, ^12j^, ^12k^] However, herein, deeper knowledge of graphite as anion‐intercalation cathode for Mg‐ion‐based DIBs is focused and the differences between Li‐ and Mg‐ion‐based DIBs are systematically evaluated. In addition, ES as electrolyte additive and highly concentrated carbonate‐based electrolytes are investigated for application in Mg‐ion‐based DIBs (see Figure 1).

### Mg‐ion‐based dual‐ion batteries

For a direct comparison between Mg‐ and Li‐ion‐based DIBs, cells with the same cell setup (i. e., AC as a CE, and Li metal as a RE or QRE) were compared.^[12k]^ To ensure the same molar concentration of TFSI^−^ and exclude influences based on concentration variations, in this study, 0.5 m Mg(TFSI)_2_ and 1 m LiTFSI in Pyr_14_TFSI were used.

A direct comparison of the specific discharge capacities (SDCs) and coulombic efficiencies (C_Eff_) at 100 mA g^−1^ of graphite ‖ AC cells with Mg‐Pyr and Li‐Pyr with cut‐off potentials of 3.4–5.0 V vs. Li|Li^+^ is given in Figure S1a (Supporting Information). The SDCs of the cells with Li‐Pyr are much higher compared to the ones with Mg‐Pyr, especially in the first cycles (1st cycle: 25±5 vs. 4±4 mAh g^−1^). While these differences are diminishing upon cycling (50th cycle: 40±8 vs. 39±4 mAh g^−1^), the differential capacity versus potential plots (Figure S1b) reveal that this variance mainly occurs from a shift of the overpotential for the anion intercalation into graphite. As pointed out by other studies,[^10b^, ^12k^] the limitation of the cut‐off potential is crucial to achieve high SDCs in combination with a high C_Eff_, resulting in severe changes of the specific capacities if the cut‐off potential is changed. This is based on a staging behavior of the TFSI^−^‐intercalation into graphite at different potentials.[^10b^, ^12k^] In order to evaluate if intrinsic differences of the electrolytes or rather potential differences at the Li|Li^+^ RE/QRE lead to the different cycling performances, the Li RE/QRE was calibrated in different electrolytes using ferrocene/ferrocenium (Fc/Fc^+^) as standard redox reference system.^[31]^ The results show that the redox potential of Fc/Fc^+^ (*ϕ*_Fc/Fc+_) is shifted from 3.10±0.01 V vs. Li|Li^+^ for Li‐Pyr to 3.20±0.03 V vs. Li|Li^+^ for Mg‐Pyr (Figure S2a). To truly evaluate differences in the cycling performance of graphite ‖ AC cells with Li‐Pyr and Mg‐Pyr with Li|Li^+^ RE/QREs, a potential correction of 0.1 V is needed. The upper cut‐off potential of Li‐Pyr was therefore decreased to 4.9 V vs. Li|Li^+^, while the upper cut‐off potential of Mg‐Pyr was kept at 5.0 V vs. Li|Li^+^. In addition to this correction, a pre‐cycle at 10 mA g^−1^ was introduced for both systems to achieve an improved cycling performance even for the initial cycles (compare Figure S3).

**Figure 2:**
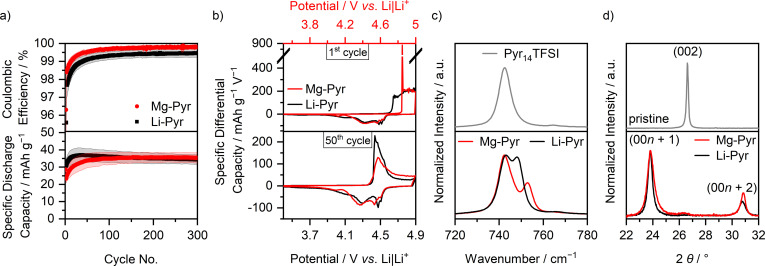
(a) Coulombic efficiency and specific discharge capacity of graphite ‖ AC Swagelok‐type cells (three‐electrode configuration; RE/QRE: Li metal) with 0.5 m Mg(TFSI)_2_ (Mg‐Pyr, red) and 1 m LiTFSI (Li‐Pyr, black) in Pyr_14_TFSI at 100 mA g^−1^ (1st cycle: 10 mA g^−1^) with cut‐off potentials of 3.4 and 4.9 V (Li‐ion‐based) respectively 5.0 V (Mg‐ion‐based) vs. Li|Li^+^ (procedure 1, Table S2a). The coulombic efficiencies of the first cycle (Mg‐Pyr: 74±1 %, Li‐Pyr: 64±2 %) are not shown. (b) Corresponding differential capacity versus potential plots of the 1st and 50th cycle. For an easier comparison, the potential of the Mg‐ion‐based cell (red, upper *x*‐axis) is shifted by 0.1 V compared to the Li‐ion‐based cell (black, lower *x*‐axis), according to variations in the potential of the Li metal QRE. (c) Normalized Raman spectra between 720 and 780 cm^−1^ of Pyr_14_TFSI, and 0.5 m Mg(TFSI)_2_ and 1 m LiTFSI in Pyr_14_TFSI. (d) Normalized ex‐situ XRD patterns of pristine graphite composite electrodes (grey), and electrodes cycled in graphite ‖ AC Swagelok‐type cells (three‐electrode configuration; RE/QRE: Li metal) with 0.5 m Mg(TFSI)_2_ (red) and 1 m LiTFSI (black) in Pyr_14_TFSI after three cycles at 10 mA g^−1^ with cut‐off potentials of 3.4 and 4.9 V (Li‐ion‐based) respectively 5.0 V (Mg‐ion‐based) vs. Li|Li^+^ and one charge to 4.9 V (Li‐ion‐based) respectively 5.0 V (Mg‐ion‐based) vs. Li|Li^+^ (procedure 6, Table S2d, electrodes in charged state).

The SDCs and C_Eff_ values of the corrected comparison of graphite ‖ AC cells with Li‐Pyr and Mg‐Pyr are shown in Figure 2a and Table S3. Even though slight differences in the SDC are visible, especially during the first cycles (1st cycle: Li‐Pyr: 32±1 mAh g^−1^; Mg‐Pyr: 29±1 mAh g^−1^), the performance for later cycles is alike (50th and 300th cycle: Li‐Pyr: 37±4 and 34±3 mAh g^−1^; Mg‐Pyr: 34±4 and 35±3 mAh g^−1^), despite slightly higher C_Eff_ for cells with Mg‐Pyr (1st, 50th, and 300th cycle: Li‐Pyr: 64±2, 99.1±0.3, 99.5±0.2 %; Mg‐Pyr: 74±1, 99.5±0.1, 99.8±0.1 %). These minor differences are quite surprising, since a higher blue shift of coordinated to uncoordinated TFSI^−^ in the Raman spectra (Figure 2c, uncoordinated: 742 cm^−1^, coordinated: 747 and 753 cm^−1^ for Mg‐Pyr and 748 cm^−1^ for Li‐Pyr),^[34]^ as well as theoretical calculations of Li‐TFSI and Mg‐TFSI^[35]^ suggest stronger interactions between Mg^2+^ and TFSI^−^ than for Li^+^ and TFSI^−^. This indicates that either “free” (i. e., not coordinated to a cation) TFSI^−^ is intercalated, or that even with higher ion interactions, breaking the ion coordination is not the rate‐determining step at the positive electrode. As the cathode potential and not the cell voltage is controlled in the cell setup of this study, overpotentials at the anode would likely not be reflected in the capacity or differential capacity versus potential plots, assuming that the AC electrode capacity is highly oversized and therefore not limiting the cell capacity.

The cycling performance of cells with Li‐Pyr is in good agreement with previously reported results of graphite ‖ Li metal cells using LiTFSI in Pyr_14_TFSI as electrolyte, which show SDC of around 40 mAh g^−1^ with a C_Eff_>99 % at a cut‐off potential of 4.9 V vs. Li|Li^+^, considering, that slightly different cycling conditions were used.[^10^, ^17^] Previous studies for DIB cells based on Mg‐Pyr electrolytes showed slightly higher SDCs, that is, 58 mAh g^−1^ [EG ‖ PTCDI (EG=expanded graphite, PTCDI=3,4,9,10‐perylenetetracarboxylic diimide), 200 mA g^−1^ cell voltage range: 1–4 V],^[12i]^ 53 up to 93 mAh g^−1^ (electrolyte with additives, EG ‖ Ti‐Nb_2_O_5_ nanoflakes, 100 mA g^−1^ cell voltage range: 0.5–3.2 V),^[12j]^ and 45 mAh g^−1^ (graphite ‖ AC, 50 mA g^−1^, 3.4–5.0 V vs. Li|Li^+^).^[12k]^ However, a direct comparison is rather difficult as these differences likely occur from the use of different additives,^[12j]^ different graphite materials,[^12i^, ^12j^] as well as different specific currents and cut‐off potentials, respectively potentials of the reference system and voltage‐controlled instead of potential‐controlled cells. A more detailed comparison to the results of Meister et al.^[12k]^ show that the intercalation potential is lower (≈4.4 V vs. Li|Li^+^) compared to the herein reported results (approx. 4.5 V *vs*. Li|Li^+^). This indicates that even for the same electrolyte with different concentrations, potential shifts of a Li|Li^+^ QRE occur. This underlines the importance of a calibration with Fc/Fc^+^ using Li|Li^+^ as QRE. Considering this, the values reported here are in good agreement with battery cells under comparable conditions (37±1 mAh g^−1^ with an upper cut‐off potential of 4.9 V vs. Li|Li^+^).^[12k]^

In order to gain deeper insights into the cycling performances of DIBs, the differential capacity versus potential plots of the 1st and 50th cycle are displayed in Figure 2b. Here, also the main difference between the cycling performances of cells with Li‐Pyr and Mg‐Pyr is visible: the onset potential in the first respective cycle. While TFSI^−^ from Li‐Pyr intercalates at 4.61±0.01 V vs. Li|Li^+^ in the first cycle, the onset potential is lowered by approximately 0.23 V and remains constant at 4.38±0.02 V vs. Li|Li^+^ for later cycles (50th, 100th, and 300th cycle, threshold of 10 mAh g^−1^ V^−1^, Table S4). A similar behavior can be observed for cells with Mg‐Pyr: While the first TFSI^−^ intercalation takes place at 4.82±0.01 V vs. Li|Li^+^, a decrease of around 0.36 V for the following cycles is observed (within 4.46±0.03 V vs. Li|Li^+^ for 50th, 100th, and 300th cycle, threshold of 10 mAh g^−1^ V^−1^, Table S4). The increased TFSI^−^ intercalation potential in the first cycle was described to be based on kinetic hindrance and a poor wetting of the electrodes.[^10b^, ^15a^, ^17^, ^36^] Balabajew et al.^[36]^ attributed the reduced overpotentials in later cycles to an opening of the interlayer gaps by remaining TFSI^−^ and an increased number of defects in the graphite, decreasing the overpotentials for later cycles. However, the kinetic hindrance in the first cycle is much larger for cells with Mg‐Pyr compared to the ones with Li‐Pyr, reflected in a much larger potential gap between the 1st and the 300th cycle (0.36 V versus 0.23 V). One reason for this could be the differences in the solution structure of Mg‐Pyr and Li‐Pyr (Figure 2c). While the stronger interactions in Mg‐Pyr do not seem to have a significant effect on the overpotentials for later cycles, they might indeed have an impact on the initial TFSI^−^ intercalation in the first cycle. Apart from this, also differences in the wetting behavior of these electrolytes could be responsible for the higher kinetic hindrance with Mg‐Pyr in the first cycle. The wetting properties of an electrolyte can be estimated based on the contact angle of a sessile drop on a surface: a small contact angle indicates a good wetting behavior, while a large contact angle reflects a poor one.^[37]^ Based on the rough and inhomogeneous surface of graphite composite electrodes, large value deviations and no clear trend could be observed (both electrolytes show contact angles within 25±5° on graphite composite electrodes after 10–15 s). Using a silicon (Si) wafer instead, a higher contact angle of Mg‐Pyr compared to Li‐Pyr (44±1 versus 39±1° after 10–15 s) is observed. This trend is even more pronounced for longer wetting times (e. g., 38±5° for Mg‐Pyr and 28±4° for Li‐Pyr after 180 s).

Since the surface texture and composition of the Si wafer and graphite are different, this does not necessarily reflect the wetting phenomena on graphite. Still, the differences of the contact angles on the Si wafer indicate that the wetting phenomena of these electrolytes can be different, even though they are likely not the only reason for the higher kinetic hindrance of TFSI^−^ intercalation observed in the first cycle with Mg‐Pyr.

Even for the discharge potentials of the first cycle, and also for the charge potentials in following cycles, the TFSI^−^ intercalation potentials are, considering the calibration of the Li|Li^+^ QRE discussed above, similar in cells with Li‐Pyr and Mg‐Pyr. Both show one main intercalation region at 4.4–4.6 V vs. Li|Li^+^ (Li‐Pyr), respectively 4.5–4.7 V vs. Li|Li^+^ (Mg‐Pyr) during charge, and two main deintercalation regions for discharge at 4.5 and 4.4–3.9 V vs. Li|Li^+^ (Li‐Pyr), respectively 4.6 and 4.5–4.0 V vs. Li|Li^+^ (Mg‐Pyr).

In order to gain deeper insides into the intercalation, ex‐situ XRD measurements of cycled electrodes were performed, which provide information about the staging behavior of TFSI^−^ intercalation into graphite.^[17]^ While pristine electrodes show only one distinct (002) reflection at 26.6° upon charging the electrodes to 5.0 V vs. Li|Li^+^ (Mg‐Pyr), respectively 4.9 V vs. Li|Li^+^ (Li‐Pyr), the (002) reflection vanishes, while the (00*n*+1) and (00*n*+2) reflections arise (Figure 2d). The stage number can be estimated based on the ratio of the two reflections *d*(00*n*+2)/*d*(00*n*+1) and is 1.29 for both electrodes cycled with Mg‐Pyr (at 5.0 V vs. Li|Li^+^) and Li‐Pyr (at 4.9 V vs. Li|Li^+^), indicating dominant stages of 2 to 3.^[17]^ The results of Li‐Pyr fit well with previously reported values for TFSI^−^ intercalation into graphite from 1 m LiTFSI in Pyr_14_TFSI at 20 °C with similar upper cut‐off potentials.^[17]^ Considering the achieved specific charge capacities of TFSI^−^ intercalation into graphite (Table S5: Mg‐Pyr: 42 mAh g^−1^; Li‐Pyr: 44 mAh g^−1^) as well as the dominant stage (between 2 and 3), a maximum intercalation stoichiometry of (TFSI)C_26_ can be estimated, matching previous results at 20 °C.^[17]^ Additional XRD data at various cut‐off potentials are given in Figure S4 and Table S5. Here, a similar performance of Mg‐Pyr and Li‐Pyr is visible, even though lower potentials for discharging are needed for Li‐Pyr, in good agreement with slight differences in the differential capacity versus potential plots (Figure 2b). The diffraction patterns of the electrodes cycled with both electrolytes at 3.4 V vs. Li|Li^+^ before and after the 4th cycle with mainly one reflection at 26.6±0.1° (Figure S4a and b) indicate that no or only very low amounts of TFSI^−^ remain in the graphite at a potential of 3.4 V vs. Li|Li^+^.

Apart from XRD, which gives insights into the bulk structure of graphite, Raman spectroscopy can be used to study anion intercalation into graphite closer to the surface.[^12i^, ^12j^, ^36^, ^38^] The ex‐situ Raman spectrum of a pristine graphite electrode (Figure S5a) shows the D band associated to the *A*_1g_ symmetry at approximately 1352 cm^−1^ and a G band for the *E*_2g_ vibrational mode at 1580 cm^−1^.^[38a]^ In addition, a small shoulder of the G band at 1623 cm^−1^ indicates the D’ band.[^38a^, ^39^] The G band originates from a crystalline sp^2^ network within the graphite, while the D and D’ bands originate from defects in the graphite structure.[^38a^, ^39^] The appearance of the D bands even in pristine graphite indicates defects even before cycling and is in good agreement with defects observed via SEM (Figure S7).

Upon charging, respectively ion intercalation into the graphite, the G band splits into two Raman bands, associated to graphite layers not adjacent to layers with intercalated anions [*E*_2g2_(i)] and layers adjacent to layers with intercalated anions [*E*_2g2_(b)] at higher wavenumbers.[^36^, ^38a^] Based on the ratio of the band intensities, the intercalation stage can be estimated, as described in more detail elsewhere.[^36^, ^38a^] Graphite with stage *n*≤2 only consists of graphite layers adjacent to layers with intercalated anions and therefore does not show the *E*_2g2_(i) band.[^36^, ^38a^]

**Figure 3:**
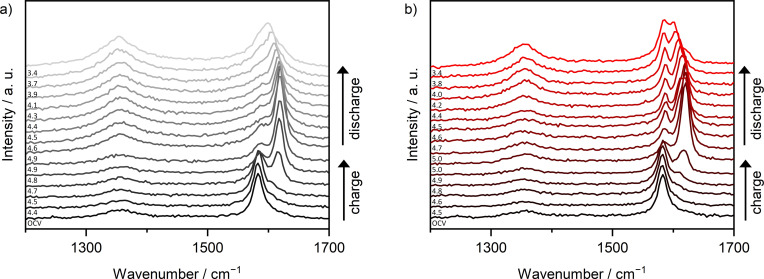
In‐situ Raman spectra at selected potentials of the 2nd cycle of modified graphite ‖ AC pouch‐type cells (three‐electrode configuration; RE/QRE: Li metal) between 1200 and 1700 cm^−1^ with (a) Li‐Pyr (black) and (b) Mg‐Pyr (red) electrolytes at a laser intensity of 1 % (acquisition time: 5×30 s). The corresponding CVs [scan speed: 0.5 mV s^−1^, cut‐off potentials of 3.4–4.9 V (Li‐ion‐based), respectively 5.0 V vs. Li|Li^+^ (Mg‐ion‐based)] are displayed in Figure S6. At the upper potential of 4.9 V, respectively 5.0 V vs. Li|Li^+^, two spectra recorded before and after a constant potential step of 5 min are shown.

In‐situ Raman spectra of the second cycle (cyclic voltammetry, CV) of modified graphite ‖ AC pouch cells with Li‐Pyr and Mg‐Pyr are given in Figure 3. Cells with both electrolytes show a clearly arising *E*_2g2_(b) band at 1618–1620 cm^−1^ upon charging. At the upper cut‐off potential (Li‐ion‐based: 4.9 V vs. Li|Li^+^; Mg‐ion‐based: 5.0 V vs. Li|Li^+^), however, the *E*_2g2_(i) band at around 1583 cm^−1^ is still visible, indicating, that stage 2 is not reached. Since the *E*_2g2_(i) band is quite small, though, a mixture of different stages might be present, in good agreement with the staging observed using XRD (Figure 2d, Table S5). After discharging at 3.4 V vs. Li|Li^+^, besides the *E*_2g2_(i) band at around 1580 cm^−1^, also an overlapping band at higher wavenumbers is visible and indicates remaining TFSI^−^ in the graphite. In addition, the intensity of the D band is much higher in comparison to the band before the second cycle and the pristine graphite electrode, indicating more defects. This is in good agreement with the results from Balabajew et al.^[36]^ Since no significant amounts of TFSI^−^ remaining in the graphite could be observed by using XRD, it is indicated that most of the TFSI^−^ in the bulk (observed by XRD) is removed upon discharging, while some TFSI^−^ close to the surface (detected by Raman spectroscopy) remains in the graphite.[^38d^, ^38e^] An irreversible intercalation of TFSI^−^ is also in good agreement with the much lower C_Eff_ observed in the first cycles for all electrolytes used in this study (e. g., <80 % in the 1st cycle, Table S3).

The first in‐situ Raman spectra showing the *E*_2g2_(b) band upon charging are at potentials of 4.7 V vs. Li|Li^+^ for the cell with Li‐Pyr (Figure 3a) and 4.8–4.9 V vs. Li|Li^+^ for the cell with Mg‐Pyr (Figure 3b). These potentials are higher than the main intercalation peaks observed in the CV (≈4.4–4.5 V vs. Li|Li^+^) but are in good agreement with the higher onset potentials observed in the first cycle (Figure S6). This is likely based on the fact that not all graphite is fully charged within the first cycle, which was also observed by the activation processes during the first cycles. The full capacity of graphite ‖ AC cells is only reached after several cycles (Figure 2a). Also, in the CVs (Figure S6) the second intercalation peak in the second cycle starting at 4.8 V (Li‐ion‐based) respectively 4.9 V (Mg‐ion‐based) overlaps with the peak observed in the first cycle and might reflect TFSI^−^ intercalation into “pristine” graphite. The phenomena observed might therefore rather be first cycle effects and explain why the Raman spectra before the second cycle (open‐circuit potential “OCP”) do not show the *E*_2g2_(b) band or increased intensity of the D band, as observed after the second discharge.

The effect of long‐term cycling on the electrode was additionally studied by ex‐situ Raman spectroscopy measurements of graphite electrodes after 100 cycles (Figure S7). The electrodes were not washed prior to the Raman measurements to avoid alterations of the electrode structure. In the discharged state (solid line), beside the *E*_2g2_(i) mode band at around 1583 cm^−1^, for the electrodes cycled in both electrolytes also an additional band is visible at around 1605 cm^−1^, likely indicating remaining TFSI^−^ in the graphite respectively the *E*_2g2_(b) mode. This is in good agreement with the in‐situ Raman spectroscopy measurements and the results of Balabajew et al.^[36]^ Still, even after 100 cycles, for both electrodes the intensity of the *E*_2g2_(i) mode band is higher compared to the one of the *E*_2g2_(b) mode. In addition, the two Raman bands are separated. This is different from the in‐situ Raman spectra, which showed a strong overlapping of the bands and similar intensities. Furthermore, the intensity of the D band at around 1352 cm^−1^ is lower in comparison to the one observed in the in‐situ Raman spectra. A similar trend of a more pronounced band separation as well as a decreasing intensity of the D band was observed by Balabajew et al.^[36]^ after rest periods in in‐situ Raman cells, as well. They assigned this effect to TFSI^−^ ordering by lateral diffusion. The comparably short rest period (at least several hours) between the end of the cycling investigation and the ex‐situ Raman measurement might have already been enough to induce TFSI^−^ ordering. These observations underline the importance of in‐situ experiments. Even though the Raman spectra indicate remaining TFSI^−^ in the graphite in the discharged state, no clear alteration of the electrode morphology, such as graphite exfoliation^[40]^ can be observed via SEM of the washed electrodes after 100 cycles (Figure S7).

In the Raman spectra of the charged electrodes, the Raman bands of both modes shift to higher wavenumbers [*E*_2g2_(i)≈1590 cm^−1^; *E*_2g2_(b)≈1614 cm^−1^]. In addition, the ratios of the two modes change drastically with the *E*_2g2_(i) mode band being the dominant one. Still, the remaining band at around 1590 cm^−1^ indicates a stage below 2, in good agreement with the in‐situ Raman spectra at 4.9 V (Li‐ion‐based), respectively 5.0 V vs. Li|Li^+^ (Mg‐ion‐based) of the second cycle and the estimated stage based on XRD (between stage 2 and 3). Similar to the discharged state, SEM images of the charged electrodes show no clear alteration of the electrode morphology (Figure S7).

**Figure 4:**
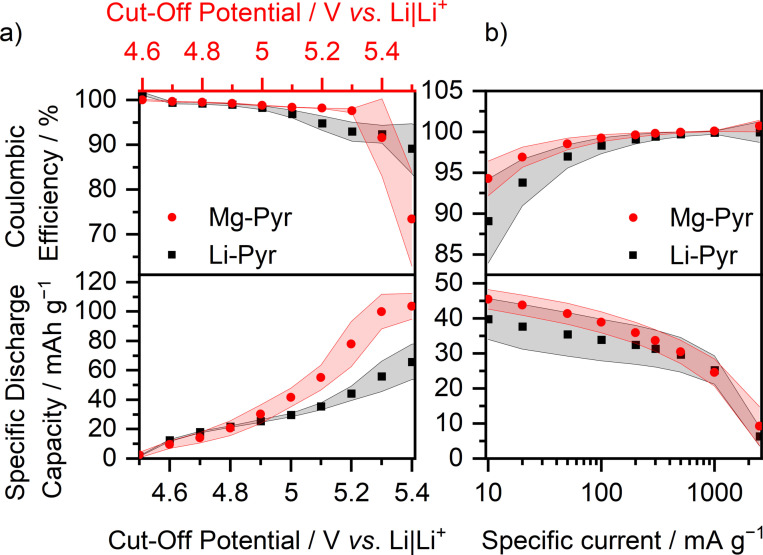
Coulombic efficiency (upper part) and specific discharge capacity (lower part) of graphite ‖ AC Swagelok‐type cells (three‐electrode configuration; RE/QRE: Li metal) with 0.5 m Mg(TFSI)_2_ (Mg‐Pyr, red) and 1 m LiTFSI (Li‐Pyr, black) in Pyr_14_TFSI (a) at different cut‐off potentials at a specific current of 100 mA g^−1^ (procedure 4, Table S2b, the values of the 10th cycle of each potential are displayed) and (b) at different specific currents with cut‐off potentials of 3.4 and 4.9 V (Li‐ion‐based) respectively 5.0 V (Mg‐ion‐based) vs. Li|Li^+^ (procedure 5, Table S2c, the values of the 5th cycle of each current are displayed). For an easier comparison in (a), the potential of the Mg‐ion‐based cell (red, upper *x*‐axis) is shifted 0.1 V compared to the Li‐ion‐based cell (black, lower *x*‐axis), according to variations in the potential of the Li metal RE/QRE.

The limits of Mg‐ion‐based DIBs are evaluated based on their ability to perform at higher cut‐off potentials, as well as higher specific currents. In good agreement with previously reported results for different DIBs,[^12k^, ^17^] the SDC of TFSI^−^ intercalation into graphite increases with higher cut‐off potentials for cells with both, Mg‐Pyr and Li‐Pyr (Figure 4a, Table S6). This increase is more distinct for cells with Mg‐Pyr reaching maximum values of 104±9 mAh g^−1^, while a maximum SDC of 66±12 mAh g^−1^ is reached in cells with Li‐Pyr at 5.5 and 5.4 V vs. Li|Li^+^. The increased SDCs, however, are accompanied by a decrease of the C_Eff_, especially for Mg‐Pyr‐containing cells. The C_Eff_ is decreased from values above 99 % for 4.9 V (respectively 4.8 V for Li‐Pyr) vs. Li|Li^+^ to 73±11 % (89±6 %) for 5.5 V (5.4 V for Li‐Pyr) vs. Li|Li^+^. Based on the drastic decrease of the C_Eff_ for higher cut‐off potentials, it is assumed, that the maximum applicable cut‐off potential for graphite ‖ AC cells with Mg‐Pyr is 5.3 V vs. Li|Li^+^. The increased capacity is based on additional TFSI^−^ intercalation, respectively on higher staging numbers of graphite at higher potentials, which can also be observed as additional peaks in the differential capacity versus potential plots with various upper cut‐off potentials (Figure S8a).^[17]^ In addition to an extra peak at approx. 5.2 V vs. Li|Li^+^, a peak at approximately 4.8 V vs. Li|Li^+^ rises, when cycled to higher potentials, which might be based on diminished overpotentials^[41]^ also for lower staging (e. g., caused by irreversible widening of the graphite structure) once high stages were reached. Long‐term cycling of graphite ‖ AC cells with Mg‐Pyr and upper cut‐off potentials of 5.3 V vs. Li|Li^+^ (Figure S8c) shows, that, despite the high SDCs in the first cycles, the capacity fades during cycling and decreases from 86±5 mAh g^−1^ (C_Eff_: 97.2±0.9 %) in the 50th cycle to only 55±11 mAh g^−1^ (C_Eff_: 97.7±0.5 %) in the 300th cycle. This is based on higher overpotentials, as well as a lower SDC per intercalation, visible in the differential capacity versus potential plots of the 50th and 300th cycle (Figure S8b) and might result from electrolyte (e. g., intercalated TFSI^−^) or electrode decomposition, also reflected in the lower C_Eff_ compared to the values at a cut‐off potential of 5.0 V vs. Li|Li^+^. Variations of the specific current (Figure 4b, Table S7) show that the SDC of graphite ‖ AC cells with Mg‐Pyr and Li‐Pyr is slightly increased at lower currents, but also that the C_Eff_ is lowered simultaneously (Mg‐Pyr: 10 mA g^−1^: 46±3 mAh g^−1^, 94±2 %; 100 mA g^−1^: 39±3 mAh g^−1^, 99.2±0.4 %; Li‐Pyr: 10 mA g^−1^: 40±6 mAh g^−1^, 89±5 %; 100 mA g^−1^: 34±6 mAh g^−1^, 98±1 %), in good agreement with previously reported results in Li‐ion‐based DIBs.^[15a]^ The reduced C_Eff_ might originate from enhanced parasitic side reactions due to electrolyte decomposition, in particular decomposition of the intercalated TFSI^−^ anions. On the other side, high specific currents increase the C_Eff_, indicating that parasitic side reactions are suppressed, but significantly decrease the SDC for currents above 1000 mA g^−1^ (2500 mA g^−1^: Mg‐Pyr: 9±6 mAh g^−1^, 100.7±0.7 %; Li‐Pyr: 6±3 mAh g^−1^, 100±1 %). Still, from 10–500 mA g^−1^, both the specific capacity (≈30–45 mAh g^−1^) and the C_Eff_ (>90 %) are adequate.

Also, elevated operating temperatures can significantly change the intercalation behavior of TFSI^−^ into graphite, as shown for Li‐ion‐based electrolytes.[^17^, ^41^] A similar behavior can also be observed in the Mg‐ion‐based system (Figure S9, Table S3). Especially in the first cycle, the onset potential for TFSI^−^ intercalation is diminished drastically (Table S4, 20 °C: 4.82±0.01 V vs. Li|Li^+^; 60 °C: 4.54±0.02 V vs. Li|Li^+^), even though the onset potentials are similar for later cycles (50th cycle at 20 °C: 4.48±0.01 V vs. Li|Li^+^; at 60 °C: 4.43±0.03 V vs. Li|Li^+^). In addition, the SDCs in the first cycles are with 52±1 (1st cycle) to 56±2 mAh g^−1^ (50th cycle) almost twice as high as the values at 20 °C, even though the C_Eff_ is reduced drastically (50th cycle: 95.0±0.9 %). This could be based on improved kinetics for TFSI^−^ intercalation, visible in additional intercalation peaks, respectively significantly lower overpotentials for TFSI^−^ intercalation,^[41]^ at 4.7 and 4.9 V vs. Li|Li^+^ and deintercalation at 4.9 and 4.7 V vs. Li|Li^+^, as well as a broader peak from 4.5–4.1 V vs. Li|Li^+^ (Figure S9b). Still, upon long‐term cycling the capacity is fading, similar to the performance at higher cut‐off potentials, probably based on electrolyte (e. g., intercalated TFSI^−^) and/or electrode degradation (Figure S9a).

Overall, and in agreement with previous studies,[^12i^, ^12j^, ^12k^] these results indicate that TFSI^−^ intercalation into graphite is possible from a Mg‐ion‐based electrolyte and has a similar performance compared to a Li‐ion‐based system. The achievable SDCs, however, are still lower compared to other cathode materials such as layered sulfides and oxides (often >100 mAh g^−1^) or sulfur (up to 1000 mAh g^−1^).[^5c^, ^6d^, ^7^, ^8b^, ^8c^, ^8d^] In addition, in DIBs the electrolyte is part of the active material, which is why typically larger amounts of electrolyte are needed in comparison to cells based on the “ion transfer” mechanism, where the electrolyte is only a charge carrier between the electrodes. This also increases the share of inactive electrolyte components and thus the achievable energy density in DIBs.^[15g]^ High cut‐off potentials and elevated temperatures are able to significantly increase the SDCs, but at the cost of the C_Eff_ and long‐term cycling stability. In the following, further approaches to increase the SDCs using additives and alternative concepts based on highly concentrated electrolytes will be discussed.

### Electrolyte additives for Mg‐ion‐based dual‐ion batteries

The utilization of electrolyte additives was shown to improve the overall cycling performance in DIBs with several electrolyte formulations based on various cations including Li^+^, Na^+^, and K^+^.[^12a^, ^12b^, ^14^] Also for Mg‐ion‐based DIBs, an ionic liquid‐based electrolyte mixed with organic solvent additives (a mixture of cyclic and linear carbonates, as well as glymes) showed almost twice the capacity (up to 93 mAh g^−1^) compared to additive‐free cell setups.^[12j]^ Yang et al.^[12j]^ showed an excellent rate capability and C_Eff_ for Mg‐ion‐based DIB cells at high specific currents, even though the C_Eff_ decreased drastically to below 90 % at a low specific current (100 mA g^−1^). Also, they used a quite high total additive amount of 10 wt%. For several alkali‐metal‐based DIBs the addition of even small amounts (2 wt%) of ES significantly increased the achieved capacity (e. g., the capacity increased from 50 to ≈97 mAh g^−1^ for 1 m LiTFSI in Pyr_14_TFSI in graphite ‖ Li metal DIB cells^[14]^).[^12a^, ^12b^]

**Figure 5:**
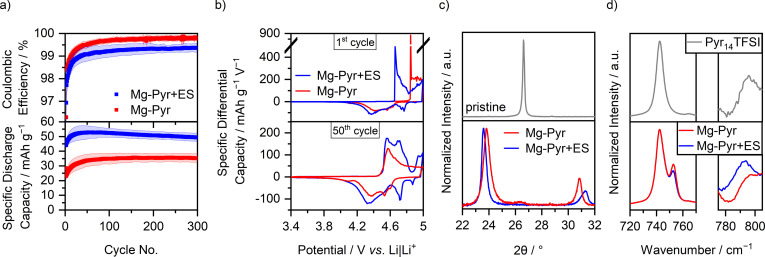
(a) Coulombic efficiency and specific discharge capacity of graphite ‖ AC Swagelok‐type cells (three‐electrode configuration; QRE: Li metal) with 0.5 m Mg(TFSI)_2_ (Mg‐Pyr, red) and 0.5 m Mg(TFSI)_2_+2 wt% ES (Mg‐Pyr+ES, blue) in Pyr_14_TFSI at 100 mA g^−1^ (1st cycle: 10 mA g^−1^) with cut‐off potentials of 3.4 and 5.0 V vs. Li|Li^+^ (procedure 1, Table S2a). The Coulombic efficiencies of the first cycle (Mg‐Pyr: 74±1 %, Mg‐Pyr+ES: 73±2 %) are not shown. (b) Corresponding differential capacity versus potential plots of the 1st and 50th cycle. (c) Normalized ex‐situ XRD patterns of pristine graphite composite electrodes (grey), and electrodes cycled in graphite ‖ AC Swagelok‐type cells (three‐electrode configuration; RE/QRE: Li metal) with 0.5 m Mg(TFSI)_2_ (red) and 0.5 m Mg(TFSI)_2_+2 wt% ES (blue) in Pyr_14_TFSI after three cycles at 10 mA g^−1^ with cut‐off potentials of 3.4 and 5.0 V vs. Li|Li^+^ and one charge to 5.0 V vs. Li|Li^+^ (procedure 6, Table S2d, electrodes in charged state). (d) Normalized Raman spectra of Pyr_14_TFSI, and 0.5 m Mg(TFSI)_2_ (red) and 0.5 m Mg(TFSI)_2_+2 wt% ES (blue) in Pyr_14_TFSI between 720 and 770 cm^−1^ and 775 and 805 cm^−1^. The different regions are normalized separately.

Similar to alkali‐metal‐based DIB systems, the addition of only 2 wt% of ES to Mg‐Pyr (Mg‐Pyr+ES) enhances the SDC of graphite ‖ AC cells drastically (Figure 5a, Table S3). The SDC of the 50th cycle is increased from 34±4 mAh g^−1^ to 53±4 mAh g^−1^, even though the capacity slightly fades for Mg‐Pyr+ES (300th cycle: 50±3 mAh g^−1^), while it remains constant for Mg‐Pyr (300th cycle: 35±3 mAh g^−1^). These higher capacities can be correlated to additional intercalation peaks at around 4.7 and above 4.9 V vs. Li|Li^+^ (Figure 5b), similar to the ones observed at elevated temperatures (Figure S9), assuming that the addition of ES reduces the overpotentials for TFSI^−^ intercalation, for example, by reducing the interfacial limitations. It can be excluded that a reversible redox reaction of the ES itself is responsible for this improved SDCs, since no significant SDCs could be observed using an inert platinum (Pt) electrode (Pt ‖ AC cells with Mg‐Pyr+ES show <1 μAh discharge capacity even at an upper cut‐off potential of 5.4 V vs. Li|Li^+^). Also, the ex‐situ XRD patterns reveal (Figure 5c, Table S5) that the graphite cathode cycled to 5.0 V vs. Li|Li^+^ with Mg‐Pyr+ES shows a slightly higher staging (stage 2) compared to the one cycled in Mg‐Pyr (stage 2 to 3), indicating that the process of reversible TFSI^−^ intercalation itself and not side reactions are responsible for the increased capacity. In addition, the onset potential for the intercalation of TFSI^−^ in the first cycle is significantly lower upon the addition of ES (4.82±0.01 versus 4.64±0.02 V vs. Li|Li^+^, Table S4), even though it is alike for later cycles (within 4.44±0.05 V vs. Li|Li^+^). This severe difference indicates that the increased kinetic hindrance, which could be observed for Mg‐Pyr in comparison to Li‐Pyr (Figure 2b), can be decreased through the addition of ES, again similar to the results observed at elevated temperatures.

**Figure 6:**
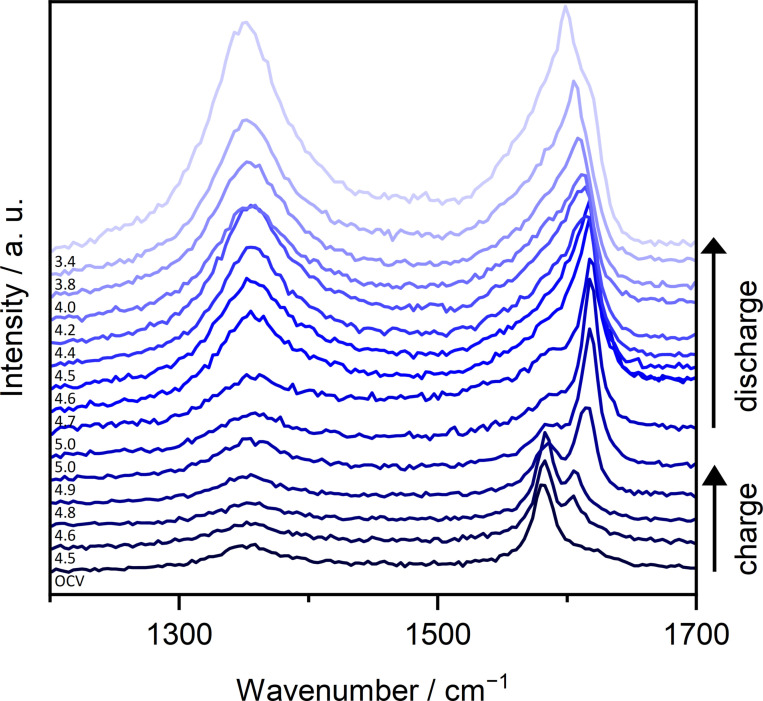
In‐situ Raman spectra at selected potentials of the 2nd cycle of modified graphite ‖ AC pouch‐type cells (three‐electrode configuration; RE/QRE: Li metal) between 1200 and 1700 cm^−1^ with Mg‐Pyr+ES at a laser intensity of 1 % (acquisition time: 5×30 s). The corresponding CVs (scan speed: 0.5 mV s^−1^, cut‐off potentials of 3.4–5.0 V vs. Li|Li^+^) are displayed in Figure S12. At the upper potential of 5.0 V vs. Li|Li^+^, two spectra recorded before and after a constant potential step of 5 min are shown.

In‐situ Raman spectra of the second cycle of a modified graphite ‖ AC pouch‐type cell with Mg‐Pyr+ES are given in Figure 6. Different from the cell cycled with Mg‐Pyr (Figure 3b), the *E*_2g2_(b) band at around 1605 cm^−1^ starts to arise at potentials of 4.5 V vs. Li|Li^+^ in good agreement with the reduced onset potential observed in cells with Mg‐Pyr+ES especially for the first cycle (Table S4). Upon cycling, the *E*_2g2_(i) band decreases in intensity, while the *E*_2g2_(b) band increases and shifts to around 1618 cm^−1^. At 5.0 V vs. Li|Li^+^, the *E*_2g2_(i) band is still visible as a small shoulder of the *E*_2g2_(b) band. Even though this indicates the presence of stages>2, the lower intensity of the *E*_2g2_(i) band in comparison to the one observed with Mg‐Pyr (Figure 3b) is in good agreement with the higher staging observed via XRD (Figure 5c). Apart from differences between the bulk and surface intercalation, the lower staging observed via Raman spectroscopy might also arise from the different cycling procedures used for XRD and in‐situ Raman spectroscopy investigations. Upon discharging, the *E*_2g2_(b) band shifts to lower wavenumbers, while the D band increases drastically in intensity. At 3.4 V vs. Li|Li^+^, the high intensity of the D band as well as the broad G band indicate a high number of defects as well as remaining TFSI^−^ in the graphite, similar to the results obtained without the addition of ES (Figure 3b). Since no splitting of the (002) reflection was observed at 3.4 V vs. Li|Li^+^ in a graphite ‖ AC Swagelok‐type cell (Table S5), this effect is likely mainly occurring at the surface of graphite.

The ex‐situ Raman spectra of graphite composite electrolytes after long‐term cycling with Mg‐Pyr+ES (Figure S7, blue) are similar to the ones obtained after cycling with Mg‐Pyr (Figure S7, red). In the discharged state, the occurrence of the *E*_2g2_(b) band at 1608 cm^−1^ beside the *E*_2g2_(i) band at 1583 cm^−1^ indicates remaining TFSI^−^ in the graphite. The *E*_2g2_(i) band observed in the Raman spectrum of the charged electrode, instead, indicates that stages≥2 are formed. Neither the band intensities nor band positions of the electrodes cycled with Mg‐Pyr and Mg‐Pyr+ES differ significantly and therefore do not explain the improved cycling performance upon the addition of ES. The morphology of the washed electrodes observed via SEM shows no sign of exfoliation or other aging processes, in both charged or discharged state (Figure S7).

Contact angle measurements show that the addition of 2 wt% significantly improved the wetting behavior of Mg‐Pyr on a Si wafer (Mg‐Pyr: 44±1°; Mg‐Pyr+ES: 36±4° after 10–15 s, Mg‐Pyr: 38±5°; Mg‐Pyr+ES: 26±7° after 180 s), even though no clear trend could be observed on graphite (within 25±5° after 10–15 s). This finding might indicate an improved wetting performance on graphite and can, among others, be one reason for the improved onset potential in the first cycle.

Raman spectroscopy of the electrolytes was performed to evaluate possible changes of the solution structure, such as a different coordination of anion and cation occurring upon the addition of ES. The ion coordination of TFSI^−^ can be observed at the Raman band at approximately 740–760 cm^−1^, associated to the expansion and contraction of the TFSI^−^‐anion. In solution, cation and anion can either be completely separated (solvent‐separated ion pairs, SSIPs), form contact ion pairs, (CIPs) with one, or aggregates (AGGs) with several cations in one complex. While “free” TFSI^−^ (SSIP) is located at approximately 742 cm^−1^, the band is shifted to higher wavenumbers [up to 756 cm^−1^ for Mg(TFSI)_2_], if coordinated to cations.[^34^, ^42^] The spectrum of Mg‐Pyr (Figure 5d) shows three bands at 742±1 (“free” TFSI^−^), 747±1, and 753±1 cm^−1^ (coordinated TFSI^−^), in good agreement with previous reports.^[34]^ Giffin et al.^[34a]^ assigned the mid‐frequency band to bridging AGG TFSI^−^ as well as mono‐dentate and weakly bound CIP TFSI^−^, while the band at higher frequencies was attributed to a bidentate coordinated TFSI^−^ in CIPs. The much higher blue shift of the bidentate coordinated TFSI^−^ reflects a stronger binding energy.^[34a]^ Upon the addition of ES, the band positions do not change, and also the amount of “free” TFSI^−^ remains constant (69±1 to 71±1 %), but a decrease of the high‐frequency band is visible (24±1 to 19±1 %, Figure S10a, Table S8). This indicates that coordination of Mg^2+^ by TFSI^−^ is partially exchanged by ES. This trend is more pronounced (decrease to 15±1 %), if the amount of ES is increased to approximately 10 wt%, equaling three ES molecules per Mg^2+^ (Mg‐Pyr+10ES, Figure S10b and Table S8). However, since both, TFSI^−^ and ES contain sulfoxide‐groups and show many overlapping Raman bands also in the band region at 720–780 cm^−1^ (Figure S10b), and the amount of ES is very low, these findings might not reflect the true solution structure. In addition, however, an additional Raman band at 789±1 cm^−1^, which can most likely be attributed to ES coordinated to Mg^2+^, arises [compare 0.5 m Mg(TFSI)_2_ in ES and ES, Figure 5d and S7]. This is especially pronounced for Mg‐Pyr+10ES, indicating that at least some of the ES molecules are coordinated to Mg^2+^. The addition of EC to Mg‐Pyr results in a similar effect on the solution structure (Table S8 and Figure S11), but the cycling performance is, except for the onset potential of the first cycle, not improved significantly compared to the additive‐free electrolyte, indicating that neither the solution structure nor processes in the electrolyte are solely causing the enhanced SDC, but also (side‐)reactions at the electrodes. Even though the amount of ES is quite small, during cycling the ES or its decomposition products might accumulate on the electrode surfaces and therefore enhance the kinetics only locally. For example, the addition of ES to a carbonate‐based electrolyte in LIB cells was found to form sulfite species,^[43]^ such as ROSO_2_Li and Li_2_SO_3_, as well as alkyl sulfide species^[43d]^ on the graphitic anode. The reaction mechanisms in ionic liquid‐ and carbonate‐based systems, however, might differ significantly. In ionic liquid‐based systems, especially with TFSI^−^ anions, the products of the ES are to the best of our knowledge not well investigated yet. In addition, the working potential of the AC anode used in this study is with approximately 2.5–3.5 V vs. Li|Li^+^ much higher in comparison to graphite Li^+^ intercalation anodes. The large amounts of sulfur species of the TFSI^−^ anion and its decomposition products, however, hamper the detection of sulfur species, which might be responsible for the improved performance.

In order to elucidate if ES forms a (permanent) cathode electrolyte interphase (CEI^[44]^), or if short‐term effects are responsible for the improved capacity, respectively lower overpotential, pre‐cycled graphite cathodes (three cycles at 10 mA g^−1^ from 3.4 to 5.0 V vs. Li|Li^+^ in graphite ‖ AC Swagelok‐type cells with Mg‐Pyr+ES) were cycled with an ES‐free electrolyte (graphite ‖ AC Swagelok‐type cells with Mg‐Pyr) afterwards (Figure S13, Table S9). The three pre‐cycles in cells with Mg‐Pyr+ES (grey/black) show higher SDCs compared to the pre‐cycle in cells with Mg‐Pyr (red, Figure S13a, magnification). Also, the differential capacity versus potential profile (Figure S13c, cyan) is typical for a Mg‐Pyr+ES containing cell (blue) and shows additional peaks at 4.7 and 4.9–5.0 V vs. Li|Li^+^ (charge) and 4.7 V vs. Li|Li^+^ (discharge) compared to cells with Mg‐Pyr (red). When exchanging the electrolyte with Mg‐Pyr and using a pristine AC‐based CE after three pre‐cycles in Mg‐Pyr+ES (Figure S13a), the SDC decays and reaches a similar SDC (150th cycle: 39±3 mAh g^−1^, 300th cycle: 38±3 mAh g^−1^) as cells without pre‐cycling in Mg‐Pyr+ES (150th cycle: 36±3 mAh g^−1^, 300th cycle: 35±3 mAh g^−1^, Table S3) after ca. 150 cycles. The SDC of a cell with Mg‐Pyr+ES instead is much higher (150th cycle: 52±3 mAh g^−1^, 300th cycle: 50±3 mAh g^−1^). In addition, even the first cycle with Mg‐Pyr after three pre‐cycles in Mg‐Pyr+ES (Figure S13c, grey) shows a differential capacity versus potential profile typical for Mg‐Pyr (red), without the additional intercalation peaks at 4.7 and 4.9–5.0 V (charge) and 4.7 V vs. Li|Li^+^ (discharge), typical for cells with Mg‐Pyr+ES (Figure S13c, blue and cyan). This indicates that the formation of a (permanent) CEI having a significant impact on the intercalation/de‐intercalation behavior is unlikely. When instead of only graphite also the AC‐based CE is kept when exchanging the electrolyte with Mg‐Pyr after the three pre‐cycles in Mg‐Pyr+ES, the behavior changes drastically (Figure S13, black). Different to the cells with only pre‐cycled graphite (Figure S13a, grey), the cycling performance of a graphite ‖ AC cell with Mg‐Pyr and both pre‐cycled electrodes (Figure S13b, black) is similar to the cycling performance of graphite ‖ AC cells with only Mg‐Pyr+ES (Figure S13b, blue). The SDC increases until a maximum of 52±4 mAh g^−1^ is reached after around 30 cycles and decreases only slightly to 49±3 mAh g^−1^ in the 300th cycle, comparable to cells with Mg‐Pyr+ES (50th cycle: 53±4 mAh g^−1^, 300th cycle: 50±3 mAh g^−1^). In addition, the differential capacity versus potential plots of graphite ‖ AC cells with Mg‐Pyr and both pre‐cycled electrodes show additional peaks at 4.7 and 4.9–5.0 V vs. Li|Li^+^ (charge) and 4.7 V vs. Li|Li^+^ (discharge), which could also be observed in cells with Mg‐Pyr+ES (Figure S13c, black and blue). This effect could be based on a larger amount of Mg‐Pyr+ES kept in the cell when changing the electrolyte (the electrodes where not washed prior to re‐assembly of the cells with fresh Mg‐Pyr electrolyte). However, it is unlikely since (i) the small amount of remaining Mg‐Pyr+ES only on the graphite did not show this effect, even in the first cycles and (ii) the capacity decay of the cell using Mg‐Pyr but pre‐cycled electrodes is not greater than for cells with Mg‐Pyr+ES, indicating that the additive ES is not noteworthy consumed during cycling. Instead, this behavior rather indicates that processes at the AC‐based CE are responsible also for overpotentials at the graphite WE. Apart from a possible surface film formed on the AC CE, this might be based on differences of the solution structure. As discussed above (Figure 5d), Mg^2+^ forms CIPs or aggregates in Pyr_14_TFSI without ES, showing high binding energies.^[34a]^ Processes at the AC CE might therefore rather include the Pyr_14_^+^ cation instead of Mg^2+^ or Mg^2+^ complexes. The Pyr_14_^+^ ion, however, is with a size of 1.1 nm quite large and might not be able to access micropores below 1 nm.^[45]^ In the altered solution structure with ES instead, the desolvation barrier of Mg^2+^ ions coordinated by ES might be lower, allowing for a contribution of the Mg^2+^ ions in the processes at the AC CE and accessibility of micropores below 1 nm and, hence, reduce the resistance and improve the capacity.

Another explanation for this behavior could be an imbalance of side reactions on the WE and CE, explained by the electron inventory effect, which is intrinsic for the dual‐ion storage mechanism.^[46]^ For each electron which is consumed, another electron has to be “generated”, for example, on the other electrode. In addition, and especially relevant for DIBs, the ion couple inventory model states that consumption of an electroactive ion needs to be balanced to ensure ion couples in the electrolyte.^[46]^ More pronounced parasitic reactions on one electrode can therefore lead to irreversibly trapped ions in/on the other electrode and reduce its capacity.^[46]^ In two‐electrode setups, this effect is reflected by fast capacity fading based on anion accumulation in the positive electrode, if side reactions on the negative electrode are more pronounced, or, for example, by Li plating on a negative graphite electrode, if side reactions on the positive electrode predominate. In three‐electrode setups with potential control of the positive WE, instead, mainly effects on the negative electrodes were shown.^[46]^ While side reactions on the AC‐based CE could be balanced by side reactions on the graphite in standard cells (the C_Eff_ of the pre‐cycle is below 80 % in all electrolytes, Table S3), the much higher C_Eff_ of the pre‐cycled graphite (>90 %, Table S9) might not be able to counterbalance for side reactions on the pristine AC electrode, reducing the overall cycling performance. Still, no clear and reproducible differences of the potential of the AC electrodes can be observed, which could support this assumption. Further systematic studies are therefore needed to elucidate this effect in detail. Especially the influence of ES on the potential of the AC also in cell‐voltage controlled setups should be examined.

**Figure 7:**
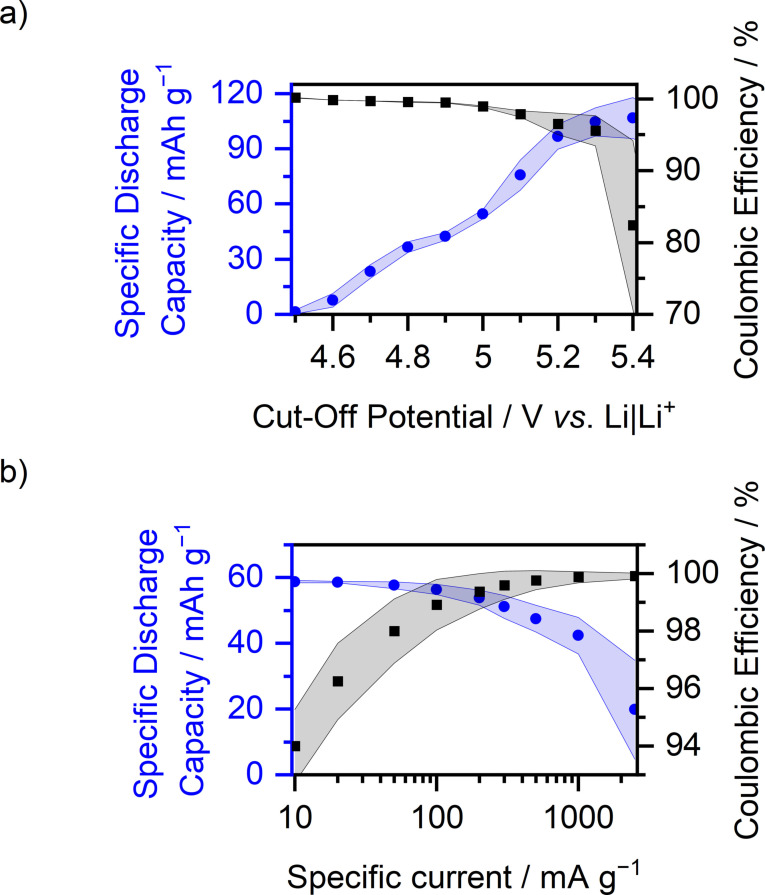
Coulombic efficiency (black) and specific discharge capacity (blue) of graphite ‖ AC Swagelok‐type cells (three‐electrode configuration; QRE: Li metal) with 0.5 m Mg(TFSI)_2_ + 2 wt% ES in Pyr_14_TFSI versus (a) different upper cut‐off potentials at a specific current of 100 mA g^−1^ (lower cut‐off at 3.4 V vs. Li|Li^+^, procedure 4, Table S2b, the values of the 10th cycle of each potential are displayed) and (b) different specific currents with cut‐off potentials of 3.4 and 5.0 V vs. Li|Li^+^ (procedure 5, Table S2c, the values of the 5th cycle of each current are displayed).

Similar to cells with Mg‐Pyr (Figure 4a, Table S6), graphite ‖ AC cells with Mg‐Pyr+ES show an increased SDC with higher cut‐off potentials (Figures 7a and S14a, Table S6). Still, for cells with Mg‐Pyr+ES, the maximum cut‐off potential is limited to 5.3 V vs. Li|Li^+^ (105±8 mAh g^−1^, CE: 96±2 %), based on a drastic decrease of the C_Eff_ for higher cut‐off potentials (5.4 V vs. Li|Li^+^: 82±12 %; 5.5 V vs. Li|Li^+^: 55±10 %). Similar to the results at 5.0 V vs. Li|Li^+^, the achieved SDC at 5.3 V vs. Li|Li^+^ in cells containing Mg‐Pyr+ES is with 105±8 mAh g^−1^ much higher compared to the ones achieved with Mg‐Pyr (78±15 mAh g^−1^), which is mainly based on lower overpotentials for TFSI^−^ intercalation (Figure S14a), also observed in Li‐based DIBs.^[14]^ These results also show that, depending on the upper cut‐off potentials, SDCs similar to or even higher than the results shown by Yang et al.^[12j]^ (max. SDC of 93 mAh g^−1^) could be achieved using 2 wt% of ES instead of 10 wt% of carbonates and glymes as electrolyte additive. These differences, however, can also be based on varying cell setups[^46^, ^47^] (e. g., 3‐electrode versus 2‐electrode cell setup). In addition, with 96±2 % for cut‐offs of 5.3 V vs. Li|Li^+^ the C_Eff_ is much higher compared to the C_Eff_ shown by Yang et al.^[12j]^ at 100 mA g^−1^ (C_Eff_<90 %). XRD patterns of graphite charged to 5.3 V vs. Li|Li^+^ in graphite ‖ AC cells with Mg‐Pyr and Mg‐Pyr+ES were obtained to gain more insights into the effect of ES on TFSI^−^ intercalation (Figure S14b, Table S10). Since the C_Eff_ decreased drastically at a current of 10 mA g^−1^ and a cut‐off potential of 5.3 V vs. Li|Li^+^, the cells were charged with 100 instead of 10 mA g^−1^ after three pre‐cycles at 10 mA g^−1^ and cut‐off potentials of 3.4 and 5.0 V vs. Li|Li^+^. Graphite cycled to 5.3 V vs. Li|Li^+^ in both electrolytes show stage 1 as the main intercalation stage, even though also weaker reflections (indicating lower stages) are observed. Taking the mean SDCs (Table S6) of cells with Mg‐Pyr (78±15 mAh g^−1^) and Mg‐Pyr+ES (105±8 mAh g^−1^) into consideration, this would indicate, that with Mg‐Pyr, only (TFSI)C_26_ (theoretical SDC: 86 mAh g^−1^) can be achieved, while using Mg‐Pyr+ES can lead to a (TFSI)C_20_ stoichiometry (theoretical SDC: 112 mAh g^−1^).^[17]^ These differences were also observed using elevated temperatures.^[17]^ The specific charge capacity of the cell cycled with Mg‐Pyr before XRD measurements (Figure S14b), however, was significantly higher (≈120 mAh g^−1^) than the ones observed during the upper cut‐off experiments, why even stage 1 with (TFSI)C_20_ or higher might be present also for the electrode cycled with Mg‐Pyr.

Also, for different specific currents, cells containing Mg‐Pyr+ES (Figure 7b, Table S7) show a similar trend compared to cells with Mg‐Pyr (Figure 4b, Table S7). While the SDC slightly increases for lower specific currents, the C_Eff_ decreases (10 mA g^−1^: 58.8±0.4 mAh g^−1^, 94±1 %; 100 mA g^−1^: 56±2 mAh g^−1^, 98.9±0.9 %). For higher currents, however, the SDC decreases, especially for specific currents above 1000 mA g^−1^ despite an enhanced C_Eff_ (1000 mA g^−1^: 42±6 mAh g^−1^, 99.9±0.2 %; 2500 mA g^−1^: 20±15 mAh g^−1^, 99.9±0.1 %). Even though the SDC at 2500 mA g^−1^ is with 20±15 mAh g^−1^ still higher compared to the one of cells with Mg‐Pyr (9±6 mAh g^−1^), these results show that, even though kinetics seem to be improved upon the addition of ES, no significant impact on the cell performance at high specific currents can be observed. As described above, likely lower overpotentials for higher staging degrees^[41]^ after the addition of ES result in additional (de‐)intercalation peaks at 4.7 and 4.9–5.0 V (charge) and 4.7 V vs. Li|Li^+^ (discharge). The onset potential of the first TFSI^−^ intercalation peak (except for the first cycle), however, does not change (Tables S4 and S11, 4.46±0.02 V *vs*. Li|Li^+^ at 100 mA g^−1^). At higher specific currents, this onset potential increases for both Mg‐Pyr (1000 mA g^−1^: 4.61±0.02 V *vs*. Li|Li^+^; 2500 mA g^−1^: 4.83±0.07 V *vs*. Li|Li^+^) and Mg‐Pyr+ES (1000 mA g^−1^: 4.60±0.04 V *vs*. Li|Li^+^; 2500 mA g^−1^: 4.79±0.07 V *vs*. Li|Li^+^), decreasing the overall achievable capacities (Figure S15, Table S7 and S11). These results show that ES as electrolyte additive has unique properties to improve the SDC of Mg‐ion‐based DIBs but shows similar limitations regarding the maximum cut‐off potential and applied specific current compared to Mg‐Pyr.

**Figure 8:**
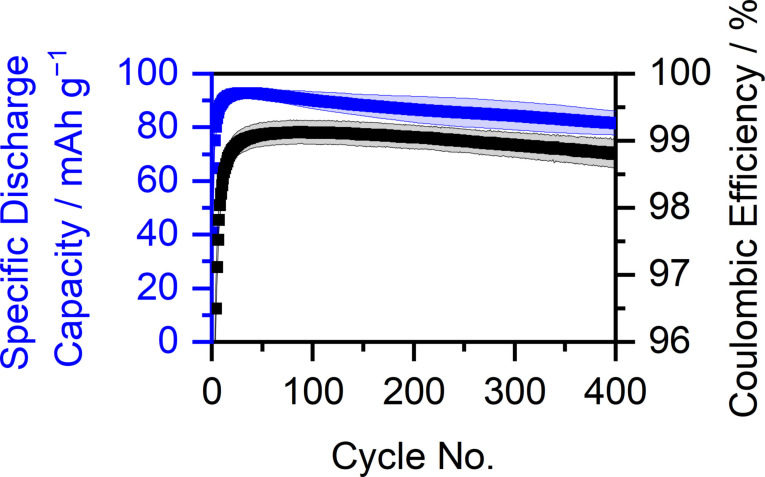
Coulombic efficiency (black) and specific discharge capacity (blue) of graphite ‖ AC Swagelok‐type cells (three‐electrode configuration; QRE: Li metal) with 0.5 m Mg(TFSI)_2_ 2 wt% ES in Pyr_14_TFSI at 300 mA g^−1^ (1st cycle: 10 mA g^−1^) with cut‐off potential of 3.4 and 5.3 V vs. Li|Li^+^ (1st cycle: 5.0 V vs. Li|Li^+^, procedure 3, Table S2a). The Coulombic efficiency of the first cycle (79.2±0.8 %) is not shown.

Combining the good rate capability and improved SDC of graphite ‖ AC cells with Mg‐Pyr+ES, the long‐term performance at 300 mA g^−1^ and an upper cut‐off potential of 5.3 V vs. Li|Li^+^ was evaluated (Figure 8, Table S3). A superior discharge capacity of up to 93±2 mAh g^−1^ in the 30th cycle can be obtained. In addition, the increased specific current improves the C_Eff_ (99.0±0.2 % in the 30th cycle) in comparison to cells cycled at 100 mA g^−1^ (96±2 %, Table S6). Even though both the SDC and C_Eff_ slightly decrease during cycling, an excellent capacity retention of 88 % (of the maximal capacity of the 30th cycle) with a discharge capacity of 82±4 mAh g^−1^ and a CE of 98.8±0.2 % after 400 cycles can be observed (Table S3).

Overall, the addition of only small amounts of ES to Mg‐Pyr can improve the SDC in a graphite ‖ AC cell, without reducing the C_Eff_ or cycling stability significantly. We therefore recommend the addition of small amounts of ES to the electrolyte in Mg‐ion‐based DIBs, if compatible with the anode side. However, more investigations are needed to further understand the impact of ES on the TFSI^−^ intercalation.

### Highly concentrated carbonate‐based electrolytes for Mg‐ion‐based dual‐ion batteries

The implementation of electrolytes with high salt concentrations has been proven beneficial for Li‐ion‐based DIBs, since they offer a high electrochemical stability, little to no Al dissolution, even for TFSI^−^‐based electrolytes, and lower onset potentials for anion intercalation.[^9a^, ^9b^] In addition, high salt concentrations are beneficial in terms of an increased energy density of DIBs.^[15g]^

In order to elucidate if highly concentrated carbonate‐based electrolytes can also be used in Mg‐ion‐based DIBs, Mg(TFSI)_2_ in DMC (dimethyl carbonate) and DEC (diethyl carbonate) with different concentrations were investigated in graphite ‖ AC cells (Figure S16). While the electrolytes with low concentrations (1 m), especially with DMC, show severe parasitic side reactions even below 5.0 V vs. Li|Li^+^, the obtained capacity is lower for high concentrations (3 m). As a compromise between oxidative stability and performance, 2.5 m Mg(TFSI)_2_ in DMC (Mg‐DMC, molar solvent/Mg^2+^ ratio: 4.8 : 1) and 2 m Mg(TFSI)_2_ in DEC (Mg‐DEC, molar solvent/Mg^2+^ ratio: 4.1 : 1) were therefore chosen for further investigations.

**Figure 9:**
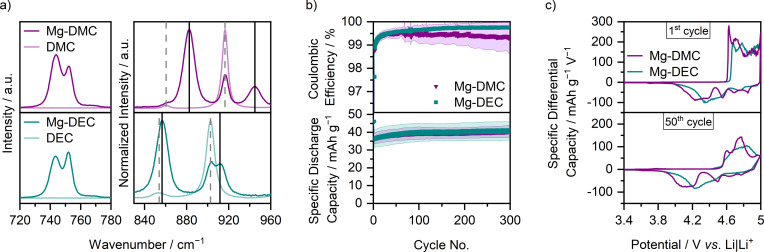
(a) Raman spectra of 2.5 m Mg(TFSI)_2_ in DMC (Mg‐DMC, purple), 2 m Mg(TFSI)_2_ in DEC (Mg‐DEC, cyan), and pure DMC (light purple) and DEC (light cyan) as references between 720 and 780 cm^−1^ and 830 and 960 cm^−1^. The bands for free, respectively coordinated solvent molecules are indicated with dashed grey, respectively solid black lines. (b) Coulombic efficiency and specific discharge capacity of a graphite ‖ AC Swagelok‐type cells (three‐electrode configuration; QRE: Li metal) with 2.5 m Mg(TFSI)_2_ in DMC (purple) and 2 m Mg(TFSI)_2_ in DEC (cyan) at 100 mA g^−1^ (1st cycle: 10 mA g^−1^) with cut‐off potentials of 3.4 and 5.0 V vs. Li|Li^+^ (procedure 1, Table S2a). The Coulombic efficiencies of the first cycle (Mg‐DMC: 73.1±0.7 %, Mg‐DEC: 76±2 %) are not shown. (c) Corresponding differential capacity versus potential plots of the 1st and 50th cycle.

To gain deeper insights into the solution structure of Mg‐DMC and Mg‐DEC, Raman spectra of the electrolytes as well as the pure solvents were measured (Figures 9a and S17, Tables S12 and S13). Besides the Raman bands between 740 and 760 cm^−1^ (Figure 9a, left side; Figure S17), which reflect the expansion and contraction of the TFSI^−^ anion and give insights into the coordination sphere of the TFSI^−^,^[34a]^ also the coordination of the carbonate solvents can be investigated by the C−O stretching mode between 830 and 960 cm^−1^ (Figure 9a, right side).^[48]^ In good agreement with the Raman spectra of Mg‐Pyr and Mg‐Pyr+ES, three main Raman bands at 743±1, 746±1, and 752±1 cm^−1^ could also be observed for Mg‐DMC and Mg‐DEC (Figure S17 and Table S12). Even though the solution structures in carbonate‐based systems are presumably different to the ones in ionic liquid‐based systems, the association of “free” TFSI^−^, monodentate CIP or bridging TFSI^−^ in AGG, and bidentate TFSI^−^ as described above is also likely in this system.^[34a]^ Based on the lower amount of TFSI^−^ per Mg^2+^ (2 : 1), compared to the ionic liquid‐based systems (>8 : 1), the amount of “free” TFSI^−^ (742 cm^−1^) only has a share of about 30–40 % of the total amount compared to around 70 % for Mg‐Pyr (Tables S8 and S12). In addition, the amount of bidentate coordinated TFSI^−^ (752 cm^−1^) is higher in Mg‐DEC compared to Mg‐DMC, which could be based on a lower molar solvent/Mg^2+^ ratio in Mg‐DEC (4.1 : 1) compared to Mg‐DMC (4.8 : 1), but also increased steric hindrance of the DEC molecule and therefore preferred bidentate TFSI^−^ coordination. In addition, the coordination of the carbonate solvents (Figure 9a, right side, Table S13) can indicate the solution structure. The Raman bands of DMC in Li‐ion‐based electrolytes are well investigated via Raman spectroscopy and additional computational methods in the literature.^[48]^ In pure DMC, one main band at 917 cm^−1^, corresponding to a *cis‐cis* conformer of DMC (*cc*‐DMC) is visible. In addition, a weak band at 861 cm^−1^ indicating a *cis‐trans* conformer of DMC (*ct*‐DMC) appears.[^48b^, ^48c^] Upon the addition of Mg(TFSI)_2_ (Mg‐DMC), two additional peaks at 882 and 945 cm^−1^, corresponding to *ct*‐DMC, respectively *cc*‐DMC coordinated to a cation appear.^[48c]^ Even though still some “free” DMC, indicated by the bands at 862 (weak) and 917 cm^−1^, is present in Mg‐DMC, the intensity of the Raman bands associated with coordinated DMC is much higher. In good agreement with previous reports[^48a^, ^48c^, ^48d^] of Li^+^ in DMC, upon coordination to Mg^2+^, the *ct*‐DMC conformation is preferred, which is clearly shown in the share of areal intensity *I* of the *ct*‐DMC for the uncoordinated bands in pure DMC [*I*_861_/(*I*_861_+*I*_917_)=3 %], compared to the coordinated bands in Mg‐DMC [*I*_882_/(*I*_882_+*I*_945_)=77 %]. In addition, the blue shift upon coordination, indicating the coordination strength is with 28 cm^−1^ for the *cc*‐DMC and 21 cm^−1^ for *ct*‐DMC higher than literature reports for Li‐ion‐based systems (e. g., *cc*‐DMC: 14 cm^−1^, *ct*‐DMC: 13 cm^−1^ by Kameda et al.^[48c]^ or ≈20 cm^−1^ for *cc*‐DMC by Heckmann et al.^[9a]^). These results indicate a higher coordination strength of Mg‐DMC complexes, probably induced by the divalent charge of Mg^2+^ in comparison to monovalent Li^+^. Following this trend, an even lower blue‐shift of *cc*‐DMC was observed for the much larger Na^+^ in DMC.^[48e]^ The Raman spectra of DEC are not as well investigated as the ones of DMC, but in addition to the well described C−O stretching band of the *cis‐cis* conformer of DEC (*cc*‐DEC) at 903 cm^−1^,[^48e^, ^49^] a weak band at 854 cm^−1^ was observed, which might, similar to DMC, indicate a second *cis‐trans* conformer of the DEC (*ct*‐DEC). In Mg‐DEC, two additional peaks arise at 857 and 913 cm^−1^, indicating Mg^2+^‐coordinated DEC.[^48e^, ^49^] Similar to the change of preferred conformation in DMC, also the ratio of intensities of the two observed conformers change drastically from the pure DEC [*I*_854_/(*I*_854_+*I*_903_)=9 %] to Mg‐DEC [*I*_857_/(*I*_857_+*I*_913_)=78 %]. Even though, again, the blue shift is more severe for Mg‐DEC (up to 10 cm^−1^) in comparison to 7 cm^−1^ described for Li‐ion‐based systems,^[49]^ the much lower shift in comparison to the DMC‐based system indicates a weaker Mg‐solvent interaction,^[48e]^ which might be due to the larger size of DEC and could result in more sterically hindered interactions. Overall, it can be concluded, that in both Mg‐DMC and Mg‐DEC, SSIP, CIP, and AGG are present. Even though a higher coordination strength, especially in bidentate CIPs (752 cm^−1^), between TFSI^−^ and Mg^2+^ is expected, the occurrence of SSIPs might be beneficial regarding desolvation energies and therefore the overpotential for TFSI^−^ intercalation.

The cycling performance of graphite ‖ AC cells with Mg‐DMC and Mg‐DEC (Figure 9b,c, and Table S3) is with average SDCs within 40±6 mAh g^−1^ and C_Eff_>99 % comparable to cells containing Mg‐Pyr (Figure 2). Only the first cycle shows a higher SDC (46±3 mAh g^−1^), which is mainly based on the lower onset potential (Table S4, Mg‐Pyr: 4.82±0.01 V vs. Li|Li^+^; Mg‐DMC: 4.61±0.01 V vs. Li|Li^+^; Mg‐DEC: 4.64±0.01 V vs. Li|Li^+^). Still, the onset potentials of higher cycles are within 4.51±0.04 V vs. Li|Li^+^ comparably higher than the ones in cells with Mg‐Pyr (within 4.46±0.03 V vs. Li|Li^+^). The reduced onset potential of the first cycle in comparison to cells cycled with Mg‐Pyr is again accompanied by a significantly lower contact angle on Si (Mg‐Pyr: 38±5°; Mg‐DMC and Mg‐DEC: below 20° after 180 s), likely also improving the electrode wetting. Still, also the different solution structures (Figure 9a) could have an impact on this performance.

**Figure 10:**
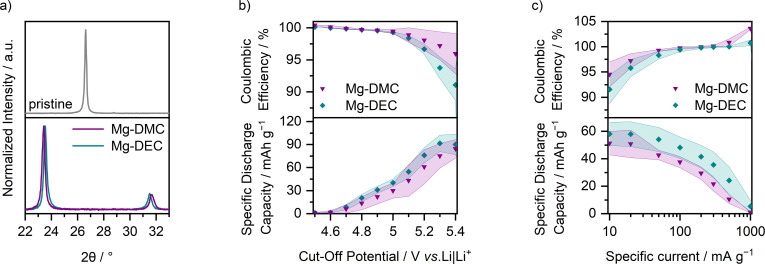
(a) Normalized ex‐situ XRD patterns of pristine graphite composite electrodes (grey), and electrodes cycled in graphite ‖ AC Swagelok‐type cells (three‐electrode configuration; QRE: Li metal) with 2.5 m Mg(TFSI)_2_ in DMC (Mg‐DMC, purple) and 2 m Mg(TFSI)_2_ in DEC (Mg‐DEC, cyan) after three cycles at 10 mA g^−1^ with cut‐off potentials of 3.4 and 5.0 V vs. Li|Li^+^ and one charge to 5.0 V vs. Li|Li^+^ (procedure 6, Table S2d, electrodes in charged state). Coulombic efficiency and specific discharge capacity of graphite ‖ AC Swagelok‐type cells (three‐electrode configuration; QRE: Li metal) with 2.5 m Mg(TFSI)_2_ in DMC (purple) and 2 m Mg(TFSI)_2_ in DEC (cyan) (b) at different cut‐off potentials at a specific current of 100 mA g^−1^ (procedure 4, Table S2b, the values of the 10th cycle of each potential are displayed) and (c) at different specific currents with cut‐off potentials of 3.4 and 5.0 V vs. Li|Li^+^ (procedure 5, Table S2c, the values of the 5th cycle of each current are displayed).

The differential capacity versus potential plots of the first charge of cells with Mg‐DMC and Mg‐DEC (Figure 9c) are quite similar, even though slightly shifted, and show three peaks for TFSI^−^ intercalation at 4.6–4.7 V, 4.7–4.8 V, and above 4.9 V vs. Li|Li^+^. For TFSI^−^ deintercalation, instead, the differential capacity versus potential plots of cells with Mg‐DMC and Mg‐DEC differ. While the cell with Mg‐DEC shows a small peak at 4.7 V vs. Li|Li^+^ and a broad peak between 4.6 and 4.3 V vs. Li|Li^+^, the cells with Mg‐DMC shows three peaks at 4.8, 4.7, and 4.5 V vs. Li|Li^+^, as well as a broad peak between 4.4 and 4.1 V vs. Li|Li^+^. A similar trend can be observed for later cycles. While cells with Mg‐DMC show three distinct TFSI^−^‐intercalation peaks at 4.6, 4.7–4.8, and above 4.9 V vs. Li|Li^+^, only a broad peak from 4.6 to 5.0 V vs. Li|Li^+^ is visible for the cells with Mg‐DEC. In addition, for TFSI^−^ deintercalation, three small peaks at 4.8, 4.6, and 4.5 V vs. Li|Li^+^ and one broad peak between 4.3 and 3.9 V vs. Li|Li^+^ are visible for cells with Mg‐DMC, while mainly one broad peak between 4.5 and 4.0 V vs. Li|Li^+^ can be observed for Mg‐DEC. This indicates on one hand that the kinetics with Mg‐DEC are more sluggish compared to Mg‐DMC, but also that the overpotentials, at least for certain staging of the TFSI^−^‐(de‐)intercalation, are higher using Mg‐DMC since a larger voltage hysteresis can be observed. These differences might arise from the differences in the solution structure, as discussed above (Figure 9a). Still, XRD patterns (Figure 10a, Table S14) reveal, that at 5.0 V vs. Li|Li^+^, the intercalation stages with both electrolytes are similar, with mainly stage 2 graphite present, matching the overall similar cycling performances also reflected in similar SDCs and C_Eff_ (Figure 9b).

**Figure 11:**
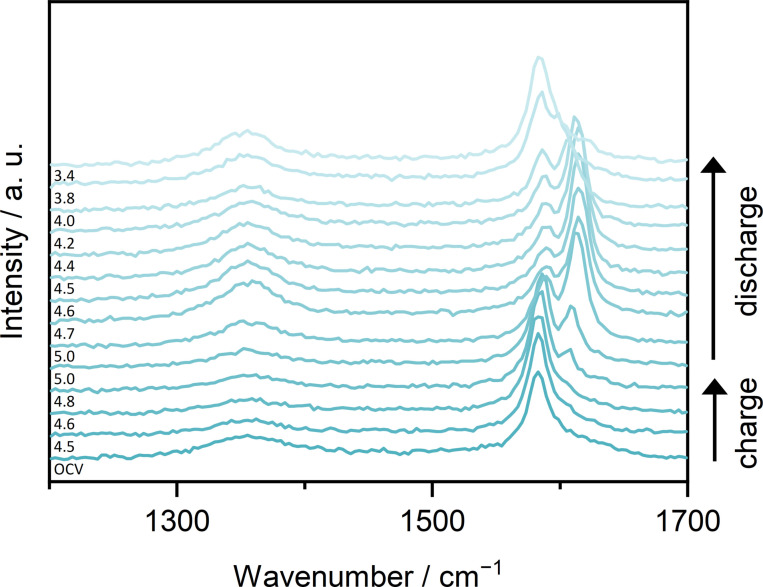
In‐situ Raman spectra at selected potentials of the 2nd cycle of modified graphite ‖ AC pouch‐type cells (three‐electrode configuration; RE/QRE: Li metal) between 1200 and 1700 cm^−1^ with Mg‐DEC at a laser intensity of 1 % (acquisition time: 5×30 s). The corresponding CVs (scan speed: 0.5 mV s^−1^, cut‐off potentials of 3.4–5.0 V vs. Li|Li^+^) are displayed in Figure S18. At the upper potential of 5.0 V vs. Li|Li^+^, two spectra recorded before and after a constant potential step of 5 min are shown.

In‐situ Raman spectra of modified pouch cells with Mg‐DEC are given in Figure 11. Starting from a potential of 4.8 V, the arising *E*_2g2_(b) mode band at around 1608 cm^−1^ indicates the intercalation of TFSI^−^ into the graphite. Again, this potential is higher than the onset potential of the second cycle but rather fits the potential observed in the first cycle (Figure S18), indicating that not all graphite was fully charged in the first cycle. At the upper cut‐off potential, the occurrence of the *E*_2g2_(i) band aside the *E*_2g2_(b) band indicates that stage 2 is not fully reached. Different from the electrolytes based on ionic liquids (Figures 3 and 6), however, the intensity of the D band does not increase significantly upon discharging and the broadening of the G band at the end of the cycle is less pronounced. A similar effect can be observed in the ex‐situ Raman spectra of electrodes after 100 cycles with Mg‐DEC (Figure S7, cyan). While the Pyr_14_TFSI‐based electrolytes show a clear second Raman band at around 1605 cm^−1^ even in the discharged state, it cannot be observed in the Raman spectrum of the electrode cycled with Mg‐DEC. In addition, a much higher intensity of the *E*_2g2_(i) mode band in the charged state in comparison to the *E*_2g2_(b) mode band indicates a lower staging. However, this was not observed in the in‐situ spectra and might indicate a stronger self‐discharge.

SEM images of electrodes cycled in Mg‐DMC and Mg‐DEC (Figure S7) do not show significant alteration, for example, by exfoliation of graphite.

Application of higher cut‐off potentials to cells with Mg‐DMC and Mg‐DEC show similar trends compared to the ionic liquid‐based electrolytes described above. Higher cut‐offs lead to higher SDCs, even though the C_Eff_ are reduced (Figure 10b and Table S6; Mg‐DMC: 5.0 V vs. Li|Li^+^: 30±10 mAh g^−1^, 99.5±0.3 %, 5.3 V vs. Li|Li^+^: 75±16 mAh g^−1^, 97±2 %; Mg‐DEC: 5.0 V vs. Li|Li^+^: 40±5 mAh g^−1^, 99.3±0.1 %, 5.3 V vs. Li|Li^+^: 92±11 mAh g^−1^, 94±2 %). The C_Eff_ at higher cut‐off potentials might increase if higher concentrations are used, as parasitic side reactions might be reduced when free carbonates are absent. Notably, this would presumably come at the cost of decreased capacity and C‐rate performance. However, since no severe electrolyte decomposition or Al‐dissolution, indicated by a large irreversible potential plateau during charge as observed for diluted electrolytes^[50]^ (compare Figure S16) can be observed up to 5.4 V vs. Li|Li^+^ (C_Eff_>90 %), the herein chosen salt concentrations seem to be sufficient for the typical potential range of DIBs (up to ≈5.3 V vs. Li|Li^+^), even though free carbonate solvents were observed in the Raman spectra (Figure 9a). The SDCs are slightly higher for the cells with Mg‐DEC, but, considering the quite large error margin of Mg‐DMC, are probably within the same range. While a higher error margin could also be observed for higher cut‐off potentials for ionic liquid‐based electrolytes, it is not clear why particularly the cells with Mg‐DMC have high deviations, even at lower potentials. The much higher SDCs at high cut‐off potentials are mainly based on additional TFSI^−^ intercalation/deintercalation peaks at 5.1/4.7–4.6 V vs. Li|Li^+^ (Figure S19), respectively lower overpotentials.^[41]^ Again, these peaks are broader for cells with Mg‐DEC in comparison to the ones with Mg‐DMC. Overall, the observed intercalation peaks are in good agreement, even though shifted for about 0.2 V, with previous reports of highly concentrated LiTFSI‐based carbonate electrolytes,^[9a]^ which show TFSI^−^‐intercalation peaks between 4.3–4.6, 4.7–4.8. and 4.9 V vs. Li|Li^+^ and highly concentrated KFSI‐based carbonate electrolytes^[12c]^ (intercalation: ≈4.6, 4.75, and 4.9 V vs. K/K^+^; deintercalation: ≈4.75 and 4.6–4.3 V vs. K/K^+^). Interestingly, the overall intercalation behavior is similar to the performance of cells with Mg‐Pyr+ES (Figure S14a), indicating that similar kinetic effects occur. The performance at different specific currents (Figures 10c and S20, Table S7) follows a similar trend as for the ionic liquid‐based electrolytes, with higher SDCs but lower C_Eff_ at low currents (Mg‐DMC: 10 mA g^−1^: 51±9 mAh g^−1^, 95±3 %; 100 mA g^−1^: 38±4 mAh g^−1^, 99.8±0.2 %; Mg‐DEC: 10 mA g^−1^: 58±8 mAh g^−1^, 92±3 %; 100 mA g^−1^: 48±8 mAh g^−1^, 99.4±0.4 %), probably based on increasing side reactions, but much lower SDCs and higher C_Eff_ at high specific currents, caused by higher overpotentials for (de‐)intercalation (Figure S20, Mg‐DMC: 500 mA g^−1^: 11±3 mAh g^−1^, 100.8±0.7 %; Mg‐DEC: 500 mA g^−1^: 24±10 mAh g^−1^, 100.0±0.1 %). In comparison to the cells with Mg‐Pyr and Mg‐Pyr+ES, which did not show significant onset potential shifts between 10 and 100 mA g^−1^ (Figure S15), the overpotentials of the carbonate‐based electrolytes are already increased comparing 100 and 10 mA g^−1^ (Figure S20), indicating sluggish kinetics of the highly concentrated electrolytes. This is also reflected in the diminished performance at higher specific currents: While the ionic liquid‐based systems showed only a slow decrease in SDCs up to 1000 mA g^−1^, the SDCs are decreasing more significantly with the specific current (Figure 10c, Table S7). The values at low currents on the other hand, especially for cells with Mg‐DEC are with 54–58±9 mAh g^−1^ rather comparable to the values of cells with Mg‐Pyr+ES, also reflecting the similarities in the differential capacity versus potential plots.

Overall, with slightly higher SDCs and C_Eff_ as well as lower overpotentials (especially for deintercalation) the performance of cells containing Mg‐DEC is better than the ones with Mg‐DMC. In addition, the lower amount of required conducting salt (2 m in Mg‐DEC versus 2.5 m in Mg‐DMC) is beneficial regarding the cost. Notably, highly concentrated electrolytes are only suitable if low currents (≤100 mA g^−1^) are applied. In this case, a slightly higher salt concentration might also be used to achieve an increased C_Eff_ at higher cut‐off potentials.

## Conclusion

In this work, a bis(trifluoromethanesulfonyl)imide anion (TFSI^−^) intercalating graphite cathode for the possible application in Mg‐ion‐based DIBs was systematically evaluated using activated carbon‐based (AC) counter electrodes (CE). In the first part, the comparison of Mg(TFSI)_2_ in Pyr_14_TFSI to a Li‐ion‐based system revealed, in good agreement with our previous results,^[12k]^ that, except for the first intercalation, no great differences between Mg‐ and Li‐ion‐based systems occur, even though Raman spectra and theoretical calculations from the literature^[35]^ suggest higher binding energies between Mg^2+^ and TFSI^−^ compared to the Li^+^ counterparts.

Secondly, and to the best of our knowledge for the first time, ethylene sulfite (ES) was introduced as additive into a Mg‐ion‐based DIB and showed an improvement of the discharge capacity, most likely based on lower overpotentials for TFSI^−^ intercalation as well as on wetting effects, which was also observed for alkali‐based systems upon the addition of ES.

Thirdly, and to the best of our knowledge also for the first time, highly concentrated carbonate‐based electrolytes were successfully introduced to Mg‐ion‐based DIBs. Even though they showed sluggish kinetics at higher specific currents, especially at low specific currents the SDCs outperformed the ionic liquid‐based system without additives, while having a similar C_Eff_.

Since in this study AC was used as CE, the feasibility of this electrolytes in DIBs with Mg metal still needs to be proven. Even though attempts were made to use alternative Mg^2+^‐storage anodes for Mg‐DIBs,[^12i^, ^12j^] their capacity is still limited. However, the use of ES as additive or highly concentrated carbonate‐based electrolytes might also be beneficial in these systems.

In the past years, several promising attempts to protect the Mg surface with an artificial solid electrolyte interphase (SEI) have been made^[33]^ and even allowed the use of oxidatively more stable halogen‐free ether‐based, or carbonate‐based electrolytes. Therefore, we strongly consider the development of an artificial, well Mg^2+^ conductive SEI, which would enable the application of a Mg‐metal anode with the here investigated electrolytes.

## Experimental Section

### Electrodes and electrolytes

All electrolyte formulations are listed in Table 1. Electrolytes were obtained by mixing 1 mol per L solvent [m] LiTFSI (Solvionic, 99.9 %) or 0.5 m Mg(TFSI)_2_ (Solvionic, 99.5 %) in 1‐butyl‐1‐methylpyrrolidinium bis(trifluoromethanesulfonyl)imide (Pyr_14_TFSI, Solvionic, 99.9 %) to obtain Li‐Pyr and Mg‐Pyr with the same molar concentration of TFSI^−^. To obtain Mg‐Pyr+ES, 2 wt% ethylene sulfite (Merck, >99.0 %) was added to Mg‐Pyr. For the concentrated carbonate‐based electrolytes Mg‐DMC and Mg‐DEC, the desired amounts of Mg(TFSI)_2_ (Solvionic, 99.5 %) were dissolved in DMC (Powerlyte, battery grade) or DEC (Powerlyte, battery grade), respectively. For the calibration of the Li metal RE, 0.05 m ferrocene (Alfa Aesar, 99+%) was dissolved in the electrolytes prior to cell assembly.

**Table 1:** Abbreviations and electrolyte formulations.^[a]^

Electrolyte abbreviation	Conc. [m]	Salt	Solvent	Additive
Mg‐Pyr	0.5	Mg(TFSI)_2_	Pyr_14_TFSI	–
Li‐Pyr	1	LiTFSI	Pyr_14_TFSI	–
Mg‐Pyr+ES	0.5	Mg(TFSI)_2_	Pyr_14_TFSI	2 wt % ES
Mg‐DMC	2.5	Mg(TFSI)_2_	DMC	–
Mg‐DEC	2	Mg(TFSI)_2_	DEC	–

[a] The abbreviations consist of cation‐solvent+additive. Pyr=Pyr_14_TFSI.

LiTFSI, Mg(TFSI)_2_, and Pyr_14_TFSI were dried for at least 24 h under high‐vacuum conditions (<1×10^−6^ mbar) at 110 °C. All other chemicals were used as received.

Working electrodes were prepared by mixing 90 wt% of KS6 graphite (Imerys) with 5 wt% of Super C65 (Imerys) and 5 wt% sodium carboxymethylcellulose (CMC, Sigma Aldrich) in an aqueous electrode paste with 30 wt% solids for 1 h by an Ultra‐turrax® disperser at 5000 rpm. Finally, the electrode paste was coated on aluminum foil (20 μm, Goodfellow) by a standard doctor‐blade technique with a doctor‐blade gap of 100 μm and dried at 60 °C for 1 h. 12 mm electrode discs were punched out and dried again at 120 °C at reduced pressure for at least 24 h, resulting in average active mass (graphite) loadings of 1.9±0.2 mg cm^−2^.

Oversized (capacity) self‐standing activated carbon counter electrodes were prepared by mixing 85 wt% of activated carbon (Norit), 10 wt% of Super C65 (Imerys), and 5 wt% of polytetrafluoroethylene (PTFE, Sigma Aldrich, in form of a 60 wt% dispersion in H_2_O) in ethanol for at least 1 h. The electrode paste was then stirred and heated to 80 °C to evaporate excess solvent. The obtained electrode paste was kneaded several minutes to ensure a good distribution and a high density and afterwards rolled flat. Finally, 12 mm electrode discs were punched out and dried at 120 °C at reduced pressure for at least 24 h.

All electrolytes and electrodes were stored in a dry room with atmospheric water contents below 0.02 %.

### Electrochemical investigations

Electrochemical investigations were performed in 3‐electrode Swagelok‐type T‐cells^[47]^ with WE, CE (prepared as described above), and a 5 mm Li metal disk (Albemarle) RE/QRE. WE and CE were separated by a separator (two layers of 13 mm Whatman GF/A) soaked with 170 μL of electrolyte. Additionally, two layers of 10 mm Whatman GF/A soaked with 50 μL of electrolyte were used to separate the WE and CE from the RE/QRE. Cycling performance of these cells was studied on a battery cycler (MACCOR Series 4000, MACCOR INC., procedure 1, 5 and 6, Tables S2a, S2c and S2d) or on a multi‐channel potentiostat (VMP3, Bio‐Logic) including cut‐off potentials above 5.0 V vs. Li|Li^+^ (procedures 2–4, Tables S2a and S2b) at 20 °C. For the constant‐current studies, Swagelok‐type cells were controlled with the potential of the WE within the cut‐off potentials of 3.4–4.9 V (Li‐ion‐based) respectively 5.0 V (Mg‐ion‐based) vs. Li|Li^+^ at specific currents of 100 mA g^−1^ (10 mA g^−1^ for the first cycle, procedure 1, Table S2a). Herein, the values in mA g^−1^ and mAh g^−1^ refer to the weight of graphite, or generally, the respective active material of the WE, only. To investigate the performances of the cells at higher cut‐off potentials, after one pre‐cycle [10 mA g^−1^, 3.4–4.9 V (Li‐ion‐based) respectively 5.0 V (Mg‐ion‐based) vs. Li|Li^+^], increasing upper cut‐off potentials (4.5–5.5 V vs. Li|Li^+^ in 0.1 V steps, 10 cycles each; respectively, lower cut‐off potential: 3.4 V vs. Li|Li^+^) were applied (procedure 4, Table S2b). The 10th cycle of each cut‐off potential was used to illustrate the SDCs andC_Eff_. To investigate the performance of the cells in dependency of the specific current, cycle sequences with different specific currents were used: After the preconditioning cycle (10 mA g^−1^) 30 cycles with 100 mA g^−1^ were performed, followed by a range of specific currents [from 10 to 10000 mA g^−1^, 5 cycles each between 3.4–4.9 V (Li‐ion‐based) respectively 5.0 V (Mg‐ion‐based) vs. Li|Li^+^, procedure 5, Table S2c]. In order to illustrate the relation between the specific current and the SDC as well as the C_Eff_, the fifth cycle of each specific current was used. Long term cycling with cut‐off potentials of 5.3 V vs. Li|Li^+^ were obtained at specific currents of 100 mA g^−1^ (procedure 2, Table S2a) respectively 300 mA g^−1^ (procedure 3, Table S2a; both with 10 mA g^−1^ and an upper cut‐off potential of 5.0 V vs. Li|Li^+^ for the first cycle) on a multi‐channel potentiostat (VMP3, Bio‐Logic). For each electrochemical investigation, at least three independent cells were evaluated to ensure a high reproducibility of the electrochemical data, which is indicated by error bars in the respective graphs.

For the calibration of the Li‐metal RE, Pt ‖ AC Swagelok‐type cells with a Li‐metal RE were used. The potential of ferrocene was calculated according to Liu et al.^[31]^ from CV measurements at 5 mV s^−1^ on a multi‐channel potentiostat (VMP3, Bio‐Logic) at 20 °C.

### X‐ray diffraction

Prior to the XRD measurements, the graphite electrodes were prepared by three constant‐current cycles at specific currents of 10 mA g^−1^ in Swagelok‐type cells with controlled potential of the WE within the cut‐off potentials 3.4–4.9 V (Li‐ion‐based) respectively 5.0 V (Mg‐ion‐based) vs. Li|Li^+^. Following, the cell was charged to the displayed potential with a scan speed of 10 mA g^−1^ (100 mA g^−1^ for the cut‐off potential of 5.3 V vs. Li|Li^+^). Afterwards, the charged cells were disassembled under ambient air, and the unwashed graphite electrodes were put on a sample holder (single‐crystal silicon), fixed with Kapton‐tape and measured within 3 h.

Ex‐situ XRD of the graphite electrodes was measured at room temperature on a Bruker D8 Advance diffractometer with CuK_α_ radiation (1.54 Å) within the 2*θ* range of 20–35° with a step size of 0.020°. The background of each data set was subtracted before analysis and data extraction using the software Diffrac.Eva 3.1 (Bruker).

### Drop‐shape analysis

For contact angle measurements, a Krüss Drop Shape Analyzer DSA 100 was used. All measurements were performed at room temperature in a dry room with water contents below 0.02 %. The contact angles between different electrolytes (Mg‐Pyr, Li‐Pyr, Mg‐Pyr+ES, Mg‐DMC, and Mg‐DEC) in form of sessile drops and the Si wafer or graphite composite electrodes were obtained using the tangent method 2 in the DSA 1 software.

### Raman spectroscopy

Raman spectra of different electrolytes were obtained using 5 mm NMR‐tubes (Bruker) on a VERTEX 70 FT‐IR spectrometer (Bruker) with a RAM II FT‐Raman Module (Bruker), a nitrogen‐cooled Ge‐diode detector and a 1064 nm laser source with the OPUS 7.0 software. The spectra are normalized to the maximal intensity of the Raman spectra at around 742 cm^−1^ for the spectra shown between 720 and 780 or 800 cm^−1^, respectively the maximal intensity of the Raman spectra between 830 and 960 cm^−1^. The band positions and areas were calculated using PsdVoigt1 functions in the OriginPro 2019 software. According to Watkins et al.,^[34b]^ the shape factors of the TFSI^−^ bands were set to 0.85 for the band at around 742 cm^−1^ (“free” TFSI^−^) and 0.5 for the higher‐frequency bands (coordinated TFSI^−^). Each fitting was performed three times with a *R* ^2^ of at least 0.998 and the resulting average values and standard deviation are displayed.

For the in‐situ Raman measurements, a modified pouch cell setup was used. A schematic illustration of the cell setup is given in Figure S21. The aqueous electrode paste of the graphite WE was prepared similar to the ones used in Swagelok‐type cells described above, but with a KS6 graphite/Super C65/CMC ratio of 70 : 10 : 20 and a solid wt % of around 11 %. The electrodes were prepared by applying 2×5 μL of the electrode paste on cover glass (Thermo Scientific, Ø=30 mm, #2) covered with a stripe of Al mesh (5 mm wide) as current collector. The electrodes were dried at 60 °C and afterwards fixed on a pouch foil by pressing it on the pouch foil with a hot piece of metal and adhesive tape to improve the air tightness. A 10×5 mm self‐standing AC CE was pressed on an Al mesh as current collector. For the RE a 100 μm thick 5×5 mm Li metal piece on a carbon‐coated copper mesh (Benmetal) was used, proceeded from 500 μm thick Li metal (Albemarle, battery grade) using a roll press (Hohsen Corp., HSAM‐615H), as described previously.[^42^, ^51^] The pouch cells consisted of the glass‐graphite WE, the Li metal RE between two separators (16 mm Whatman GF/A) soaked with 200 μL of electrolyte, and the AC CE, wrapped in pouch foil and hermetically sealed under reduced pressure (compare Figure S21). The cells were cycled after a rest period of at least 12 h (stored in a dry room) with a Solartron SI 1287 potentiostat using CV and constant‐potential steps at selected potentials. In the first cycle, the cell was charged from OCP to a potential of 4.9 V (Li‐ion‐based), respectively 5.0 V vs. Li|Li^+^ (Mg‐ion‐based) at a scan rate of 0.5 mV s^−1^. The potential was then kept at the upper potential for 30 min and discharged to 3.4 V vs. Li|Li^+^ with 0.5 mV s^−1^ and kept at 3.4 V vs. Li|Li^+^ for 5 min. The cells were then transferred to a LabRAM HR Evolution, Horiba Scientific with a green Nd:YAG laser (532 nm) and a 50× magnification long distance objective (Olympus). For the second in‐situ cycle, the cells were charged and discharged from 3.4 to 4.9 V (Li‐ion‐based), respectively 5.0 V vs. Li|Li^+^ (Mg‐ion‐based) at a scan rate of 0.5 mV s^−1^, with a constant potential step of 5 min at the upper potential. To avoid local heating and electrolyte degradation, during the in‐situ Raman measurements the laser intensity was set to 1 % and the spectra were recorded with 5×30 s acquisition time. The displayed potentials reflect the potential, at which the measurement of the displayed spectra was started. Before each measurement the *z* direction was focused (autofocus) using the maximal spectral intensity between 1550 and 1650 cm^−1^.

For the ex‐situ Raman spectra of cycled electrodes, graphite ‖ AC Swagelok‐type cells (three‐electrode configuration; RE/QRE: Li metal) were cycled using procedure 1 (Table S2a) with only 100 repetitions at 100 mA g^−1^. For the charged electrodes, the cells were afterwards charged to 4.9 V (Li‐ion‐based), respectively 5.0 V vs. Li|Li^+^ (Mg‐ion‐based) at 100 mA g^−1^. The cells were disassembled in a dry room and the electrodes not washed prior to the Raman measurements. To avoid moisture contact, the electrodes were transported and measured in a sealed container with a glass window. For the ex‐situ Raman spectra, the laser intensity was set to 10 % with 60×5 s acquisition time to obtain a better signal to noise ratio. The obtained spectra using the two different laser intensities and acquisition times for ex‐situ and in‐situ Raman spectra differ only in the absolute intensity and signal‐to‐noise ratio, but not in the relative Raman band intensities or positions, as shown in Figure S5.

The background of all in‐situ and ex‐situ Raman spectra was corrected by setting the first intensity value at around 1200 cm^−1^ to zero.

### Scanning electron microscopy

SEM images were obtained using an Auriga field emission scanning electron microscope (FE‐SEM) Crossbeam workstation equipped with a Schottky field emission gun by Carl Zeiss AG using an acceleration voltage of 3 kV, an in‐lens secondary electron detector, and a working distance of 5 mm. For the SEM measurements, the cycled graphite electrodes measured by ex‐situ Raman spectroscopy were used. To remove the electrolyte surface film, the electrodes were washed with DMC (2×0.5 mL) and transferred to the SEM device in a sealed container without moisture contact.

## Conflict of interest

The authors declare no conflict of interest.

## Supporting information

As a service to our authors and readers, this journal provides supporting information supplied by the authors. Such materials are peer reviewed and may be re‐organized for online delivery, but are not copy‐edited or typeset. Technical support issues arising from supporting information (other than missing files) should be addressed to the authors.

Supporting Information

## References

[1] R. Schmuch , R. Wagner , G. Hörpel , T. Placke , M. Winter , Nat. Energy 2018, 3, 267–278.

[2a] S. Dühnen , J. Betz , M. Kolek , R. Schmuch , M. Winter , T. Placke , Small Methods 2020, 4, 2000039;

[2b] M. Winter , B. Barnett , K. Xu , Chem. Rev. 2018, 118, 11433–11456. 30500179 10.1021/acs.chemrev.8b00422

[3] J. Muldoon , C. B. Bucur , T. Gregory , Angew. Chem. Int. Ed. 2017, 56, 12064–12084; 10.1002/anie.201700673 28295967

[4a] D. Aurbach , Z. Lu , A. Schechter , Y. Gofer , H. Gizbar , R. Turgeman , Y. Cohen , M. Moshkovich , E. Levi , Nature 2000, 407, 724–727; 11048714 10.1038/35037553

[4b] A. Ponrouch , J. Bitenc , R. Dominko , N. Lindahl , P. Johansson , M. R. Palacin , Energy Storage Mater. 2019, 20, 253–262;

[4c] M. Mao , T. Gao , S. Hou , C. Wang , Chem. Soc. Rev. 2018, 47, 8804–8841. 30339171 10.1039/c8cs00319j

[5a] J. Betz , G. Bieker , P. Meister , T. Placke , M. Winter , R. Schmuch , Adv. Energy Mater. 2019, 9, 1803170;

[5b] T. Gao , S. Hou , F. Wang , Z. Ma , X. Li , K. Xu , C. Wang , Angew. Chem. Int. Ed. 2017, 56, 13526–13530; 10.1002/anie.201708241 28849616

[5c] C. Pei , F. Xiong , Y. Yin , Z. Liu , H. Tang , R. Sun , Q. An , L. Mai , Small 2021, 17, 2004108. 10.1002/smll.202004108 33354934

[6a] T. S. Arthur , N. Singh , M. Matsui , Electrochem. Commun. 2012, 16, 103–106;

[6b] Z. Meng , D. Foix , N. Brun , R. Dedryvère , L. Stievano , M. Morcrette , R. Berthelot , ACS Energy Lett. 2019, 4, 2040–2044;

[6c] J. Niu , Z. Zhang , D. Aurbach , Adv. Energy Mater. 2020, 10, 2000697;

[6d] R. Dominko , J. Bitenc , R. Berthelot , M. Gauthier , G. Pagot , V. Di Noto , J. Power Sources 2020, 478, 229027.

[7] Z. Guo , S. Zhao , T. Li , D. Su , S. Guo , G. Wang , Adv. Energy Mater. 2020, 10, 1903591.

[8a] X. Yu , A. Manthiram , Adv. Funct. Mater. 2020, 30, 2004084;

[8b] G. Bieker , V. Küpers , M. Kolek , M. Winter , Commun. Mater. 2021, 2, 37;

[8c] P. Wang , M. R. Buchmeiser , Adv. Funct. Mater. 2019, 29, 1905248;

[8d] S.-H. Chung , A. Manthiram , Adv. Mater. 2019, 31, 1901125. 10.1002/adma.201901125 31081272

[9a] A. Heckmann , J. Thienenkamp , K. Beltrop , M. Winter , G. Brunklaus , T. Placke , Electrochim. Acta 2018, 260, 514–525;

[9b] L. Xiang , X. Ou , X. Wang , Z. Zhou , X. Li , Y. Tang , Angew. Chem. Int. Ed. 2020, 59, 17924–17930; 10.1002/anie.202006595 32558980

[9c] K. V. Kravchyk , P. Bhauriyal , L. Piveteau , C. P. Guntlin , B. Pathak , M. V. Kovalenko , Nat. Commun. 2018, 9, 4469. 30367050 10.1038/s41467-018-06923-6 PMC6203722

[10a] T. Placke , P. Bieker , F. Lux Simon , O. Fromm , H.-W. Meyer , S. Passerini , M. Winter , Z. Phys. Chem. 2012, 226, 391–407;

[10b] T. Placke , O. Fromm , S. F. Lux , P. Bieker , S. Rothermel , H.-W. Meyer , S. Passerini , M. Winter , J. Electrochem. Soc. 2012, 159, A1755–A1765.

[11a] J. M. Wrogemann , S. Künne , A. Heckmann , I. A. Rodríguez-Pérez , V. Siozios , B. Yan , J. Li , M. Winter , K. Beltrop , T. Placke , Adv. Energy Mater. 2020, 10, 1902709;

[11b] Y. Kondo , Y. Miyahara , T. Fukutsuka , K. Miyazaki , T. Abe , Electrochem. Commun. 2019, 100, 26–29.

[12a] K. Beltrop , S. Beuker , A. Heckmann , M. Winter , T. Placke , Energy Environ. Sci. 2017, 10, 2090–2094;

[12b] P. Meister , O. Fromm , S. Rothermel , J. Kasnatscheew , M. Winter , T. Placke , Electrochim. Acta 2017, 228, 18–27;

[12c] P. Münster , A. Heckmann , R. Nölle , M. Winter , K. Beltrop , T. Placke , Batteries & Supercaps 2019, 2, 992–1006;

[12d] G. A. Elia , I. Hasa , G. Greco , T. Diemant , K. Marquardt , K. Hoeppner , R. J. Behm , A. Hoell , S. Passerini , R. Hahn , J. Mater. Chem. A 2017, 5, 9682–9690;

[12e] S. Wang , S. Jiao , W.-L. Song , H.-S. Chen , J. Tu , D. Tian , H. Jiao , C. Fu , D.-N. Fang , Energy Storage Mater. 2018, 12, 119–127;

[12f] E. Zhang , W. Cao , B. Wang , X. Yu , L. Wang , Z. Xu , B. Lu , Energy Storage Mater. 2018, 11, 91–99;

[12g] X. Zhang , Y. Tang , F. Zhang , C.-S. Lee , Adv. Energy Mater. 2016, 6, 1502588;

[12h] S. Wu , F. Zhang , Y. Tang , Adv. Sci. 2018, 5, 1701082; 10.1002/advs.201701082 PMC6097003 30128228

[12i] X. Lei , Y. Zheng , F. Zhang , Y. Wang , Y. Tang , Energy Storage Mater. 2020, 30, 34–41;

[12j] R. Yang , F. Zhang , X. Lei , Y. Zheng , G. Zhao , Y. Tang , C.-S. Lee , ACS Appl. Mater. Interfaces 2020, 12, 47539–47547; 32986396 10.1021/acsami.0c13045

[12k] P. Meister , V. Küpers , M. Kolek , J. Kasnatscheew , S. Pohlmann , M. Winter , T. Placke , Batteries & Supercaps 2021, 4, 504–512;

[12l] B. Ji , W. Yao , Y. Tang , Sustain. Energy Fuels 2020, 4, 101–107.

[13a] K. Beltrop , P. Meister , S. Klein , A. Heckmann , M. Grünebaum , H.-D. Wiemhöfer , M. Winter , T. Placke , Electrochim. Acta 2016, 209, 44–55;

[13b] J. A. Seel , J. R. Dahn , J. Electrochem. Soc. 2000, 147, 892–898;

[13c] M.-C. Lin , M. Gong , B. Lu , Y. Wu , D.-Y. Wang , M. Guan , M. Angell , C. Chen , J. Yang , B.-J. Hwang , H. Dai , Nature 2015, 520, 324–328; 10.1038/nature14340 25849777

[13d] J. Gao , M. Yoshio , L. Qi , H. Wang , J. Power Sources 2015, 278, 452–457.

[14] S. Rothermel , P. Meister , G. Schmuelling , O. Fromm , H.-W. Meyer , S. Nowak , M. Winter , T. Placke , Energy Environ. Sci. 2014, 7, 3412–3423.

[15a] T. Placke , S. Rothermel , O. Fromm , P. Meister , S. F. Lux , J. Huesker , H.-W. Meyer , M. Winter , J. Electrochem. Soc. 2013, 160, A1979–A1991;

[15b] M. Kolek , F. Otteny , P. Schmidt , C. Mück-Lichtenfeld , C. Einholz , J. Becking , E. Schleicher , M. Winter , P. Bieker , B. Esser , Energy Environ. Sci. 2017, 10, 2334–2341;

[15c] C. Li , H. Yang , J. Xie , K. Wang , J. Li , Q. Zhang , ACS Appl. Mater. Interfaces 2020, 12, 32719–32725; 32602692 10.1021/acsami.0c07729

[15d] S. Dühnen , R. Nölle , J. Wrogemann , M. Winter , T. Placke , J. Electrochem. Soc. 2019, 166, A5474-A5482;

[15e] C. Li , B. Lao , Z. Li , H. Yin , Z. Yang , H. Wang , D. Chen , X. Zhang , Y. Xu , C. Sun , Energy Storage Mater. 2020, 32, 159–166;

[15f] Z. Guo , Z. Xu , F. Xie , J. Feng , M. Titirici , Adv. Energy Sustainability Res. 2021, 2100074;

[15g] T. Placke , A. Heckmann , R. Schmuch , P. Meister , K. Beltrop , M. Winter , Joule 2018, 2, 2528–2550;

[15h] L. Zhang , H. Wang , X. Zhang , Y. Tang , Adv. Funct. Mater. 2021, 31, 2010958.

[16] X. Qi , B. Blizanac , A. DuPasquier , P. Meister , T. Placke , M. Oljaca , J. Li , M. Winter , Phys. Chem. Chem. Phys. 2014, 16, 25306–25313. 25335810 10.1039/c4cp04113e

[17] G. Schmuelling , T. Placke , R. Kloepsch , O. Fromm , H.-W. Meyer , S. Passerini , M. Winter , J. Power Sources 2013, 239, 563–571.

[18a] Z. Lu , A. Schechter , M. Moshkovich , D. Aurbach , J. Electroanal. Chem. 1999, 466, 203–217;

[18b] G. A. Giffin , J. Mater. Chem. A 2016, 4, 13378–13389;

[18c] G. T. Cheek , W. E. O'Grady , S. Z. El Abedin , E. M. Moustafa , F. Endres , J. Electrochem. Soc. 2008, 155, D91–D95;

[18d] N. Amir , Y. Vestfrid , O. Chusid , Y. Gofer , D. Aurbach , J. Power Sources 2007, 174, 1234–1240;

[18e] G. Vardar , A. E. S. Sleightholme , J. Naruse , H. Hiramatsu , D. J. Siegel , C. W. Monroe , ACS Appl. Mater. Interfaces 2014, 6, 18033–18039. 25248147 10.1021/am5049064

[19a] A. Kitada , Y. Kang , Y. Uchimoto , K. Murase , J. Electrochem. Soc. 2014, 161, D102–D106;

[19b] A. Kitada , Y. Kang , K. Matsumoto , K. Fukami , R. Hagiwara , K. Murase , J. Electrochem. Soc. 2015, 162, D389–D396;

[19c] B. Pan , K.-C. Lau , J. T. Vaughey , L. Zhang , Z. Zhang , C. Liao , J. Electrochem. Soc. 2017, 164, A902–A906;

[19d] V. Küpers , D. Weintz , C. Mück-Lichtenfeld , P. Bieker , M. Winter , M. Kolek , J. Electrochem. Soc. 2020, 167, 160505.

[20a] T. Watkins , A. Kumar , D. A. Buttry , J. Am. Chem. Soc. 2016, 138, 641–650; 26683518 10.1021/jacs.5b11031

[20b] X. Gao , A. Mariani , S. Jeong , X. Liu , X. Dou , M. Ding , A. Moretti , S. Passerini , J. Power Sources 2019, 423, 52–59.

[21a] Y. NuLi , J. Yang , R. Wu , Electrochem. Commun. 2005, 7, 1105–1110;

[21b] Y. NuLi , J. Yang , P. Wang , Appl. Surf. Sci. 2006, 252, 8086–8090.

[22a] I. Shterenberg , M. Salama , H. D. Yoo , Y. Gofer , J.-B. Park , Y.-K. Sun , D. Aurbach , J. Electrochem. Soc. 2015, 162, A7118–A7128;

[22b] S.-Y. Ha , Y.-W. Lee , S. W. Woo , B. Koo , J.-S. Kim , J. Cho , K. T. Lee , N.-S. Choi , ACS Appl. Mater. Interfaces 2014, 6, 4063–4073; 24559269 10.1021/am405619v

[22c] O. Tutusaus , R. Mohtadi , T. S. Arthur , F. Mizuno , E. G. Nelson , Y. V. Sevryugina , Angew. Chem. Int. Ed. 2015, 54, 7900–7904; 10.1002/anie.201412202 26013580

[22d] N. Sa , B. Pan , A. Saha-Shah , A. A. Hubaud , J. T. Vaughey , L. A. Baker , C. Liao , A. K. Burrell , ACS Appl. Mater. Interfaces 2016, 8, 16002–16008; 27255422 10.1021/acsami.6b03193

[22e] G. Bieker , M. Salama , M. Kolek , Y. Gofer , P. Bieker , D. Aurbach , M. Winter , ACS Appl. Mater. Interfaces 2019, 11, 24057–24066; 31199113 10.1021/acsami.9b05307

[22f] J. O. Besenhard , M. Winter , ChemPhysChem 2002, 3, 155–159. 12503122 10.1002/1439-7641(20020215)3:2<155::AID-CPHC155>3.0.CO;2-S

[23] A. L. Lipson , S.-D. Han , B. Pan , K. A. See , A. A. Gewirth , C. Liao , J. T. Vaughey , B. J. Ingram , J. Electrochem. Soc. 2016, 163, A2253–A2257.

[24a] Y. Liang , H. Dong , D. Aurbach , Y. Yao , Nat. Energy 2020, 5, 646–656;

[24b] Z. Zhao-Karger , R. Liu , W. Dai , Z. Li , T. Diemant , B. P. Vinayan , C. Bonatto Minella , X. Yu , A. Manthiram , R. J. Behm , M. Ruben , M. Fichtner , ACS Energy Lett. 2018, 3, 2005–2013;

[24c] H. Xu , Z. Zhang , Z. Cui , A. Du , C. Lu , S. Dong , J. Ma , X. Zhou , G. Cui , Electrochem. Commun. 2017, 83, 72–76;

[24d] A. Du , Z. Zhang , H. Qu , Z. Cui , L. Qiao , L. Wang , J. Chai , T. Lu , S. Dong , T. Dong , H. Xu , X. Zhou , G. Cui , Energy Environ. Sci. 2017, 10, 2616–2625;

[24e] Z. Zhang , Z. Cui , L. Qiao , J. Guan , H. Xu , X. Wang , P. Hu , H. Du , S. Li , X. Zhou , S. Dong , Z. Liu , G. Cui , L. Chen , Adv. Energy Mater. 2017, 7, 1602055;

[24f] V. Bhaghavathi Parambath , Z. Zhao-Karger , T. Diemant , M. Jäckle , Z. Li , T. Scherer , A. Gross , R. J. Behm , M. Fichtner , J. Mater. Chem. A 2020, 8, 22998–23010.

[25] P. Seggem , S. N. Chavan , S. Biswas , V. R. Jetti , ACS Appl. Energy Mater. 2021, 4, 5165–5174.

[26] Q. Zhao , Y. NuLi , T. Nasiman , J. Yang , J. Wang , Int. J. Electrochem. 2012, 2012, 701741.

[27] I. Shterenberg , M. Salama , Y. Gofer , D. Aurbach , Langmuir 2017, 33, 9472–9478. 28651053 10.1021/acs.langmuir.7b01609

[28] B. Özmen-Monkul , M. M. Lerner , Carbon 2010, 48, 3205–3210.

[29a] S. Rothermel , P. Meister , O. Fromm , J. Huesker , H. W. Meyer , M. Winter , T. Placke , ECS Trans. 2014, 58, 15–25;

[29b] G. H. Wrodnigg , J. O. Besenhard , M. Winter , J. Power Sources 2001, 97–98, 592–594.

[30] R. Dugas , J. D. Forero-Saboya , A. Ponrouch , Chem. Mater. 2019, 31, 8613–8628. 31736535 10.1021/acs.chemmater.9b02776 PMC6854841

[31] X. Liu , G. A. Elia , S. Passerini , J. Power Sources Advances 2020, 2, 100008.

[32a] Q. Guo , W. Zeng , S.-L. Liu , Y.-Q. Li , J.-Y. Xu , J.-X. Wang , Y. Wang , Rare Met. 2021, 40, 290–308;

[32b] N. Wu , Y.-C. Lyu , R.-J. Xiao , X. Yu , Y.-X. Yin , X.-Q. Yang , H. Li , L. Gu , Y.-G. Guo , NPG Asia Mater. 2014, 6, e120;

[32c] I. A. Rodríguez-Pérez , Y. Yuan , C. Bommier , X. Wang , L. Ma , D. P. Leonard , M. M. Lerner , R. G. Carter , T. Wu , P. A. Greaney , J. Lu , X. Ji , J. Am. Chem. Soc. 2017, 139, 13031–13037; 28823162 10.1021/jacs.7b06313

[32d] D. Lu , H. Liu , T. Huang , Z. Xu , L. Ma , P. Yang , P. Qiang , F. Zhang , D. Wu , J. Mater. Chem. A 2018, 6, 17297–17302.

[33a] X. Li , T. Gao , F. Han , Z. Ma , X. Fan , S. Hou , N. Eidson , W. Li , C. Wang , Adv. Energy Mater. 2018, 8, 1701728;

[33b] T. Gao , S. Hou , K. Huynh , F. Wang , N. Eidson , X. Fan , F. Han , C. Luo , M. Mao , X. Li , C. Wang , ACS Appl. Mater. Interfaces 2018, 10, 14767–14776; 29620854 10.1021/acsami.8b02425

[33c] S.-B. Son , T. Gao , S. P. Harvey , K. X. Steirer , A. Stokes , A. Norman , C. Wang , A. Cresce , K. Xu , C. Ban , Nat. Chem. 2018, 10, 532–539; 29610460 10.1038/s41557-018-0019-6

[33d] B. Li , R. Masse , C. Liu , Y. Hu , W. Li , G. Zhang , G. Cao , Energy Storage Mater. 2019, 22, 96–104;

[33e] J. Zhang , X. Guan , R. Lv , D. Wang , P. Liu , J. Luo , Energy Storage Mater. 2020, 26, 408–413;

[33f] K. Tang , A. Du , S. Dong , Z. Cui , X. Liu , C. Lu , J. Zhao , X. Zhou , G. Cui , Adv. Mater. 2020, 32, 1904987; 10.1002/adma.201904987 31850607

[33g] Z. Liang , C. Ban , Angew. Chem. Int. Ed. 2021, 60, 11036–11047. 10.1002/anie.202006472 32691897

[34a] G. A. Giffin , A. Moretti , S. Jeong , S. Passerini , J. Phys. Chem. C 2014, 118, 9966–9973;

[34b] T. Watkins , D. A. Buttry , J. Phys. Chem. B 2015, 119, 7003–7014. 25985170 10.1021/acs.jpcb.5b00339

[35] O. Borodin , G. A. Giffin , A. Moretti , J. B. Haskins , J. W. Lawson , W. A. Henderson , S. Passerini , J. Phys. Chem. C 2018, 122, 20108–20121.

[36] M. Balabajew , H. Reinhardt , N. Bock , M. Duchardt , S. Kachel , N. Hampp , B. Roling , Electrochim. Acta 2016, 211, 679–688.

[37] P. Schmitz , M. Kolek , D. Diddens , M. C. Stan , K. Jalkanen , M. Winter , P. Bieker , Phys. Chem. Chem. Phys. 2017, 19, 19178–19187. 28702548 10.1039/c7cp03716c

[38a] L. J. Hardwick , M. Hahn , P. Ruch , M. Holzapfel , W. Scheifele , H. Buqa , F. Krumeich , P. Novák , R. Kötz , Electrochim. Acta 2006, 52, 675–680;

[38b] L. J. Hardwick , P. W. Ruch , M. Hahn , W. Scheifele , R. Kötz , P. Novák , J. Phys. Chem. Solids 2008, 69, 1232–1237;

[38c] L. Zhang , J. Li , Y. Huang , D. Zhu , H. Wang , Langmuir 2019, 35, 3972–3979; 30811939 10.1021/acs.langmuir.9b00262

[38d] J. Gao , S. Tian , L. Qi , H. Wang , Electrochim. Acta 2015, 176, 22–27;

[38e] J. Gao , S. Tian , L. Qi , M. Yoshio , H. Wang , J. Power Sources 2015, 297, 121–126.

[39] M. A. Pimenta , G. Dresselhaus , M. S. Dresselhaus , L. G. Cançado , A. Jorio , R. Saito , Phys. Chem. Chem. Phys. 2007, 9, 1276–1290. 17347700 10.1039/b613962k

[40a] W. Märkle , N. Tran , D. Goers , M. E. Spahr , P. Novák , Carbon 2009, 47, 2727–2732;

[40b] L. J. Hardwick , H. Buqa , M. Holzapfel , W. Scheifele , F. Krumeich , P. Novák , Electrochim. Acta 2007, 52, 4884–4891.

[41] A. Heckmann , P. Meister , L.-Y. Kuo , M. Winter , P. Kaghazchi , T. Placke , Electrochim. Acta 2018, 284, 669–680.

[42] V. Küpers , M. Kolek , P. Bieker , M. Winter , G. Brunklaus , Phys. Chem. Chem. Phys. 2019, 21, 26084–26094. 31746873 10.1039/c9cp05334d

[43a] B. T. Yu , W. H. Qiu , F. S. Li , L. Cheng , J. Power Sources 2006, 158, 1373–1378;

[43b] E. G. Leggesse , J.-C. Jiang , J. Phys. Chem. A 2012, 116, 11025–11033; 23078373 10.1021/jp3081996

[43c] L. Madec , R. Petibon , K. Tasaki , J. Xia , J. P. Sun , I. G. Hill , J. R. Dahn , Phys. Chem. Chem. Phys. 2015, 17, 27062–27076; 26412322 10.1039/c5cp04221f

[43d] H. Ota , T. Akai , H. Namita , S. Yamaguchi , M. Nomura , J. Power Sources 2003, 119–121, 567–571.

[44a] D. R. Gallus , R. Wagner , S. Wiemers-Meyer , M. Winter , I. Cekic-Laskovic , Electrochim. Acta 2015, 184, 410–416;

[44b] N. von Aspern , D. Diddens , T. Kobayashi , M. Börner , O. Stubbmann-Kazakova , V. Kozel , G.-V. Röschenthaler , J. Smiatek , M. Winter , I. Cekic-Laskovic , ACS Appl. Mater. Interfaces 2019, 11, 16605–16618. 30965002 10.1021/acsami.9b03359

[45] S. Pohlmann , B. Lobato , T. A. Centeno , A. Balducci , Phys. Chem. Chem. Phys. 2013, 15, 17287–17294. 24019082 10.1039/c3cp52909f

[46] B. Heidrich , A. Heckmann , K. Beltrop , M. Winter , T. Placke , Energy Storage Mater. 2019, 21, 414–426.

[47] R. Nölle , K. Beltrop , F. Holtstiege , J. Kasnatscheew , T. Placke , M. Winter , Mater. Today 2020, 32, 131–146.

[48a] L. Doucey , M. Revault , A. Lautié , A. Chaussé , R. Messina , Electrochim. Acta 1999, 44, 2371–2377;

[48b] O. Borodin , M. Olguin , P. Ganesh , P. R. C. Kent , J. L. Allen , W. A. Henderson , Phys. Chem. Chem. Phys. 2016, 18, 164–175; 26601903 10.1039/c5cp05121e

[48c] Y. Kameda , S. Saito , Y. Umebayashi , K. Fujii , Y. Amo , T. Usuki , J. Mol. Liq. 2016, 217, 17–22;

[48d] O. Borodin , G. D. Smith , J. Phys. Chem. B 2009, 113, 1763–1776; 19146427 10.1021/jp809614h

[48e] A. V. Cresce , S. M. Russell , O. Borodin , J. A. Allen , M. A. Schroeder , M. Dai , J. Peng , M. P. Gobet , S. G. Greenbaum , R. E. Rogers , K. Xu , Phys. Chem. Chem. Phys. 2017, 19, 574–586. 10.1039/c6cp07215a 27918030

[49] M. G. Giorgini , K. Futamatagawa , H. Torii , M. Musso , S. Cerini , J. Phys. Chem. Lett. 2015, 6, 3296–3302.

[50] E. Krämer , S. Passerini , M. Winter , ECS Electrochem. Lett. 2012, 1, C9–C11.

[51] J. Becking , A. Gröbmeyer , M. Kolek , U. Rodehorst , S. Schulze , M. Winter , P. Bieker , M. C. Stan , Adv. Mater. Interfaces 2017, 4, 1700166.

