# Supplementary material for: Opportunities and Limitations of Ionic Liquid‐ and Organic Carbonate Solvent‐Based Electrolytes for Mg‐Ion‐Based Dual‐Ion Batteries
Source: ChemSusChem. 2021 Sep 2;14(20):4480–98. doi: 10.1002/cssc.202101227 (PMC8596887; doi:10.1002/cssc.202101227)
Supplement: Supplementary file 1 — Supporting Information [file CSSC-14-4480-s001.pdf]

ChemSusChem

Supporting Information

Opportunities and Limitations of Ionic Liquid- and Organic Carbonate Solvent-Based Electrolytes for Mg-Ion-Based Dual-Ion Batteries

Verena Küpers, Jan Frederik Dohmann, Peter Bieker, Martin Winter,* Tobias Placke,* and Martin Kolek*© 2021 The Authors. ChemSusChem published by Wiley-VCH GmbH. This is an open access article under the terms of the Creative Commons Attribution License, which permits use, distribution and reproduction in any medium, provided the original work is properly cited.

Supporting Information

Opportunities and Limitations of Ionic-Liquid- and Organic Carbonate Solvent-Based Electrolytes for Mg-Ion-Based Dual-Ion Batteries

Verena Küpers[a], Jan Frederik Dohmann[a], Peter Bieker[a, b], Martin Winter*[a, b], Tobias Placke*[a] and Martin Kolek*[a]

[a] V. Küpers, J. F. Dohmann, Dr. P. Bieker, Prof. Dr. M. Winter, Dr. T. Placke, Dr. M. Kolek
MEET Battery Research Center, Institute of Physical Chemistry
University of Münster
Corrensstraße 46, 48149 Münster, Germany
Email: m.winter@fz-juelich.de
tobias.placke@uni-muenster.de
martin.kolek@uni-muenster.de

[b] Dr. P. Bieker, Prof. Dr. M. Winter
Helmholtz Institute Münster (HI MS), IEK-12
Forschungszentrum Jülich GmbH
Corrensstrasse 46, 48149 Münster, Germany

Table S1. List of abbreviations.

AC	Activated carbon
AGG	Aggregate
cc-	*cis-cis* conformer of
CE	Counter electrode
C_{Eff}	Coulombic efficiency
CEI	Cathode electrolyte interphase
CIP	Contact ion pair
CMC	Sodium carboxymethylcellulose
ct-	*cis-trans* conformer of
DEC	Diethyl carbonate
DIB	Dual-ion battery
DMC	Dimethyl carbonate
EG	Expanded graphite
ES	Ethylene sulfide
Fc	Ferrocene
I	Areal intensity
Li	Lithium
LIB	Lithium ion battery
M	Mol per liter solvent
Mg	Magnesium
OCP	Open-circuit potential
Pt	Platinum
PTFE	Polytetrafluoroethylene
Pyr_{14}TFSI or Pyr (in electrolyte abbreviations)	1-butyl-1-methylpyrrolidinium bis(trifluoromethanesulfonyl)imide
QRE	Quasi-reference electrode
RE	Reference electrode
RMB	Rechargeable magnesium battery
SDC	Specific discharge capacity
SEI	Solid electrolyte interphase
SHE	Standard hydrogen electrode
Si	Silicon
SSIP	Solvent separated ion pair
$TFSI^-$	Bis(trifluoromethanesulfonyl)imide anion
WE	Working electrode
XRD	X-ray diffraction

Table S2a. Description of different cycling procedures used within this work. Before the first step, an open circuit potential (OCP) step of 10 h was applied for every cycling procedure. The electrolytes used in cells cycled with this procedure are indicated in brackets. The device used for all cells with this procedure is enclosed in square brackets.

Procedure 1:
Constant current cycling (Mg-Pyr, Li-Pyr, Mg-Pyr+ES, Mg-DMC, Mg-DEC) [MACCOR]

Step		Specific current / mA g^{-1}	Cut-off potential *vs*. Li|Li^+ / V	Repetition
1	Charge	10	5.0 (Mg-ion-based)<br>4.9 (Li-ion-based)	1
	Discharge	10	3.4	
2	Charge	100	5.0 (Mg-ion-based)<br>4.9 (Li-ion-based)	300
	Discharge	100	3.4	

Procedure 2:
Constant current cycling at high cut-offs (Mg-Pyr) [VMP3]

Step		Specific current / mA g^{-1}	Cut-off potential *vs*. Li|Li^+ / V	Repetition
1	Charge	10	5.0	1
	Discharge	10	3.4	
2	Charge	100	5.3	300
	Discharge	100	3.4	

Procedure 3:
Constant current cycling at high cut-offs and currents (Mg-Pyr+ES) [VMP3]

Step		Specific current / mA g^{-1}	Cut-off potential *vs*. Li|Li^+ / V	Repetition
1	Charge	10	5.0	1
	Discharge	10	3.4	
2	Charge	300	5.3	400
	Discharge	300	3.4	

Table S2b. Description of different cycling procedures used within this work. Before the first step, an OCP step of 10 h was applied for every cycling procedure. The electrolytes used in cells cycled with this procedure are indicated in brackets. The device used for all cells with this procedure is enclosed in square brackets.

<table>
<tr><td colspan="5">Procedure 4:
Constant current cycling at varying cut-offs potentials
(Mg-Pyr, Li-Pyr, Mg-Pyr+ES, Mg-DMC, Mg-DEC) [VMP3]
For an easier comparison of the cell performance depending on the cut-off potential, the SDCs and C_{Eff} of the 10th cycle of step 2-12 are shown in Figure 4a, 7a and 10b, and Table S6.</td></tr>
<tr><td colspan="2">Step</td><td>Specific current / mA g^{-1}</td><td>Cut-off potential vs. Li|Li^+ / V</td><td>Repetition</td></tr>
<tr><td rowspan="2">1</td><td>Charge</td><td>10</td><td>5.0 V (Mg-ion-based)
4.9 V (Li-ion-based)</td><td rowspan="2">1</td></tr>
<tr><td>Discharge</td><td>10</td><td>3.4</td></tr>
<tr><td rowspan="2">2</td><td>Charge</td><td>100</td><td>4.5</td><td rowspan="2">10</td></tr>
<tr><td>Discharge</td><td>100</td><td>3.4</td></tr>
<tr><td rowspan="2">3</td><td>Charge</td><td>100</td><td>4.6</td><td rowspan="2">10</td></tr>
<tr><td>Discharge</td><td>100</td><td>3.4</td></tr>
<tr><td rowspan="2">4</td><td>Charge</td><td>100</td><td>4.7</td><td rowspan="2">10</td></tr>
<tr><td>Discharge</td><td>100</td><td>3.4</td></tr>
<tr><td rowspan="2">5</td><td>Charge</td><td>100</td><td>4.8</td><td rowspan="2">10</td></tr>
<tr><td>Discharge</td><td>100</td><td>3.4</td></tr>
<tr><td rowspan="2">6</td><td>Charge</td><td>100</td><td>4.9</td><td rowspan="2">10</td></tr>
<tr><td>Discharge</td><td>100</td><td>3.4</td></tr>
<tr><td rowspan="2">7</td><td>Charge</td><td>100</td><td>5.0</td><td rowspan="2">10</td></tr>
<tr><td>Discharge</td><td>100</td><td>3.4</td></tr>
<tr><td rowspan="2">8</td><td>Charge</td><td>100</td><td>5.1</td><td rowspan="2">10</td></tr>
<tr><td>Discharge</td><td>100</td><td>3.4</td></tr>
<tr><td rowspan="2">9</td><td>Charge</td><td>100</td><td>5.2</td><td rowspan="2">10</td></tr>
<tr><td>Discharge</td><td>100</td><td>3.4</td></tr>
<tr><td rowspan="2">10</td><td>Charge</td><td>100</td><td>5.3</td><td rowspan="2">10</td></tr>
<tr><td>Discharge</td><td>100</td><td>3.4</td></tr>
<tr><td rowspan="2">11</td><td>Charge</td><td>100</td><td>5.4</td><td rowspan="2">10</td></tr>
<tr><td>Discharge</td><td>100</td><td>3.4</td></tr>
<tr><td rowspan="2">12</td><td>Charge</td><td>100</td><td>5.5</td><td rowspan="2">10</td></tr>
<tr><td>Discharge</td><td>100</td><td>3.4</td></tr>
</table>

Table S2c. Description of different cycling procedures used within this work. Before the first step, an OCP step of 10 h was applied for every cycling procedure. The electrolytes used in cells cycled with this procedure are indicated in brackets. The device used for all cells with this procedure is enclosed in square brackets.

Procedure 5:
Constant current cycling with varying specific currents
(Mg-Pyr, Li-Pyr, Mg-Pyr+ES, Mg-DMC, Mg-DEC) [MACCOR]
For an easier comparison of the cell performance depending on the specific current, the SDCs and C_{Eff} of the 5th cycle of step 3-11 are shown in Figure 4b, 7b and 10c, and Table S7.

Step		Specific current / mA g^{-1}	Cut-off potential *vs*. Li|Li^+ / V	Repetition
1	Charge	10	4.9/5.0 (Li-/Mg-ion-based)	1
	Discharge	10	3.4	
2	Charge	100	4.9/5.0 (Li-/Mg-ion-based)	30
	Discharge	100	3.4	
3	Charge	10	4.9/5.0 (Li-/Mg-ion-based)	5
	Discharge	10	3.4	
4	Charge	20	4.9/5.0 (Li-/Mg-ion-based)	5
	Discharge	20	3.4	
5	Charge	50	4.9/5.0 (Li-/Mg-ion-based)	5
	Discharge	50	3.4	
6	Charge	100	4.9/5.0 (Li-/Mg-ion-based)	5
	Discharge	100	3.4	
7	Charge	200	4.9/5.0 (Li-/Mg-ion-based)	5
	Discharge	200	3.4	
8	Charge	300	4.9/5.0 (Li-/Mg-ion-based)	5
	Discharge	300	3.4	
9	Charge	500	4.9/5.0 (Li-/Mg-ion-based)	5
	Discharge	500	3.4	
10	Charge	1000	4.9/5.0 (Li-/Mg-ion-based)	5
	Discharge	1000	3.4	
11	Charge	2500	4.9/5.0 (Li-/Mg-ion-based)	5
	Discharge	2500	3.4	
12	Charge	5000	4.9/5.0 (Li-/Mg-ion-based)	5
	Discharge	5000	3.4	
13	Charge	10000	4.9/5.0 (Li-/Mg-ion-based)	5
	Discharge	10000	3.4	
14	Charge	100	4.9/5.0 (Li-/Mg-ion-based)	80
	Discharge	100	3.4	

Table S2d. Description of different cycling procedures used within this work. Before the first step, an OCP step of 10 h was applied for every cycling procedure. The electrolytes used in cells cycled with this procedure are indicated in brackets. The device used for all cells with this procedure is enclosed in square brackets.

**Procedure 6:**<br>Constant current cycling prior to XRD measurements<br>(Mg-Pyr, Li-Pyr, Mg-Pyr+ES, Mg-DMC, Mg-DEC) [MACCOR]				
Step		Specific current / mA g^{-1}	Cut-off potential *vs.* Li|Li$^+$ / V	Repetition
1	Charge	10	4.9/5.0 (Li-/Mg-ion-based)	3
	Discharge	10	3.4	
2	Charge	10	4.9/5.0 (Li-/Mg-ion-based)	1

Table S3. Specific discharge capacities (SDC) and Coulombic efficiencies (C_{Eff}) of selected cycles of graphite ‖ AC cells with selected electrolytes at 100 mA g^{-1} (1st cycle: 10 mA g^{-1}) with cut-off potentials of 3.4 V and 4.9 V (Li-ion-based) respectively 5.0 V (Mg-ion-based) *vs*. Li|Li^+.

	**Li-Pyr**		**Mg-Pyr (20 °C)**	
	SDC / mAh g^{-1}	C_{Eff} / %	SDC / mAh g^{-1}	C_{Eff} / %
1st cycle	32 ± 1	64 ± 2	29 ± 1	74 ± 1
50th cycle	37 ± 4	99.1 ± 0.3	34 ± 4	99.5 ± 0.1
300th cycle	34 ± 3	99.5 ± 0.2	35 ± 3	99.8 ± 0.1

	**Mg-Pyr (upper cut-off potential: 5.3 V *vs.* Li|Li⁺)**		**Mg-Pyr (60 °C)***	
	SDC / mAh g^{-1}	C_{Eff} / %	SDC / mAh g^{-1}	C_{Eff} / %
1st cycle	33 ± 2	72.9 ± 0.7	52 ± 1	-
50th cycle	86 ± 5	97.2 ± 0.9	56 ± 2	95.0 ± 0.9
300th cycle	55 ± 11	97.7 ± 0.5	47 ± 2	95.8 ± 0.5

	**Mg-Pyr+ES**		**Mg-Pyr+ES, (at 300 mAh g⁻¹, upper cut-off potential: 5.3 V *vs.* Li|Li⁺)**	
	SDC / mAh g^{-1}	C_{Eff} / %	SDC / mAh g^{-1}	C_{Eff} / %
1st cycle	45 ± 2	73 ± 2	40.9 ± 0.7	79.2 ± 0.8
50th cycle	53 ± 4	99.1 ± 0.3	93 ± 2	99.1 ± 0.2
300th cycle	50 ± 3	99.4 ± 0.2	84 ± 5	98.9 ± 0.2
400th cycle			82 ± 4	98.8 ± 0.2

	**Mg-DMC**		**Mg-DEC**	
	SDC / mAh g^{-1}	C_{Eff} / %	SDC / mAh g^{-1}	C_{Eff} / %
1st cycle	46 ± 3	73.1 ± 0.7	45.9 ± 0.9	76 ± 2
50th cycle	39 ± 3	99.5 ± 0.2	39 ± 5	99.57 ± 0.01
300th cycle	41 ± 4	99.3 ± 0.7	41 ± 5	99.75 ± 0.02

* At 60 °C, strong parasitic side reactions could be observed at 10 mA g^{-1} in the first cycle below 5.0 V *vs*. Li|Li^+, why the cut-off potential of 5.0 V *vs*. Li|Li^+ was not reached, before the time determination (10 hours, 100 mAh g^{-1}) was reached.

Table S4. $TFSI^{-}$ intercalation onset potentials (threshold: 10 mAh g^{-1} V^{-1}) in V *vs*. Li|Li^{+} of selected cycles of graphite ‖ AC cells with selected electrolytes at 100 mA g^{-1} (1st cycle: 10 mA g^{-1}) with cut-off potentials of 3.4 V and 4.9 V (Li-ion-based) respectively 5.0 V (Mg-ion-based) *vs*. Li|Li^{+}.

	**Li-Pyr**	**Mg-Pyr (20 °C)**	**Mg-Pyr (60 °C)**
1st cycle	4.61 ± 0.01	4.82 ± 0.01	4.54 ± 0.02
50th cycle	4.39 ± 0.01	4.48 ± 0.01	4.43 ± 0.03
100th cycle	4.38 ± 0.02	4.47 ± 0.02	4.40 ± 0.04
300th cycle	4.38 ± 0.02	4.45 ± 0.02	4.17 ± 0.10*

	**Mg-Pyr+ES**	**Mg-DMC**	**Mg-DEC**
1st cycle	4.64 ± 0.02	4.61 ± 0.01	4.64 ± 0.01
50th cycle	4.44 ± 0.03	4.53 ± 0.01	4.53 ± 0.02
100th cycle	4.44 ± 0.03	4.52 ± 0.02	4.52 ± 0.02
300th cycle	4.45 ± 0.03	4.51 ± 0.01	4.50 ± 0.02

* The lower onset potential is based on low capacities at low potentials. The main intercalation (threshold of 20 mAh g^{-1}) starts at 4.47 ± 0.03 V *vs*. Li|Li^{+} (at 20 °C: 4.45 ± 0.02 V *vs*. Li|Li^{+}).

Table S5. $d_{(00n+2)}/ d_{(00n+1)}$ ratio, dominant stage and specific charge and discharge capacities at selected potentials of graphite electrodes after three full cycles between 5.0 respectively 4.9 V *vs*. Li|Li$^+$ for Li-Pyr and 3.4 V *vs*. Li|Li$^+$ at 10 mA g^{-1} cycled in graphite ∥ AC Swagelok-type cells (three-electrode configuration; RE/QRE: Li metal) with Mg-Pyr and Li-Pyr electrolytes.

**Mg-Pyr**	Charge				Discharge			
**Cut-off potential / V *vs*. Li|Li$^+$**	4.5	4.6	4.8	5	4.5	4.4	4.2	3.4
$\frac{d_{(00n+2)}}{d_{(00n+1)}}$	-	1.16	1.24	1.29	1.21	1.13	-	-
**Dominant stage (*n*)**	-	5	3	2, 3	4	6	-	-
**Specific charge capacity / mAh g^{-1}**	5	13	29	42				
**Specific discharge capacity / mAh g^{-1}**					14	26	36	36

**Li-Pyr**	Charge				Discharge			
**Cut-off potential / V *vs*. Li|Li$^+$**	4.4	4.5	4.7	4.9	4.4	4.3	4.1	3.4
$\frac{d_{(00n+2)}}{d_{(00n+1)}}$	-	1.19	1.26	1.29	1.24	1.17	1.14	-
**Dominant stage (*n*)**	-	5	3	2, 3	3	5	6	-
**Specific charge capacity / mAh g^{-1}**	1	14	31	44				
**Specific discharge capacity / mAh g^{-1}**					13	20	23	39

**Mg-Pyr+ES**	Charge				Discharge			
**Cut-off potential / V *vs*. Li|Li$^+$**	4.5	4.6	4.8	5	4.5	4.4	4.2	3.4
$\frac{d_{(00n+2)}}{d_{(00n+1)}}$	-	1.12	1.25	1.33	1.14		-	-
**Dominant stage (*n*)**	-	6	3	2	6		-	-
**Specific charge capacity / mAh g^{-1}**	4	16	44	55				
**Specific discharge capacity / mAh g^{-1}**					22		45	49

Table S6. Specific discharge capacities (SDC) and Coulombic efficiencies (C_{Eff}) of $TFSI^{-}$ intercalation into graphite of graphite ‖ AC cells (three-electrode configuration; RE/QRE: Li metal) with selected electrolytes at different upper cut-off potentials at 100 mA g^{-1} (pre-cycle cycle: 10 mA g^{-1} with cut-off potentials of 3.4 V and 4.9 V (Li-ion-based) respectively 5.0 V (Mg-ion-based) *vs*. Li|Li^{+}).

	**Li-Pyr**		**Mg-Pyr**	
**Potential / V *vs*. Li|Li^{+}**	**SDC / mAh g^{-1}**	**C_{Eff} / %**	**SDC / mAh g^{-1}**	**C_{Eff} / %**
4.5	1.6 ± 0.2	101.4 ± 0.3	0.5 ± 0.1	100.6 ± 0.2
4.6	12.3 ± 0.7	99.4 ± 0.2	2 ± 2	100.0 ± 0.2
4.7	18.0 ± 0.4	99.2 ± 0.2	10 ± 3	99.6 ± 0.1
4.8	21.7 ± 0.6	99.0 ± 0.3	14 ± 4	99.49 ± 0.08
4.9	25.5 ± 0.8	98.3 ± 0.5	21 ± 5	99.2 ± 0.1
5.0	30 ± 1	96.9 ± 0.9	30 ± 6	98.8 ± 0.1
5.1	36 ± 3	95 ± 2	42 ± 6	98.37 ± 0.05
5.2	44 ± 5	93 ± 2	55 ± 8	98.13 ± 0.09
5.3	56 ± 10	92 ± 2	78 ± 15	97.6 ± 0.5
5.4	66 ± 12	89 ± 6	100 ± 12	92 ± 9
5.5	65 ± 15	83 ± 9	104 ± 9	73 ± 11

	**Mg-Pyr+ES**	
**Potential / V *vs*. Li|Li^{+}**	**SDC / mAh g^{-1}**	**C_{Eff} / %**
4.5	1 ± 1	100.16 ± 0.04
4.6	8 ± 4	99.83 ± 0.03
4.7	23 ± 4	99.69 ± 0.02
4.8	37 ± 3	99.56 ± 0.04
4.9	42 ± 2	99.47 ± 0.05
5.0	54 ± 3	98.9 ± 0.1
5.1	76 ± 8	97.9 ± 0.5
5.2	97 ± 7	97 ± 2
5.3	105 ± 8	96 ± 2
5.4	107 ± 11	82 ± 12
5.5	96 ± 9	55 ± 10

	**Mg-DMC**		**Mg-DEC**	
**Potential / V *vs*. Li|Li^{+}**	**SDC / mAh g^{-1}**	**C_{Eff} / %**	**SDC / mAh g^{-1}**	**C_{Eff} / %**
4.5	1.1 ± 0.3	100.41 ± 0.05	1.0 ± 0.2	100.14 ± 0.01
4.6	2 ± 2	100.2 ± 0.2	1.4 ± 0.4	99.97 ± 0.01
4.7	6 ± 5	99.9 ± 0.2	9 ± 4	99.85 ± 0.04
4.8	14 ± 8	99.74 ± 0.09	20 ± 5	99.70 ± 0.03
4.9	22 ± 10	99.6 ± 0.2	31 ± 5	99.56 ± 0.04
5.0	30 ± 10	99.5 ± 0.3	40 ± 5	99.3 ± 0.1
5.1	43 ± 21	99 ± 1	55 ± 8	98.4 ± 0.5
5.2	61 ± 22	98 ± 2	76 ± 10	96.7 ± 0.8
5.3	75 ± 16	97 ± 2	92 ± 11	94 ± 2
5.4	84 ± 12	96 ± 3	90 ± 13	91 ± 3
5.5	85 ± 5	94 ± 4	Decomposition	

Table S7. Specific discharge capacity (SDC) and the corresponding Coulombic efficiencies (C_{Eff}) of $TFSI^-$ intercalation of graphite ‖ AC cells (three-electrode configuration; RE/QRE: Li metal) at different specific currents with selected electrolytes (pre-cycle at cycle: 10 mA g^{-1}) with cut-off potentials of 3.4 V and 4.9 V (Li-ion-based) respectively 5.0 V (Mg-ion-based) *vs*. $Li|Li^+$.

	**Li-Pyr**		**Mg-Pyr**	
**Specific current / (mA g^{-1})**	**SDC / (mAh g^{-1})**	**C_{Eff} / %**	**SDC / (mAh g^{-1})**	**C_{Eff} / %**
10	40 ± 6	89 ± 5	46 ± 3	94 ± 2
20	38 ± 6	94 ± 3	44 ± 3	97 ± 1
50	36 ± 6	97 ± 1	41 ± 3	98.5 ± 0.7
100	34 ± 6	98 ± 1	39 ± 3	99.2 ± 0.4
200	33 ± 6	99.1 ± 0.6	36 ± 3	99.6 ± 0.2
300	31 ± 5	99.4 ± 0.4	34 ± 3	99.8 ± 0.2
500	30 ± 5	99.7 ± 0.3	31 ± 3	99.93 ± 0.07
1000	25 ± 4	99.9 ± 0.2	25 ± 4	100.07 ± 0.05
2500	6 ± 3	100 ± 1	9 ± 6	100.7 ± 0.7

	**Mg-Pyr+ES**	
**Specific current / (mA g^{-1})**	**SDC / mAh g^{-1}**	**C_{Eff} / %**
10	58.8 ± 0.4	94 ± 1
20	58.6 ± 0.2	96 ± 1
50	58 ± 1	98 ± 1
100	56 ± 2	98.9 ± 0.9
200	54 ± 2	99.4 ± 0.6
300	51 ± 4	99.6 ± 0.5
500	48 ± 4	99.8 ± 0.3
1000	42 ± 6	99.9 ± 0.2
2500	20 ± 15	99.9 ± 0.1

	**Mg-DMC**		**Mg-DEC**	
**Specific current / (mA g^{-1})**	**SDC / mAh g^{-1}**	**C_{Eff} / %**	**SDC / mAh g^{-1}**	**C_{Eff} / %**
10	51 ± 9	95 ± 3	58 ± 8	92 ± 3
20	51 ± 10	97 ± 1	58 ± 9	96 ± 2
50	43 ± 4	99.3 ± 0.2	54 ± 9	98.3 ± 0.8
100	38 ± 4	99.8 ± 0.2	48 ± 8	99.4 ± 0.4
200	30 ± 5	100.0 ± 0.1	42 ± 8	99.8 ± 0.2
300	22 ± 5	100.1 ± 0.2	36 ± 10	99.9 ± 0.1
500	11 ± 3	100.8 ± 0.7	24 ± 10	100.0 ± 0.1
1000	1.0 ± 0.6	103.7 ± 0.3	5 ± 4	100.8 ± 0.6

Table S8. Calculated ratios of the area of the bands of 'free' $TFSI^-$ (743 ± 1 cm^{-1}) $A_{uncoord}$, the coordinated $TFSI^-$ at 746 ± 1 cm^{-1} A_{coord1} and at 752 ± 1 cm^{-1} A_{coord2} of the total obtained area calculated using a pseudo Voigt function.

	$\frac{A_{uncoord}}{A_{total}}$ / %	$\frac{A_{coord1}}{A_{total}}$ / %	$\frac{A_{coord2}}{A_{total}}$ / %
Mg-Pyr	69 ± 1	8 ± 1	24 ± 1
Mg-Pyr+ES	71 ± 1	10 ± 1	19 ± 1
Mg-Pyr+10ES	69 ± 6	17 ± 5	15 ± 1
Mg-Pyr+EC	69 ± 1	13 ± 1	18 ± 1

Table S9. Specific discharge capacities (SDC) and Coulombic efficiencies (C_{Eff}) of selected cycles of graphite ‖ AC cells (three-electrode configuration; QRE: Li metal) with Mg-Pyr at 100 mA g^{-1} using pre-cycled graphite and pristine or pre-cycled AC (three pre-cycles in graphite ‖ AC cells with Mg-Pyr+ES at 10 mA g^{-1}) with cut-off potentials of 3.4 V and 5.0 V *vs*. Li|Li^+. The first cycle corresponds to the first cycle after pre-cycling.

	**Pristine AC**		**Pre-cycled AC**	
	**SDC / mAh g^{-1}**	**C_{Eff} / %**	**SDC / mAh g^{-1}**	**C_{Eff} / %**
1st cycle	42.0 ± 0.2	96.07 ± 0.02	46 ± 3	97 ± 2
50th cycle	40 ± 3	99.3 ± 0.2	52 ± 4	99.3 ± 0.3
300th cycle	38 ± 3	99.7 ± 0.1	49 ± 3	99.6 ± 0.3

Table S10. $d_{(00n+2)}$/ $d_{(00n+1)}$ ratio and dominant stage of graphite electrodes after three full cycles between 5.0 V and 3.4 V *vs*. Li|Li^+ at 10 mA g^{-1} cycled in graphite ‖ AC Swagelok-type cells (three-electrode configuration; QRE: Li metal) with Mg-Pyr and Mg-Pyr+ES and one charging step to 5.0 V *vs*. Li|Li^+ at 10 mA g^{-1}, respectively 5.3 V *vs*. Li|Li^+ at 100 mA g^{-1}.*

	**Mg-Pyr**		**Mg-Pyr+ES**	
	**5.0 V *vs.* Li|Li^+**	**5.3 V *vs.* Li|Li^+**	**5.0 V *vs.* Li|Li^+**	**5.3 V *vs.* Li|Li^+**
$\frac{d_{(00n+2)}}{d_{(00n+1)}}$	1.29	1.50	1.33	1.51
Dominant stage (*n*)	2, 3	1	2	1

*Graphite ‖ AC cells cycled with 10 mA g^{-1} did not reach the cut-off potential of 5.3 V vs Li|Li^+ within 10 h, similar to the cells cycled at 60 °C, likely resulting from side reactions, why a current of 100 mA g^{-1} was used for high cut-off potentials.

Table S11. $TFSI^-$ intercalation onset potentials in V *vs*. Li|Li^+ of graphite ‖ AC cells (three-electrode configuration; QRE: Li metal) with selected electrolytes at selected specific currents with cut-off potentials of 3.4 V and 5.0 V *vs*. Li|Li^+.

**Specific current / mA g^{-1}**	**Mg-Pyr**	**Mg-Pyr+ES**
100	4.46 ± 0.02	4.45 ± 0.01
1000	4.61 ± 0.02	4.60 ± 0.04
2500	4.83 ± 0.07	4.79 ± 0.07

Table S12. Calculated ratios of the area of the bands of ‘free’ $TFSI^-$ (743 ± 1 cm^{-1}) $A_{uncoord}$, the coordinated $TFSI^-$ at 746 ± 1 cm^{-1} A_{coord1} and at 752 ± 1 cm^{-1} A_{coord2} of the total obtained area calculated using a pseudo Voigt function.

	$\frac{A_{uncoord}}{A_{total}}$ / %	$\frac{A_{coord1}}{A_{total}}$ / %	$\frac{A_{coord2}}{A_{total}}$ / %
Mg-DMC	32 ± 2	36 ± 2	32 ± 1
Mg-DEC	39 ± 10	24 ± 9	37 ± 2

Table S13. Raman bands of selected electrodes and references between 830 and 960 cm^{-1}, associated with the C-O stretching mode of carbonates.

**DMC**	**Mg-DMC**	**Association**
861 cm^{-1} (*w*)	862 cm^{-1} (*w*)	Free DMC (ct)
	882 cm^{-1}	Coordinated DMC (ct)
917 cm^{-1}	917 cm^{-1}	Free DMC (cc)
	945 cm^{-1}	Coordinated DMC (cc)

**DEC**	**Mg-DEC**	**Association**
854 cm^{-1} (*w*)		Free DEC (ct)
	857 cm^{-1}	Coordinated DEC (ct)
903 cm^{-1}	903 cm^{-1}	Free DEC (cc)
	913 cm^{-1}	Coordinated DEC (cc)

Table S14. $d_{(00n+2)}$/ $d_{(00n+1)}$ ratio and dominant stage of graphite electrodes after three cycles between 5.0 V and 3.4 V *vs*. Li|Li^+ at 10 mA g^{-1} cycled in graphite ‖ AC Swagelok-type cells (three-electrode configuration; QRE: Li metal) with Mg-DMC and Mg-DEC and one charging step to 5.0 V *vs*. Li|Li^+ at 10 mA g^{-1}.

	**Mg-DMC**	**Mg-DEC**
$\frac{d_{(00n+2)}}{d_{(00n+1)}}$	1.35	1.33
Dominant stage (*n*)	2	2

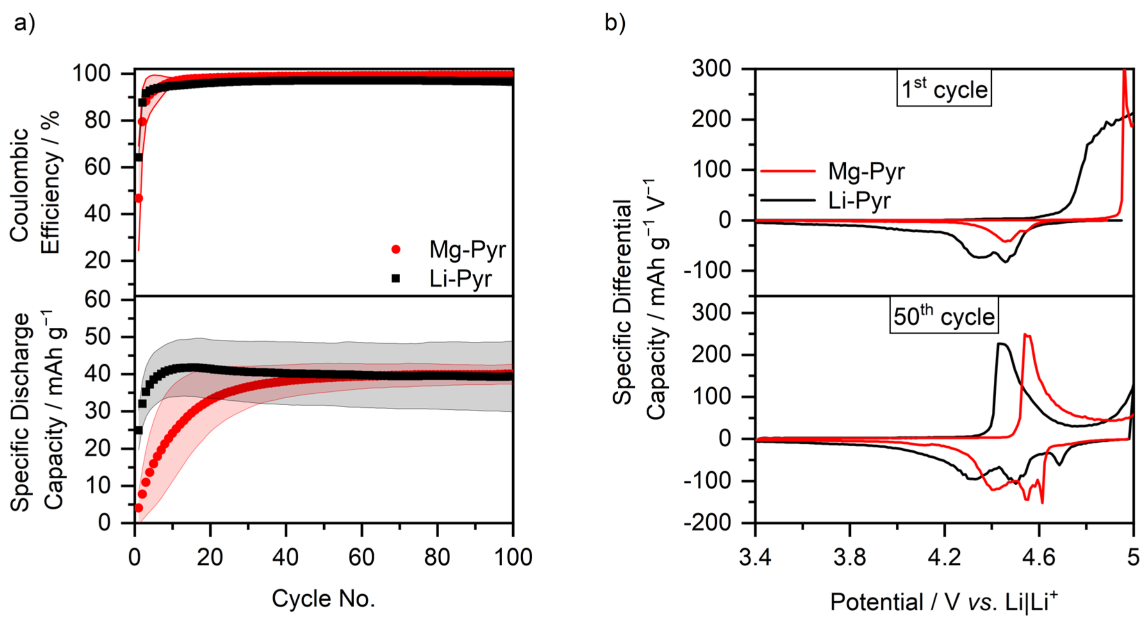

Figure S1. a) Coulombic efficiency and specific discharge capacity of graphite ‖ AC Swagelok-type cells (three-electrode configuration; RE/QRE: Li metal) with 0.5 M Mg(TFSI)$_2$ (red) and 1 M LiTFSI (black) in Pyr$_{14}$TFSI at 100 mA g^{-1} with cut-off potentials of 3.4 V and 5.0 V *vs*. Li|Li$^+$. The corresponding differential capacity *vs*. potential plots of b) the 1st and 50th cycle.

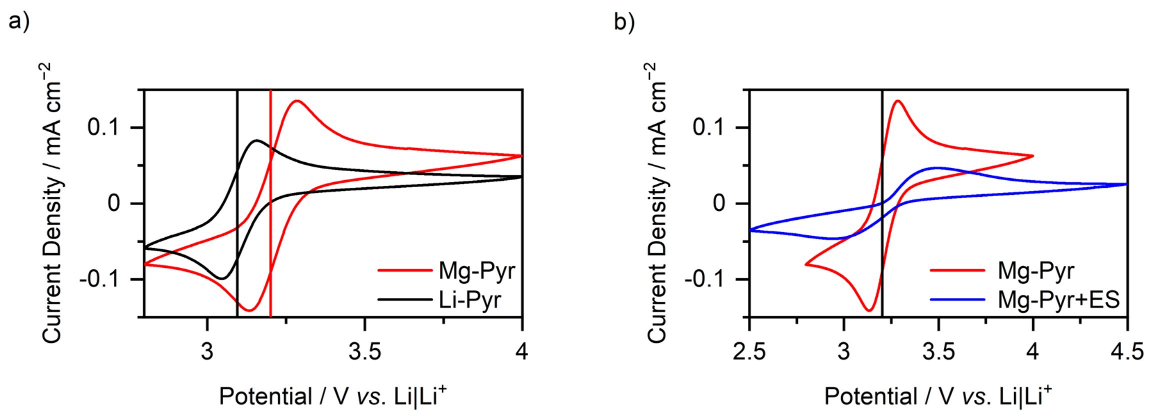

Figure S2. Cyclic voltammograms of Pt ‖ AC Swagelok-type cells (three-electrode configuration; RE/QRE: Li metal) with 0.05 M Fc in 0.5 M Mg(TFSI)$_2$ (red) and a) 1 M LiTFSI (black), respectively b) 0.5 M Mg(TFSI)$_2$ + 2 wt.% ES in Pyr$_{14}$TFSI at 5 mV s^{-1} with cut-off potentials of 2.8 and 4.0 V, respectively 2.5 and 4.5 V (Mg-Pyr+ES) *vs*. Li|Li$^+$.

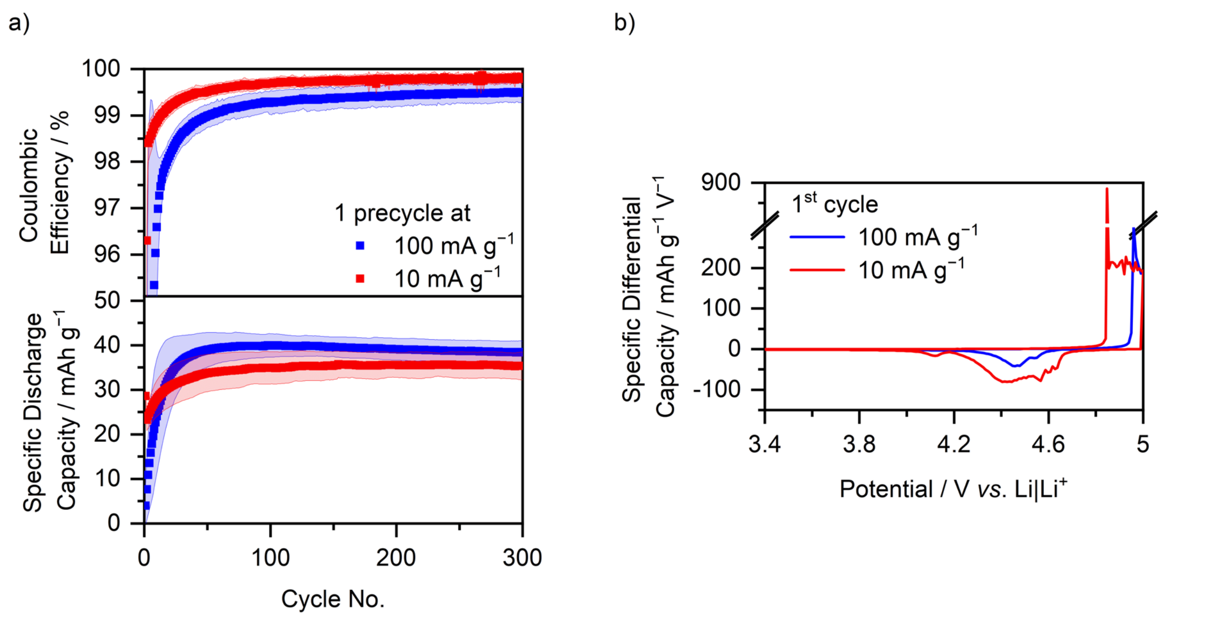

Figure S3. a) Coulombic efficiency and specific discharge capacity of graphite ‖ AC Swagelok-type cells (three-electrode configuration; RE/QRE: Li metal) with 0.5 M $Mg(TFSI)_2$ in $Pyr_{14}TFSI$ at 100 mA g^{-1} with cut-off potential of 3.4 V and 5.0 V *vs*. Li|Li^+ with 1 pre-cycle at 10 mA g^{-1} (red) or 100 mA g^{-1} (blue). b) The corresponding differential capacity *vs*. potential plots of the 1st cycle.

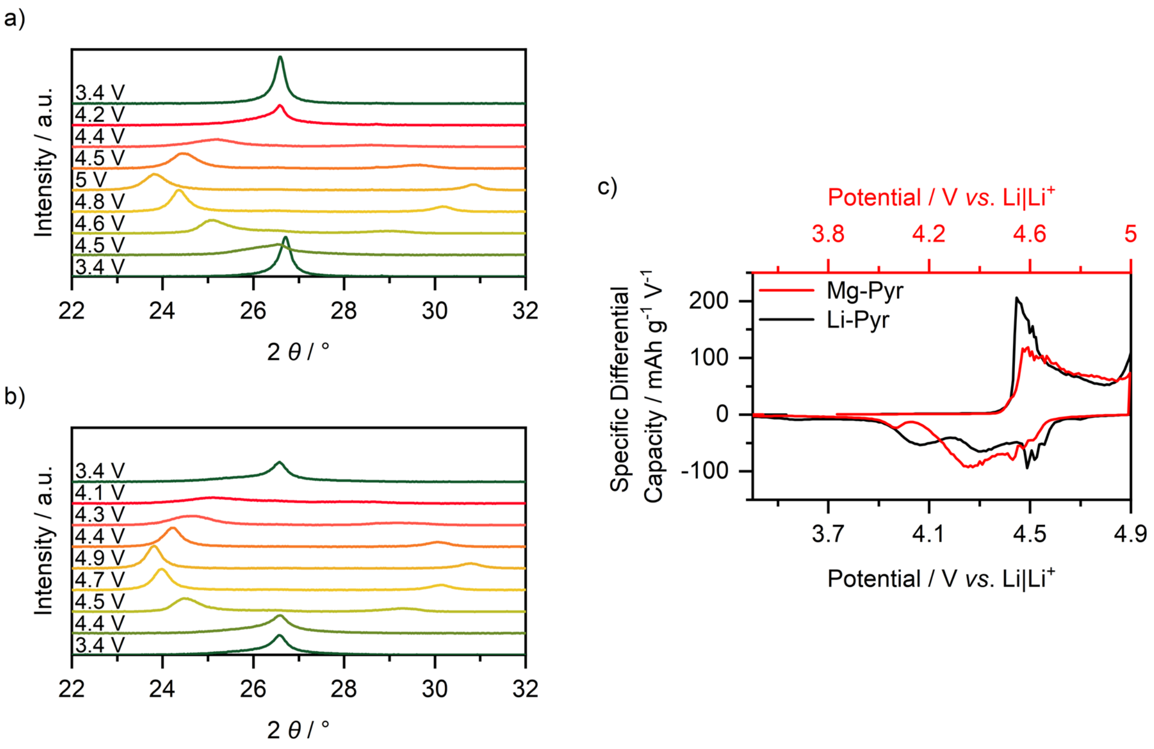

Figure S4. Ex situ XRD patterns of graphite composite electrodes at selected potentials (*vs*. Li|Li$^+$) in graphite ‖ AC Swagelok-type cells (three-electrode configuration; RE/QRE: Li metal) with a) 0.5 M Mg(TFSI)$_2$ and b) 1 M LiTFSI in Pyr$_{14}$TFSI after three cycles at 10 mA g^{-1} with cut-off potentials of 3.4 V and 4.9 V (Li-ion-based) respectively 5.0 V (Mg-ion-based) *vs*. Li|Li$^+$. c) The corresponding typical differential capacity *vs*. potential plots (4th cycle at 10 mA g^{-1}). For an easier comparison, the potential of the Mg-ion-based cell (red, upper x-axis) is shifted 0.1 V compared to the Li-ion-based cell (black, lower x-axis), according to variations in the potential of the Li metal QRE. The first XRD pattern (3.4 V, bottom) was recorded after the three pre-cycles.

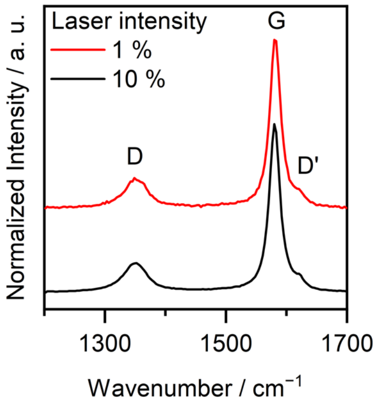

Figure S5. Normalized *ex situ* Raman spectra of a pristine graphite composite electrode (90% graphite) using a laser intensity of 1% (acquisition time: 5 x 30 s, red) and 10% (acquisition time: 60 x 5 s, black).

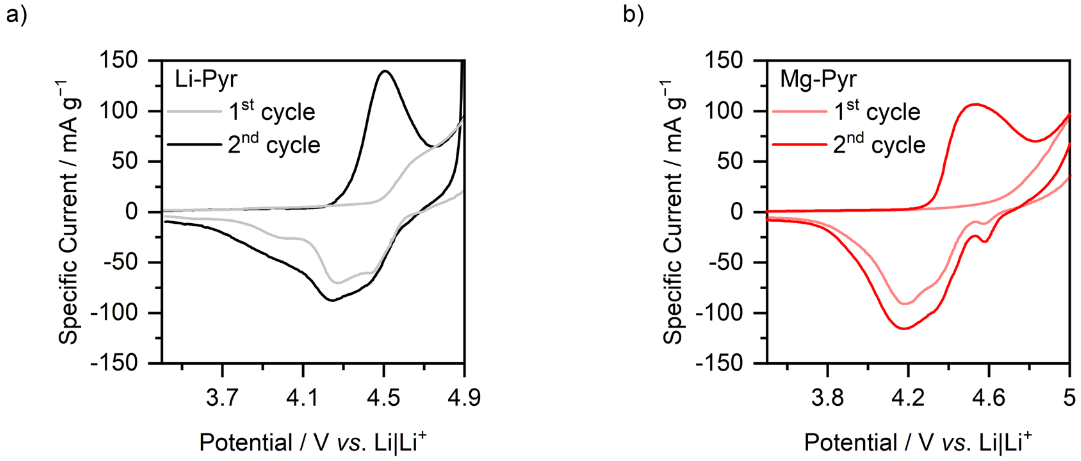

Figure S6. Cyclic voltammograms of the 1st and 2nd cycle of modified graphite ‖ AC pouch-type cells (three-electrode configuration; RE/QRE: Li metal, scan speed: 0.5 mV s^{-1}, cut-off potentials of 3.4 to 4.9 V (Li-ion-based), respectively 5.0 V *vs*. Li|Li$^+$ (Mg-ion-based)) of *in situ* Raman measurements (Figure 3) with a) Li-Pyr and b) Mg-Pyr.

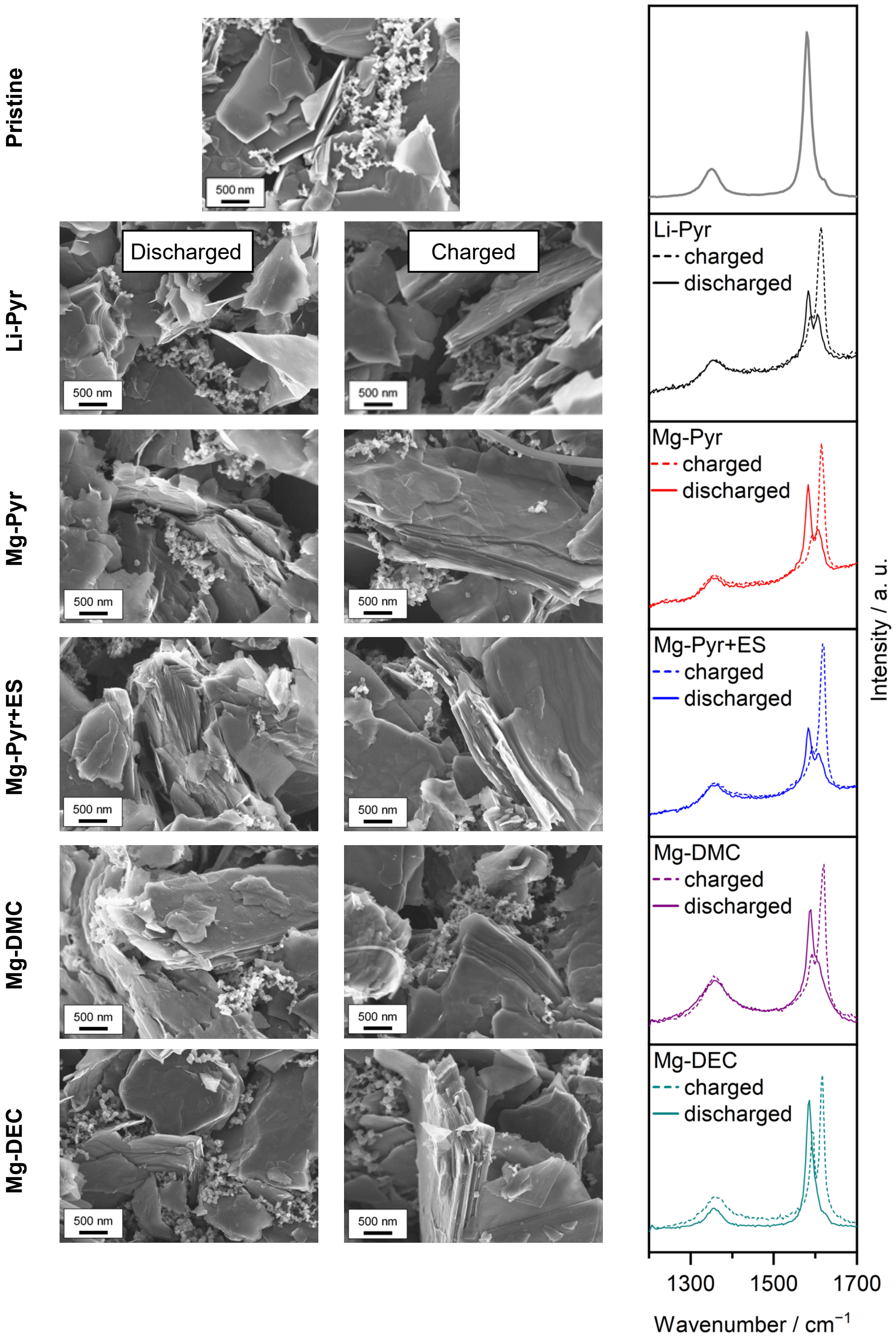

Figure S7. SEM images at a magnification of 25k of a pristine graphite electrode as well as washed electrodes after 100 cycles at 100 mA g^{-1} (1 pre-cycle: at 10 mA g^{-1}) with cut-off potentials of 3.4 V and 4.9 V (Li-ion-based) respectively 5.0 V (Mg-ion-based) *vs.* Li|Li^+ in graphite ‖ AC Swagelok-type cells (three-electrode configuration; RE/QRE: Li metal) with different electrolytes in the discharged (final potential of 3.4 V *vs.* Li|Li^+) and charged (an additional charging step to 4.9 V (Li-ion-based) respectively 5.0 V (Mg-ion-based)) state, and the corresponding *ex situ* Raman spectra between 1200 and 1700 cm^{-1} at a laser intensity of 10% (acquisition time: 60 x 5 s) of the non-washed electrodes (discharged: solid line; charged: dashed line).

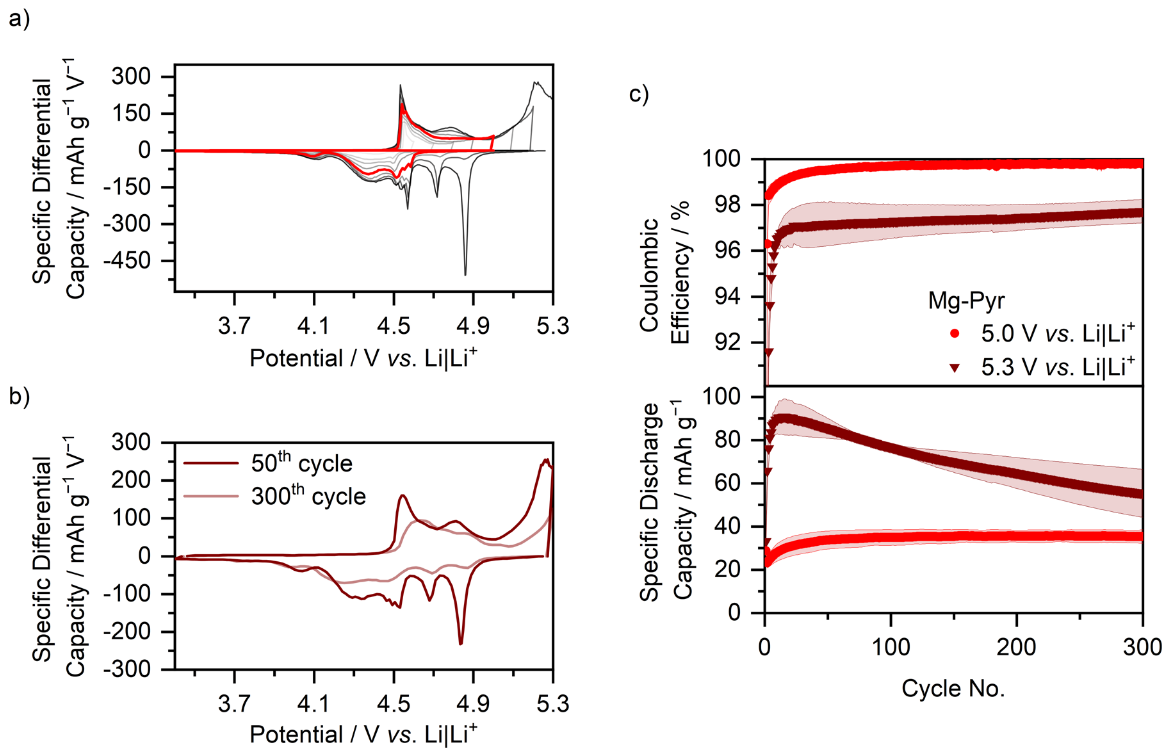

Figure S8. a) Differential capacity *vs*. potential plots of graphite ‖ AC Swagelok-type cell (three-electrode configuration; QRE: Li metal) with 0.5 M $Mg(TFSI)_2$ in $Pyr_{14}TFSI$ at 100 mA g^{-1} with various upper cut-off potentials (1 pre-cycle: 10 mA g^{-1}; 3.4 to 5.0 V *vs*. $Li|Li^+$). b) The differential capacity *vs*. potential plots of the 50th and 300th cycle and c) Coulombic efficiency and specific discharge capacity of graphite ‖ AC Swagelok-type cell with 0.5 M $Mg(TFSI)_2$ in $Pyr_{14}TFSI$ at 100 mA g^{-1} with cut-off potential of 3.4 V and 5.0 V (red) respectively 5.3 V (brown) *vs*. $Li|Li^+$ (1 precycle: 10 mA g^{-1}; 3.4 to 5.0 V *vs*. $Li|Li^+$).

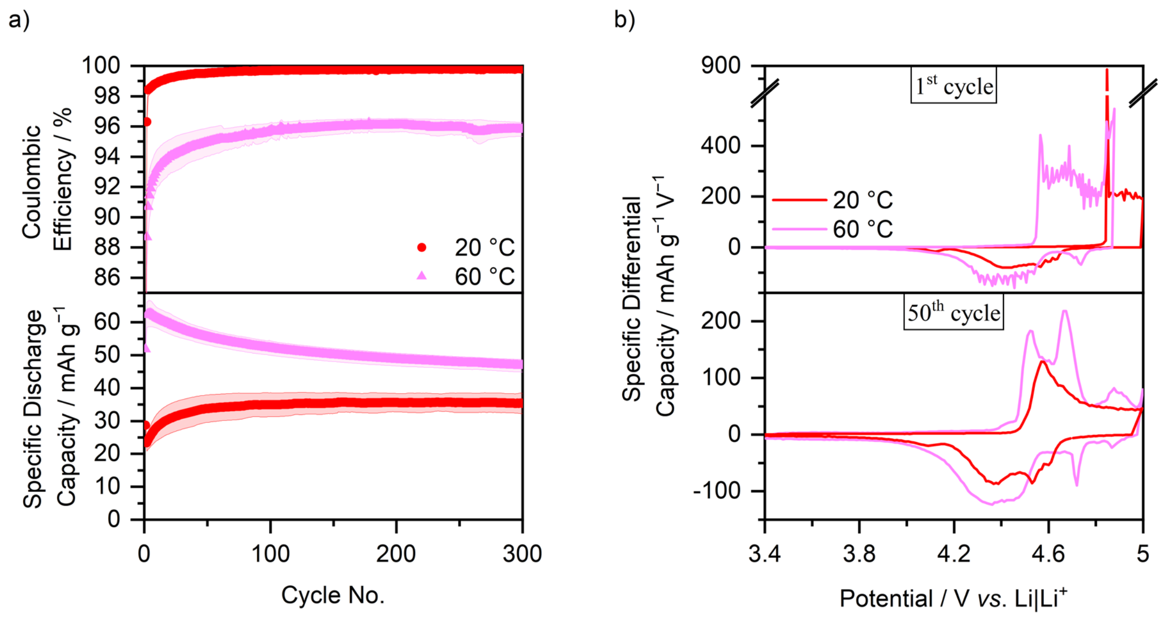

Figure S9. a) Coulombic efficiency and specific discharge capacity of graphite ‖ AC Swagelok-type cells (three-electrode configuration; QRE: Li metal) with 0.5 M $Mg(TFSI)_2$ in $Pyr_{14}TFSI$ at 20 °C (red) and 60 °C (pink) at 100 mA g^{-1} (1st cycle: 10 mA g^{-1}) with cut-off potentials of 3.4 V and 5.0 V *vs*. Li|Li^+. b) The corresponding differential capacity *vs*. potential plots of the 1st and 50th cycles. The Coulombic efficiency of the first cycle (Mg-Pyr: 74 ± 1%) is not shown.

At 60 °C, strong parasitic side reactions could be observed at 10 mA g^{-1} in the first cycle below 5.0 V *vs*. Li|Li^+, why the cut-off potential of 5.0 V *vs*. Li|Li^+ was not reached, before the time determination (10 hours, 100 mAh g^{-1}) was reached.

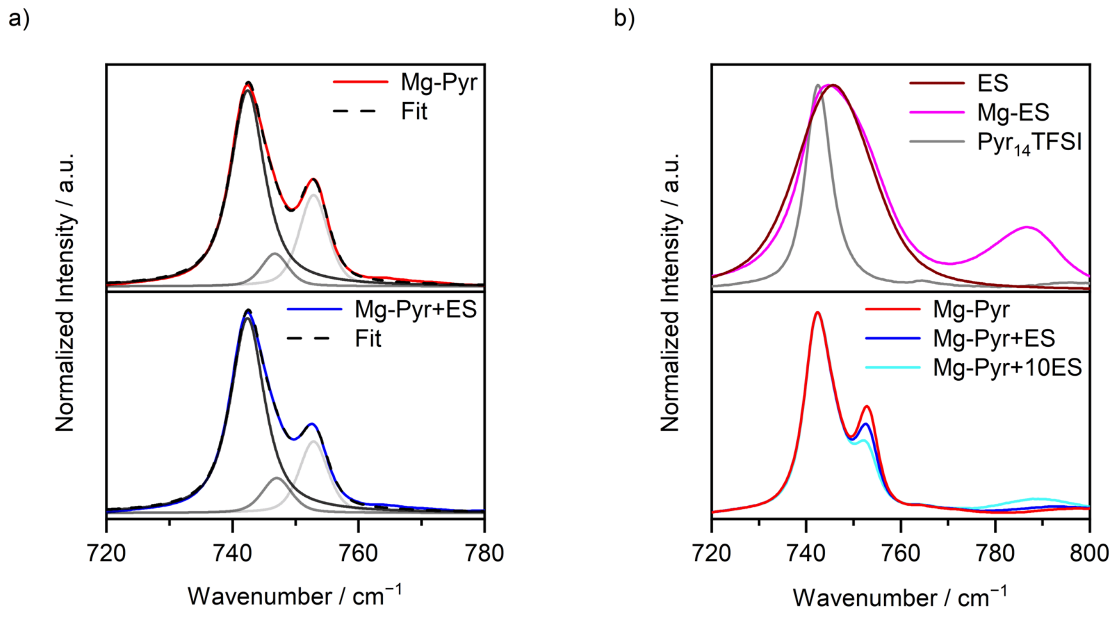

Figure S10. Raman spectra of 0.5 M $Mg(TFSI)_2$ (red) and 0.5 M $Mg(TFSI)_2$ + 2 wt.% ES (blue) between 720 and 780 cm^{-1} respectively 800 cm^{-1} a) including the fitted (pseudo Voigt) spectra and with b) pure ES (brown), 0.5 M $Mg(TFSI)_2$ in ES (pink), $Pyr_{14}TFSI$ (grey) and 0.5 M $Mg(TFSI)_2$ + approximately. 10 wt.% ES (bright blue) as references. The fitted spectra were normalized according to the maximal intensity of the band at ≈742 cm^{-1} of the experimental spectra.

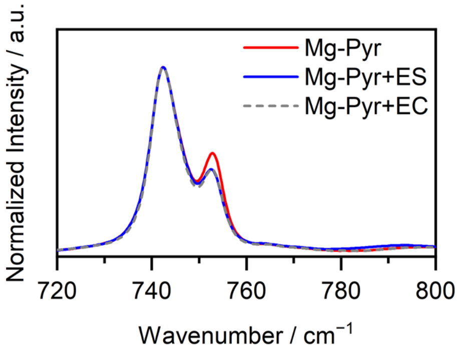

Figure S11. Raman spectra of 0.5 M $Mg(TFSI)_2$ (red) and 0.5 M $Mg(TFSI)_2$ + 2 wt.% ES (blue) and 0.5 M $Mg(TFSI)_2$ + 2 wt.% EC (grey) between 720 and 800 cm^{-1}.

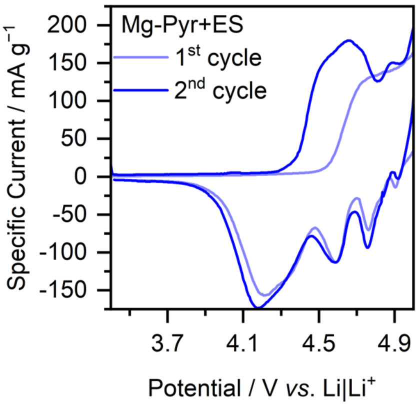

Figure S12. Cyclic voltammograms of the 1st and 2nd cycle of modified graphite ‖ AC pouch-type cells (three-electrode configuration; RE/QRE: Li metal, scan speed: 0.5 mV s^{-1}, cut-off potentials of 3.4 to 5.0 V *vs*. Li|Li^{+}) of *in situ* Raman measurements (Figure 6) with Mg-Pyr+ES.

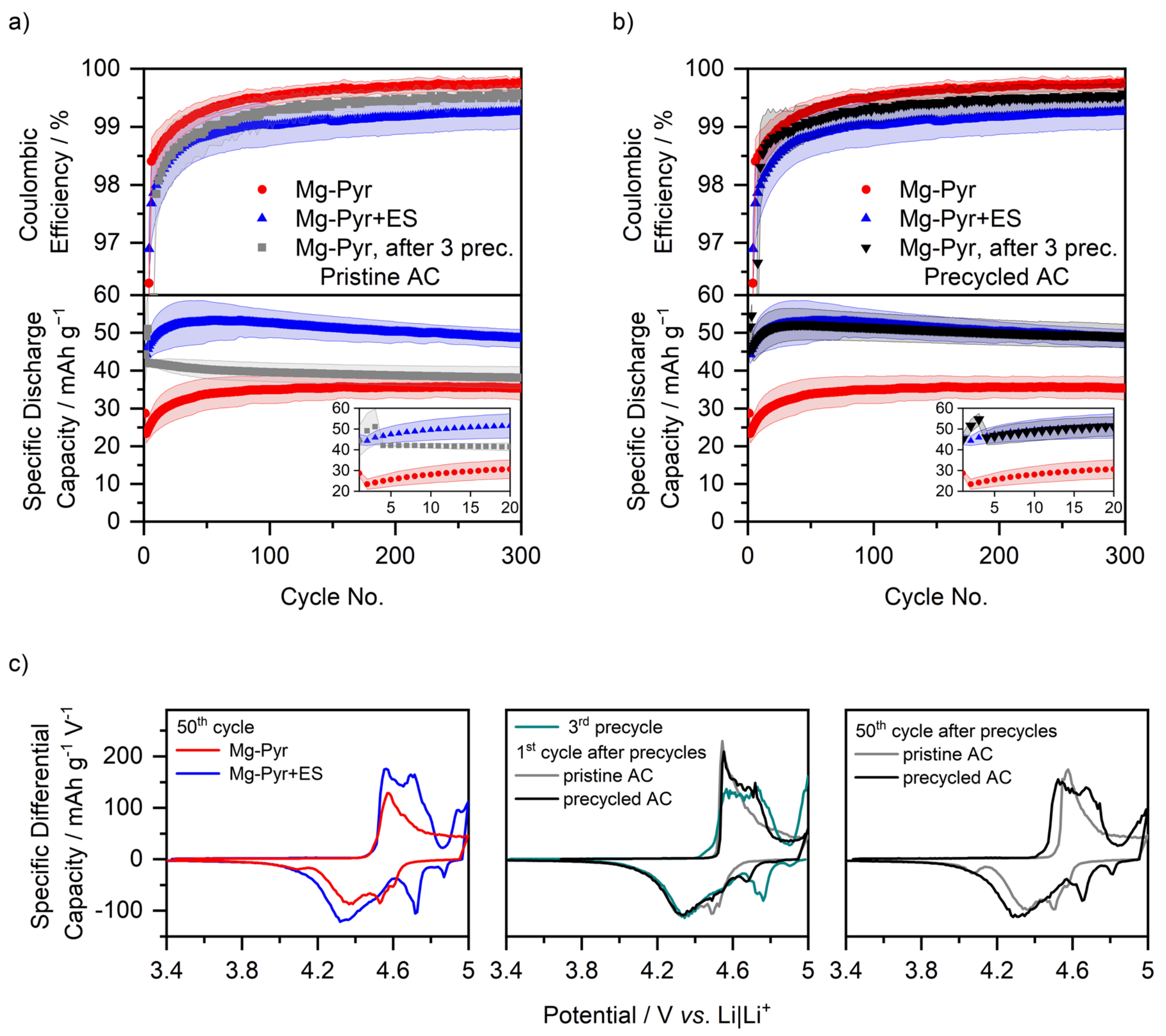

Figure S13. a) and b) Coulombic efficiency and specific discharge capacity (including a magnification of the first 20 cycles) of graphite ‖ AC Swagelok-type cells (three-electrode configuration; QRE: Li metal) with 0.5 M $Mg(TFSI)_2$ in $Pyr_{14}TFSI$ (red), 0.5 M $Mg(TFSI)_2$ in $Pyr_{14}TFSI$ + 2 wt.% ES (blue) at 100 mA g^{-1} (1 pre-cycle at 10 mA g^{-1}) and 0.5 M $Mg(TFSI)_2$ in $Pyr_{14}TFSI$ with pre-cycled graphite and a) pristine AC (grey) or b) pre-cycled AC (black) at 100 mA g^{-1} (3 pre-cycles with Mg-Pyr+ES at 10 mA g^{-1}) with cut-off potentials of 3.4 V and 5.0 V *vs.* Li|Li^+. c) The corresponding differential capacity *vs.* potential plots of the 50th cycle (Mg-Pyr and Mg-Pyr+ES) and the 3rd pre-cycle with Mg-Pyr+ES (cyan) and first and 50th subsequent cycle with Mg-Pyr using pristine AC (grey) and precycled AC (black).

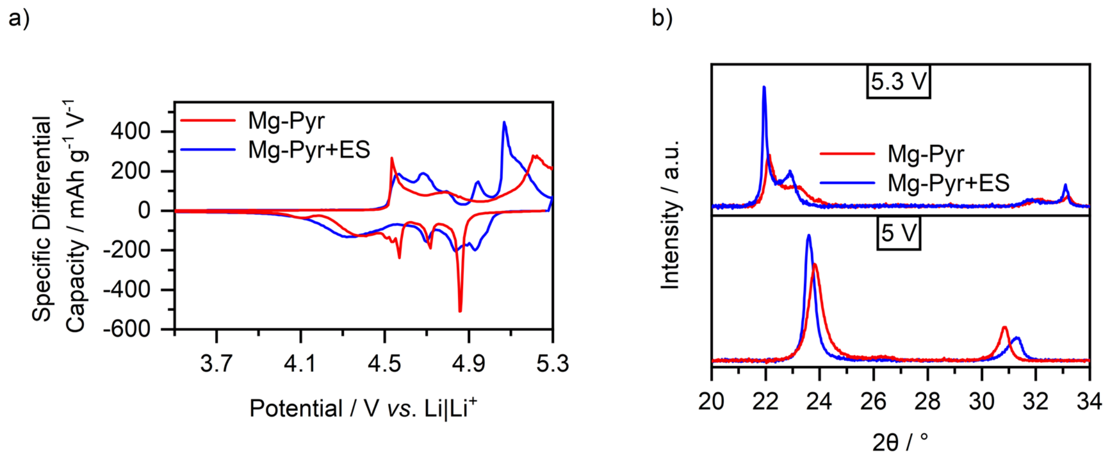

Figure S14. a) Differential capacity *vs.* potential plots of graphite ‖ AC Swagelok-type cells (three-electrode configuration; QRE: Li metal) with 0.5 M $Mg(TFSI)_2$ in $Pyr_{14}TFSI$ (red) and 0.5 M $Mg(TFSI)_2$ in $Pyr_{14}TFSI$ + 2 wt.% ES (blue) at 100 mA g^{-1} with cut-off potentials of 3.4 V and 5.3 V *vs.* Li|Li⁺. b) XRD patterns of graphite electrodes charged in 0.5 M $Mg(TFSI)_2$ in $Pyr_{14}TFSI$ (red) and 0.5 M $Mg(TFSI)_2$ in $Pyr_{14}TFSI$ + 2 wt.% ES (blue) at 100 mA g^{-1} to 5.3 V *vs.* Li|Li⁺ (upper part) and 10 mA g^{-1} to 5.0 V *vs.* Li|Li⁺ (lower part).

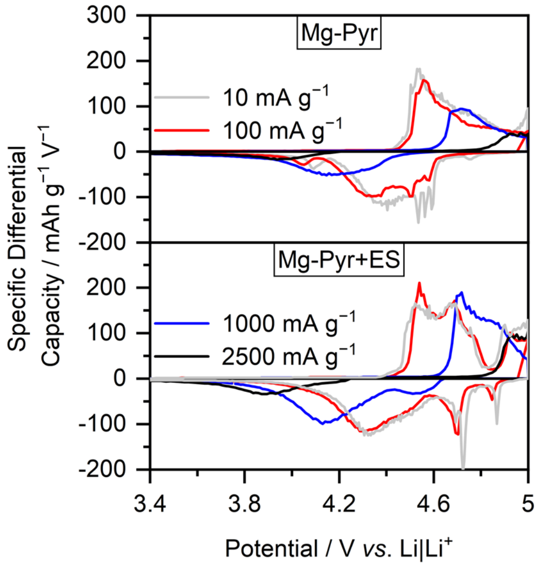

Figure S15. Differential capacity *vs.* potential plots of graphite ‖ AC Swagelok-type cells (three-electrode configuration; QRE: Li metal) with 0.5 M $Mg(TFSI)_2$ in $Pyr_{14}TFSI$ (upper part) and 0.5 M $Mg(TFSI)_2$ in $Pyr_{14}TFSI$ + 2 wt.% ES (lower part) at 10 mA g^{-1} (grey) 100 mA g^{-1} (red), 1000 mA g^{-1} (blue), and 2500 mA g^{-1} (black) with cut-off potentials of 3.4 V and 5.0 V *vs.* Li|Li⁺.

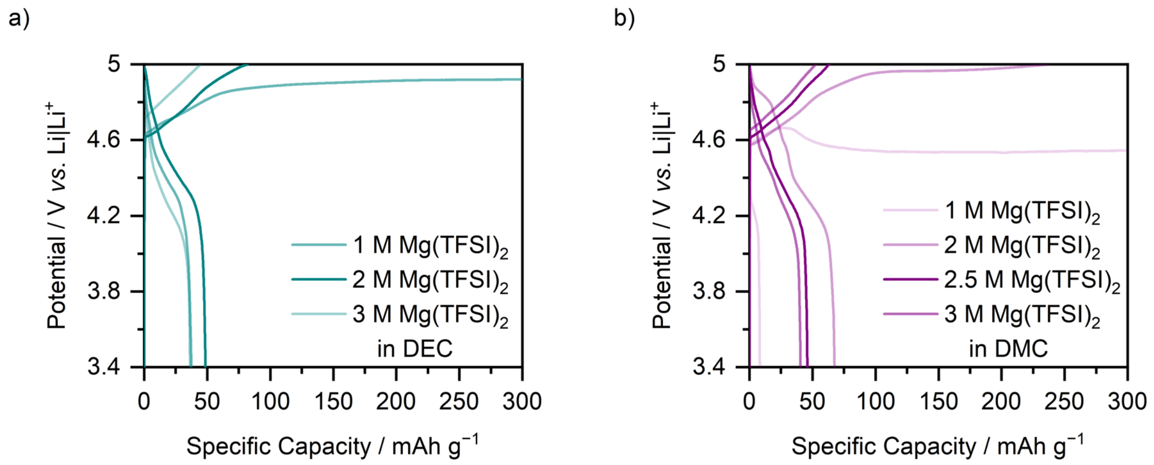

Figure S16. Potential *vs*. specific capacity of the first cycle of graphite ‖ AC Swagelok-type cells (three-electrode configuration; QRE: Li metal) with $Mg(TFSI)_2$ in a) DMC and b) DEC with various concentrations at 10 mA g^{-1} with cut-off potentials of 3.4 V and 5.0 V *vs*. $Li|Li^+$.

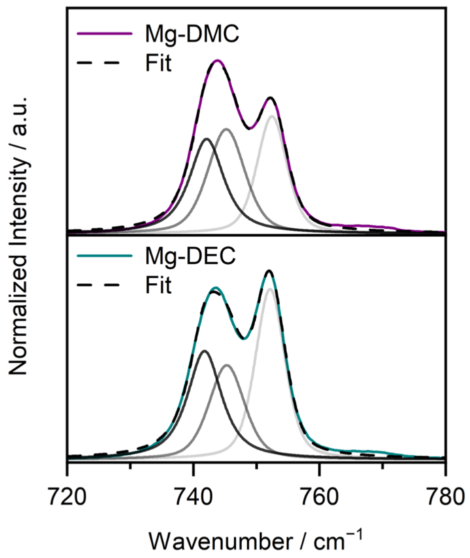

Figure S17. Raman spectra of 2.5 M $Mg(TFSI)_2$ in DMC (purple) and 2 M $Mg(TFSI)_2$ in DEC (cyan) between 720 and 780 cm^{-1} including the fitted (pseudo Voigt) spectra. The fitted spectra were normalized according to the maximal intensity of the band at ~743 cm^{-1} of the experimental spectra.

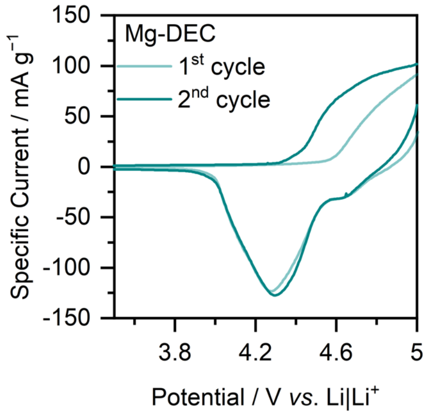

Figure S18. Cyclic voltammogram of the 1st and 2nd cycle of modified graphite ‖ AC pouch-type cells (three-electrode configuration; RE/QRE: Li metal scan speed: 0.5 mV s^{-1}, cut-off potentials of 3.4 to 5.0 V *vs*. Li|Li^+) of *in situ* Raman measurements (Figure 11) with Mg-DEC.

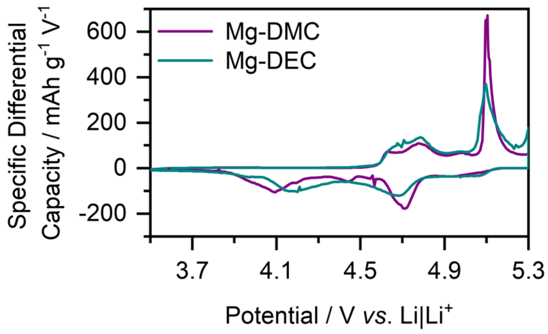

Figure S19. Differential capacity *vs*. potential plots of graphite ‖ AC Swagelok-type cells (three-electrode configuration; QRE: Li metal) with 2.5 M $Mg(TFSI)_2$ in DMC (purple) and 2 M $Mg(TFSI)_2$ in DEC (cyan) at 100 mA g^{-1} with cut-off potentials of 3.4 V and 5.3 V *vs*. Li|Li^+.

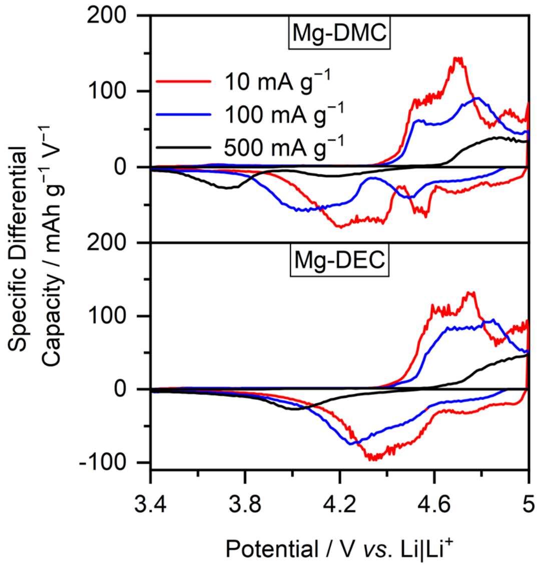

Figure S20. Differential capacity *vs*. potential plots of graphite ǁ AC Swagelok-type cells (three-electrode configuration; QRE: Li metal) with 2.5 M $Mg(TFSI)_2$ in DMC (upper part) and 2 M $Mg(TFSI)_2$ in DEC (lower part) at 10 mA g^{-1} (red) 100 mA g^{-1} (blue) and 500 mA g^{-1} (black) with cut-off potentials of 3.4 V and 5.0 V *vs*. Li|Li^+.

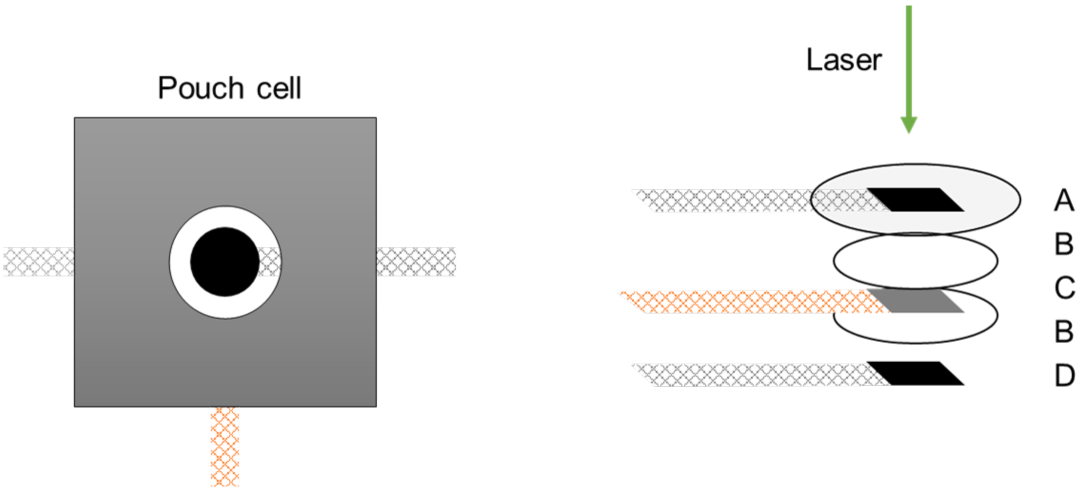

Figure S21. Schematic illustration of the *in situ* pouch cell design. A: Graphite composite electrode with an Al mesh current collector casted on a glass window. B: GF/A separator soaked with electrolyte. C: Li metal reference electrode on carbon-coated copper mesh. D: AC reference electrode with an Al mesh current collector.
